# Using Copy Number Variation Data and Neural Networks to Predict Cancer Metastasis Origin Achieves High Area under the Curve Value with a Trade-Off in Precision

**DOI:** 10.3390/cimb46080490

**Published:** 2024-08-01

**Authors:** Michel-Edwar Mickael, Norwin Kubick, Atanas G. Atanasov, Petr Martinek, Jarosław Olav Horbańczuk, Nikko Floretes, Michael Michal, Tomas Vanecek, Justyna Paszkiewicz, Mariusz Sacharczuk, Piotr Religa

**Affiliations:** 1 Institute of Genetics and Animal Biotechnology, Polish Academy of Sciences, Postepu 36A, 05-552 Jastrzebiec, Polandj.horbanczuk@igbzpan.pl (J.O.H.); m.sacharczuk@igbzpan.pl (M.S.); 2 Department of Biology, Institute of Plant Science and Microbiology, University of Hamburg, Ohnhorststr. 18, 22609 Hamburg, Germany; kubick.norwin@googlemail.com; 3 Ludwig Boltzmann Institute Digital Health and Patient Safety, Medical University of Vienna, Spitalgasse 23, 1090 Vienna, Austria; 4 Department of Pathology, Biopticka Laboratory s.r.o., Mikulasske nam. 4, 326 00 Plzen, Czech Republic; martinek@biopticka.cz (P.M.); michael.michal@biopticka.cz (M.M.); vanecek@biopticka.cz (T.V.); 5 College of Engineering, Samar State University, University Access Rd, Catbalogan City 6700, Philippines; nikko.floretes@ssu.edu.ph; 6 Department of Health, John Paul II University of Applied Sciences, Sidorska 95/97, 21-500 Biala Podlaska, Poland; j.paszkiewicz@dyd.akademiabialska.pl; 7 Department of Pharmacodynamics, Faculty of Pharmacy, Medical University of Warsaw, Banacha 1B, 02-091 Warsaw, Poland; 8 Department of Medicine, Karolinska Institute, Visionsgatan 18, 171 76 Solna, Sweden

**Keywords:** copy number variant, Ai, CNV

## Abstract

The accurate identification of the primary tumor origin in metastatic cancer cases is crucial for guiding treatment decisions and improving patient outcomes. Copy number alterations (CNAs) and copy number variation (CNV) have emerged as valuable genomic markers for predicting the origin of metastases. However, current models that predict cancer type based on CNV or CNA suffer from low AUC values. To address this challenge, we employed a cutting-edge neural network approach utilizing a dataset comprising CNA profiles from twenty different cancer types. We developed two workflows: the first evaluated the performance of two deep neural networks—one ReLU-based and the other a 2D convolutional network. In the second workflow, we stratified cancer types based on anatomical and physiological classifications, constructing shallow neural networks to differentiate between cancer types within the same cluster. Both approaches demonstrated high AUC values, with deep neural networks achieving a precision of 60%, suggesting a mathematical relationship between CNV type, location, and cancer type. Our findings highlight the potential of using CNA/CNV to aid pathologists in accurately identifying cancer origins with accessible clinical tests.

## 1. Introduction

Metastasis is one of the critical factors affecting life expectancy in cancer patients [1]. Metastatic cancer cells spreading to distant sites significantly affect patients’ prognosis and limit treatment effectiveness [2]. Identifying the origin of metastatic cancer cells is essential to prevent further spreading of said cancerous cells [3]. Failure to correctly pinpoint the origin of cancer can significantly reduce survival rates, as seen in cases of cancer with unknown primary sites [4]. Determining the primary tumor site allows for tailored treatment strategies, aligning therapies with the specific characteristics of the cancer. This, in turn, contributes to enhanced patient outcomes. Furthermore, accurately detecting the origin of metastasis can help uncover the complex mechanisms driving the spread of cancer cells. This understanding can aid in developing new interventions aimed at limiting metastasis.

Predicting cancer using copy number alterations (CNAs) could be instrumental in tracking the origin of metastases [5]. CNA refers to genomic changes in cancer cells, where specific regions have an altered number of copies compared to normal cells. As cancer spreads and metastasizes, it leaves distinct CNA patterns that can be used to trace the primary tumor site. Using single-cell RNA sequencing (scRNA-seq), it has been demonstrated that there is a high degree of similarity between primary tumors and their metastases, suggesting clonality of CNA fingerprints in cancer [6]. Several pieces of evidence support this hypothesis. For example, investigation of intratumor heterogeneity in lung adenocarcinomas using multiregional sequencing revealed a high degree of conservation of CNAs across different tumor regions, supporting clonality and the retention of genetic alterations during metastasis [7,8]. Analyzing the CNA profiles of metastatic tumors could provide valuable insights into the origin and spread of the disease, aiding treatment decisions, personalized medicine approaches, and targeted therapies for improved patient outcomes.

Advancements have been made in using copy number alterations (CNAs) to track the origin of metastases; however, further improvements are needed. Ding et al. analyzed recurrent CNVs from non-tumor blood cell DNAs of non-cancer subjects and identified differences in copy number losses and gains between cancer patients and controls in hepatocellular carcinoma, gastric cancer, and colorectal cancer [9]. Ning et al. utilized CNVs of 23,082 genes to classify six different types of cancers, achieving 75% accuracy by reducing the feature space to CNVs of 19 genes [10]. Similarly, Sanaa et al. trained seven machine learning classifiers using the same dataset, with the random forest algorithm achieving 86% accuracy [11]. 

These studies highlight the potential of CNVs and CNA analysis in predicting cancer risk, differentiating cancer types, and providing genetic insights. Recently, Karim et al. collected CNV data from The Cancer Genome Atlas, including genomic deletions and duplications, for 8000 cancer patients covering 14 different cancer types. They employed sparse representations based on oncogenes and protein-coding genes, training Conv-LSTM and convolutional autoencoder (CAE) networks to capture important features and initialize weights for subsequent convolutional layers (Karim et al., [5]). However, the highest accuracy their approach could reach is 75%. These recent efforts contribute to further advancing the field and addressing the need for improvement in CNA analysis for metastasis tracking, but they also emphasize the necessity for further enhancements.

In this report, we trained several neural networks using CNA data from twenty different cancer types. The input to the network consisted of the CNA type and the gene locus (i.e., chromosome, start, end, and strand). We explored the performance of two workflows. In the first, we compared the performance of two generalized models. In the second, we built individual models based on specific cancer types, namely male- and female-specific cancers, brain cancers, cancers of the excretory system, and cancers of the digestive system. Overall, the models achieved comparable AUC values of 0.9. However, upon closer examination of the models’ performances, the maximum precision they can achieve is 60%. This AI-based approach shows promise for improving metastatic cancer diagnosis and treatment planning. Further research is necessary to enhance the accuracy and reliability of CNA-based predictions. Nonetheless, our study highlights the potential of AI and genomic profiling to offer valuable insights into the origin and progression of metastatic cancer, paving the way for more targeted and effective therapeutic strategies.

## 2. Methods

### 2.1. Database Construction and Preprocessing

**Figure 1:**
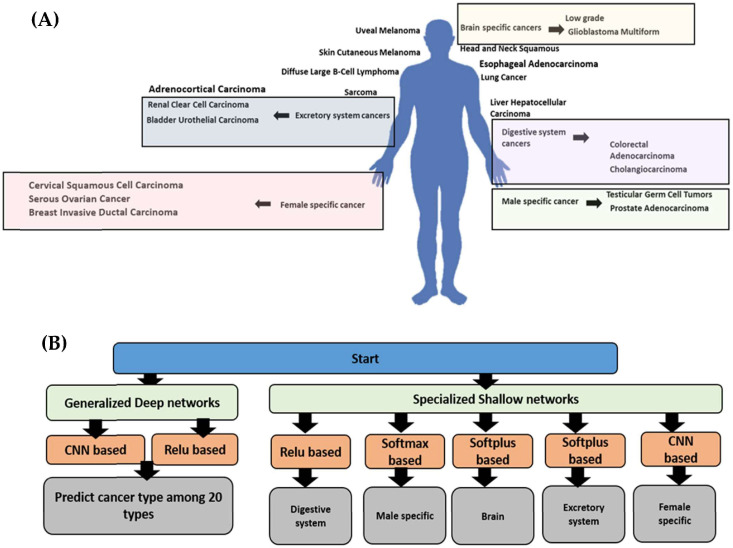
Workflow of the AI modeling. (**A**) The dataset included 20 types of cancer. (**B**) We employed two workflows: generalized deep networks and specialized shallow networks. In the first workflow, we explored the performance of two different models, namely a CNN-based network and a ReLU-based network. In the specialized shallow networks workflow, we built shallow models that differentiate between cancer types within the same system or context. For example, the digestive system model aims to distinguish between two cancers, namely cholangiocarcinoma and colorectal adenocarcinoma.

We downloaded 20 types of cancer genotypes derived from 13,000 patients through the cBioPortal portal [12]. The data originated from a study encompassing 32 different studies and were deposited in TCGA, PanCancer Atlas (Table 1) (Figure 1) [12,13]. After downloading the data, we filtered out lower-frequency genes using a threshold of 6% [14]. Subsequently, duplicates were removed based on the highest frequency for any given gene. Following this filtration process, the final dataset contained 7058 records organized into 20 cancer types. Using the gene names, we retrieved chromosome location details, including coordinates (start, end) and strand information, using a customized Python-based analysis of the GRCh38 genome [15]. For the AI model input, the chromosome number, start and end coordinates, strand information, and the type of CNA (2 different types) were used, resulting in 6 input states in total. The two states of the CNA type were encoded using a one-hot encoded vector in Python3.12. For cross-validation, we utilized the automatic cross-validation option in the Keras fit function with a validation ratio of 0.39.

### 2.2. Model Construction

In the first workflow, after downloading the data, we normalized the chromosome values (start, end, and strand). The CNA values were encoded using a one-hot encoder to represent two different cases. Similarly, the cancer types were numerically encoded to represent each cancer type uniquely. Several architectures were compared using the Keras sequential pipeline. In the second workflow, we grouped types of cancer based on their locations within the human body. For example, low-grade gliomas and glioblastoma multiforme were both categorized as brain cancers. Both models were developed in a Google Colab notebook using Python 3. The code for differentiating between cancer types based on copy number alterations (CNAs) is available on GitHub at https://github.com/michel-phylo/Adera3 (accessed on 21 July 2024).

#### 2.2.1. The Generalized Model 

For the first model, we experimented with several architectures and optimizers, using cross-entropy as the loss function. We employed Softmax as the final layer to generate probability predictions for the 20 types of cancer. Optimization was carried out using either the ADAM or ADAMAX optimizer with the categorical cross-entropy option. The main architectures explored were the following: (a) convolution + ReLU and (b) SELU + ReLU + ELU. The SELU model utilized multiple sequential layers within the Keras framework (Table 2).

#### 2.2.2. The Specialized Models

For the specialized models, we employed binary classification either with a single layer classifier or a classifier combined with a ReLU layer. Subsequently, we calculated accuracy and loss. Internal validation was conducted using the train–test split method, dividing the database in a 3:2 ratio (Table 3).

### 2.3. Hyperparameter Tuning

To find the optimal values for each model, we used two methods: (a) Keras hyperparameter tuning and (b) grid search. In both cases, our optimization aimed to minimize validation loss and maximize validation accuracy. The parameters investigated included learning rate, clip norm, and batch size.

### 2.4. Cross-Validation Using Synthetic Dataset and Sensitivity Threshold Setting

We constructed a synthetic database to mirror the original training data. This involved generating data with a similar statistical distribution for chromosomes, CNA values, and cancer types. Sensitivity was calculated as the number of correct predictions divided by the total number of predictions. We conducted three separate runs, each producing three different values randomly selected from the dataset.

For the sensitivity analysis, we conducted a grid search to determine the optimal threshold that maximizes precision and recall. The threshold was selected based on overall accuracy and the threshold value that assigns a corresponding probability to each category.

### 2.5. Biological Explainability Analysis

We conducted a biological explainability analysis to understand the model’s decision-making process. This analysis involved two levels of investigation. In the first level, we utilized all possible unique combinations of inputs grouped into single, double, triple, or quadruple groups without altering the model’s architecture. The output of this analysis included the model’s accuracy, precision, recall, and F1 score. This approach aimed to assess the sensitivity of the model to its inputs and infer the weights assigned to each input. In the second level of analysis, we modified the generalized model architecture by incorporating dense layers with the ReLU activation function or a convolution layer. This analysis focused on individual model inputs. The output of this procedure indicated changes in the model’s accuracy, aiming to identify biological inputs detected by the two utilized model layers.

### 2.6. Explainability Analysis Using SHAP in TensorFlow

To evaluate how input parameters impact model prediction accuracy across diverse cancer types, SHAP (SHapley Additive exPlanations) analysis was employed. This method effectively assesses feature importance by quantifying the influence of each parameter on model predictions. The input parameters encompassed six key features: amplification, deletion, chromosome number, start coordinates, end coordinates, and strand orientation. SHAP analysis was independently performed for each cancer type under investigation, computing SHAP values to measure the relative impact of features 0 through 5 on model predictions. The primary objective of the analysis was to identify which features significantly contributed to enhancing model prediction accuracy across the different cancer types studied.

## 3. Results

**Figure 2:**
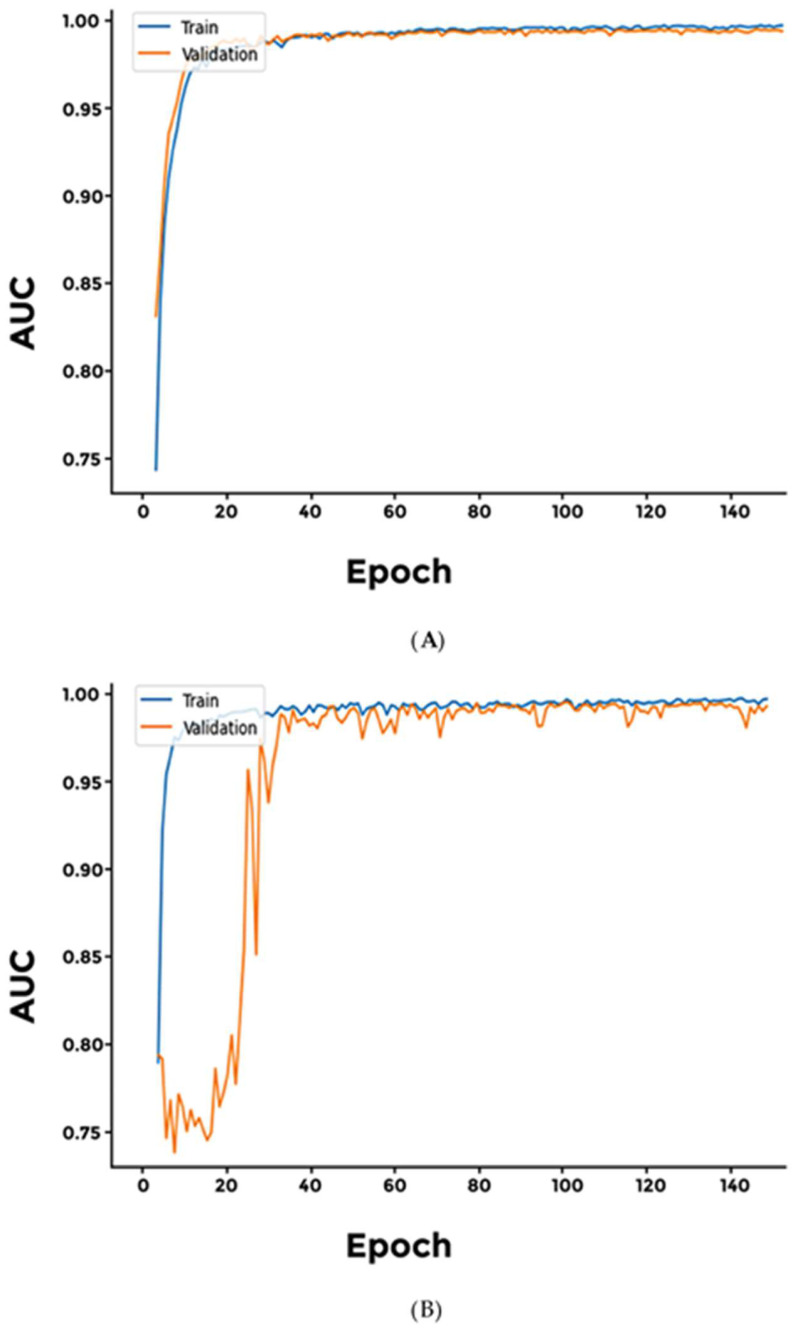
Comparison of AUC values between the two generalized models. Both models, (**A**) CNN and (**B**) SELU, achieved significant AUC values.

We evaluated two distinct architectures for our model: a 2D convolution-based approach and a ReLU-based architecture. Both models demonstrated an AUC (area under the curve) value of 0.9, accompanied by a loss of less than 0.5 (Figure 2 and Figure S1). 

**Figure 3:**
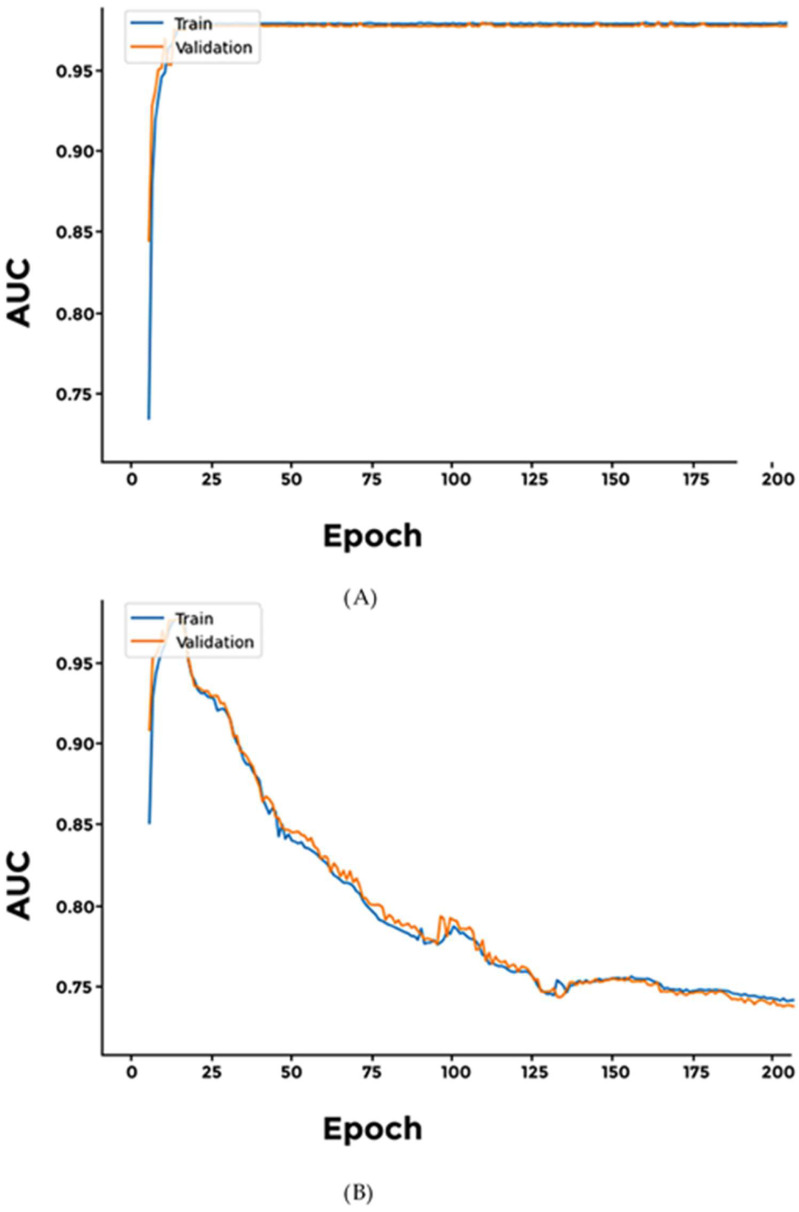
Comparison of Male-Specific Models’ AUC Values. (**A**) Softmax; (**B**) Softplus. Softmax outperformed Softplus in terms of AUC values.

For classification of male-specific cancers, we experimented with three models: (a) Softmax, (b) Sigmoid, and (c) Softplus. Overall, all three models achieved high accuracy with very low loss. However, the sensitivity values and the AUC values for the Softplus model were significantly lower than the other models investigated (Figure 3 and Figure S2).

**Figure 4:**
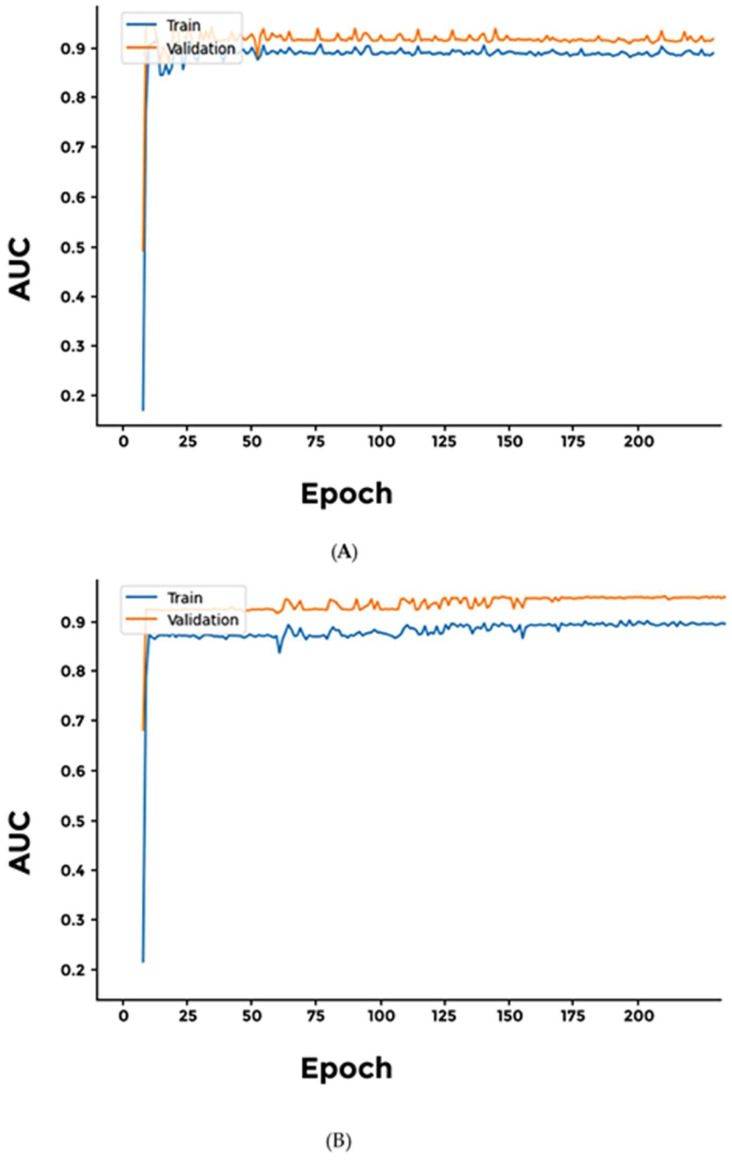
Performance of different networks for differentiating between two brain cancer types. (**A**) Sigmoid; (**B**) Softplus. AUC values indicate that after initial rapid learning, reaching almost 90%, all models failed to converge, indicating possible overfitting.

We experimented with three models: (a) Softmax, (b) Sigmoid, and (c) Softplus for classification of brain cancers. The Sigmoid model achieved the highest accuracy, while the Softplus model’s performance was limited, suffering the highest loss. Additionally, we observed a consistent difference between the training and validation AUC in all three models, indicating it did not converge effectively (Figure 4 and Figure S3).

**Figure 5:**
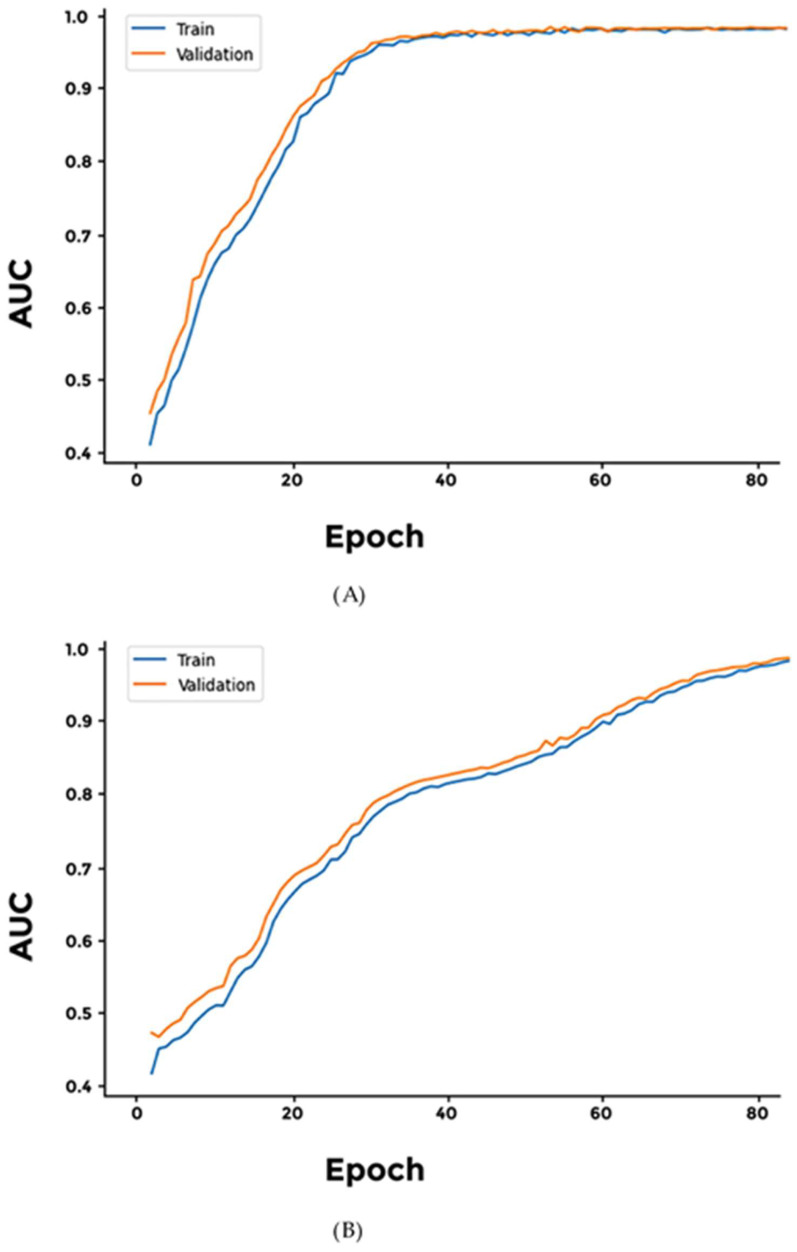
Performance of specialized models differentiating between two types of excretory system cancers. (**A**) Softmax; (**B**) Softplus. There appears to be no significant difference between the performances of these models.

We investigated binary classification of two types of excretory system cancers, namely kidney renal clear cell carcinoma and bladder urothelial carcinoma. All three investigated models achieve similar results (Figure 5 and Figure S4). 

**Figure 6:**
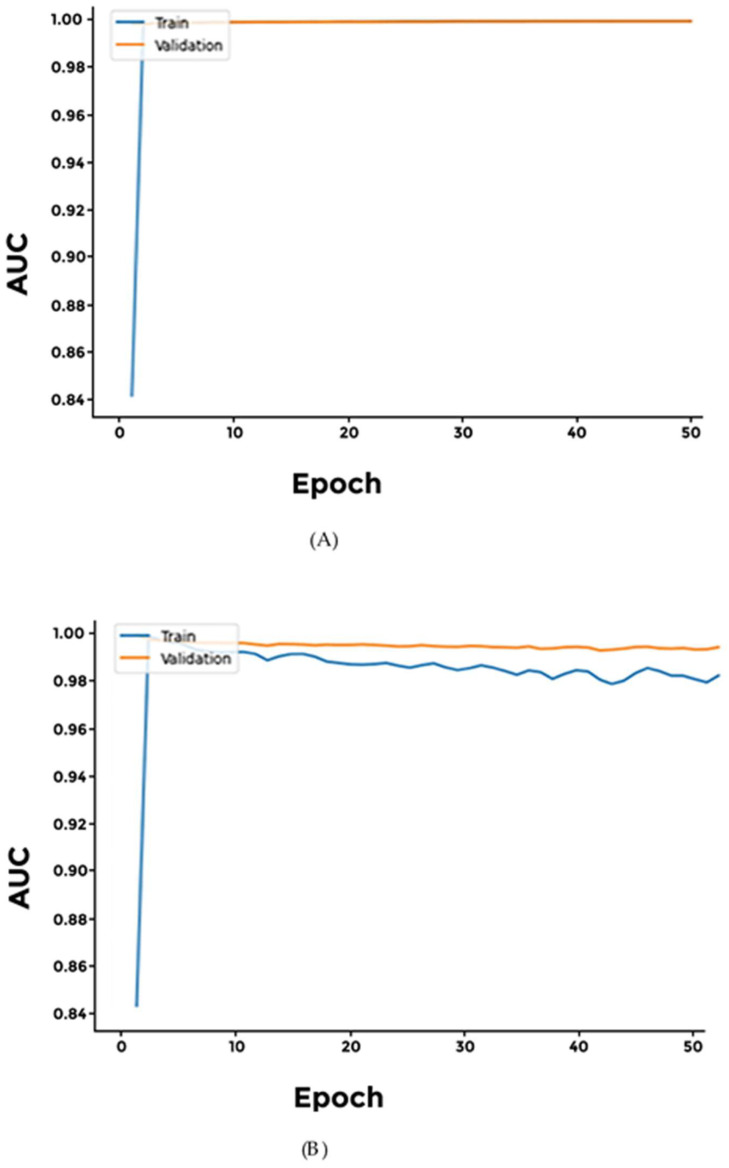
Performance of different networks for classifying two digestive system cancers. (**A**) Softmax; (**B**) Softplus. While the Softmax network achieves significant accuracy and AUC values, the Softplus model appears to be overfitting.

We built three different models using Sigmoid, Softmax, and Softplus networks to classify digestive system cancers. To increase accuracy, we augmented the networks with a dense layer with ReLU activation. It is interesting to note that the Softplus network AUC values do not seem to converge, hinting that this particular network suffers from overfitting (Figure 6 and Figure S5).

**Figure 7:**
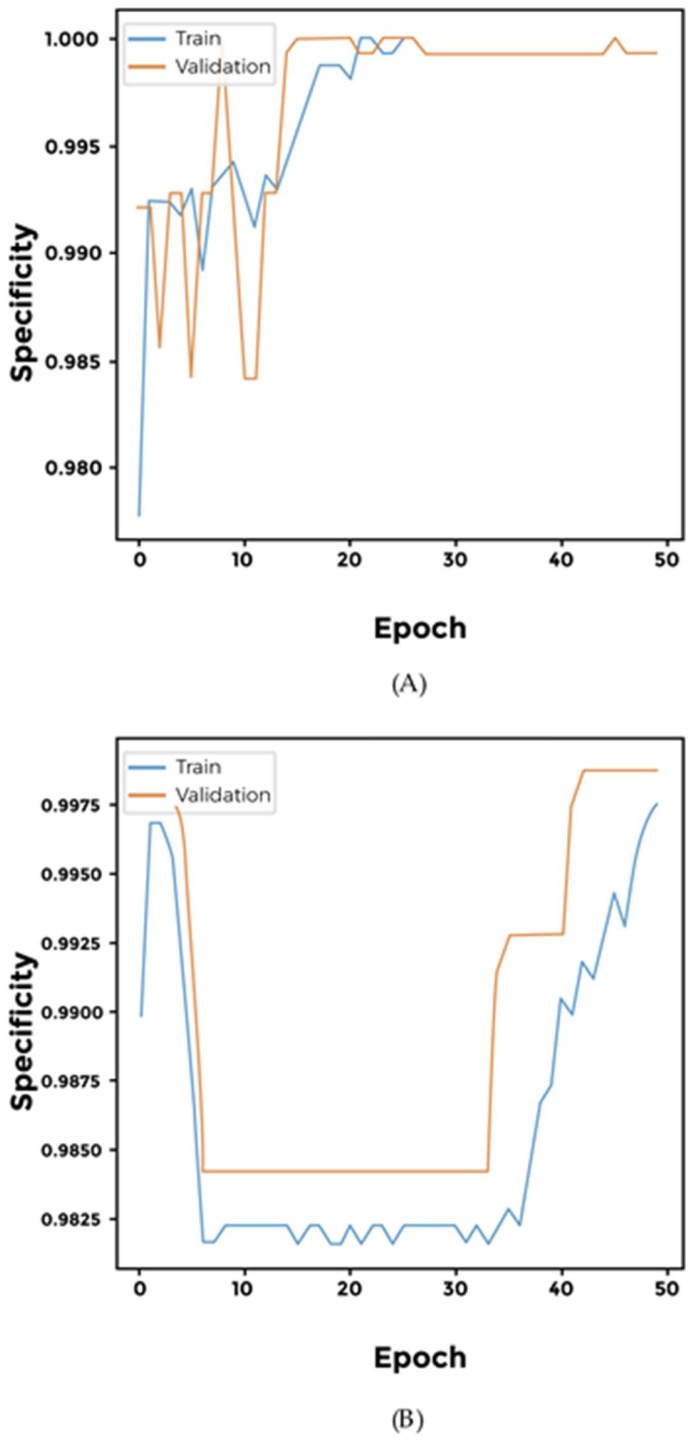
Performance of different networks for differentiating between two female-specific cancer types. (**A**) Softmax; (**B**) Softplus. Interestingly, the specificity at a sensitivity of 0.5 for the Softplus model indicates that there were fluctuations and potential overfitting during training. However, both training and validation specificities improved significantly towards the end.

We used our pipeline to classify two female-specific cancers namely; Cervical Squamous Cell Carcinoma and Ovarian Serous Cystadenocarcinoma. Our results indicate that our three networks; Sigmoid, Softmax, and Softplus achieved comparable results (Figure 7 and Supplementary Figure S6). 

**Figure 8:**
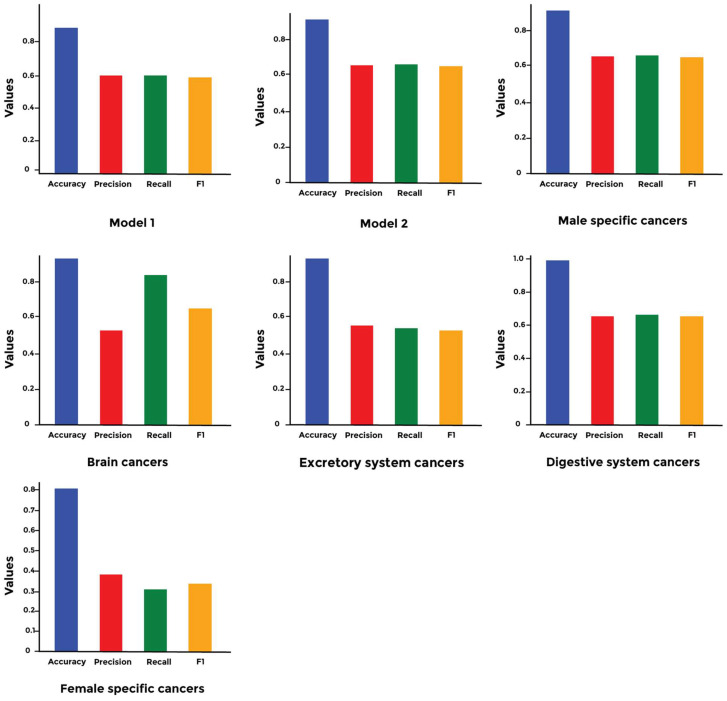
Comparison of the models’ performance. We calculated accuracy, precision, recall, and F1 score for each of the models studied. While all the models achieved an accuracy higher than 80%, none of them achieved a precision higher than 60%. However, the brain cancer model achieved an 80% recall.

Next, we validated the models’ performance by calculating accuracy, precision, recall, and F1 score for each model using a customized evaluation command in TensorFlow. Our analysis did not detect significant differences in the performance between generalized models 1 and 2. However, variations were observed in the performance of the specialized models, with the female-specific model performing worse than the others (see Figure 8).

**Figure 9:**
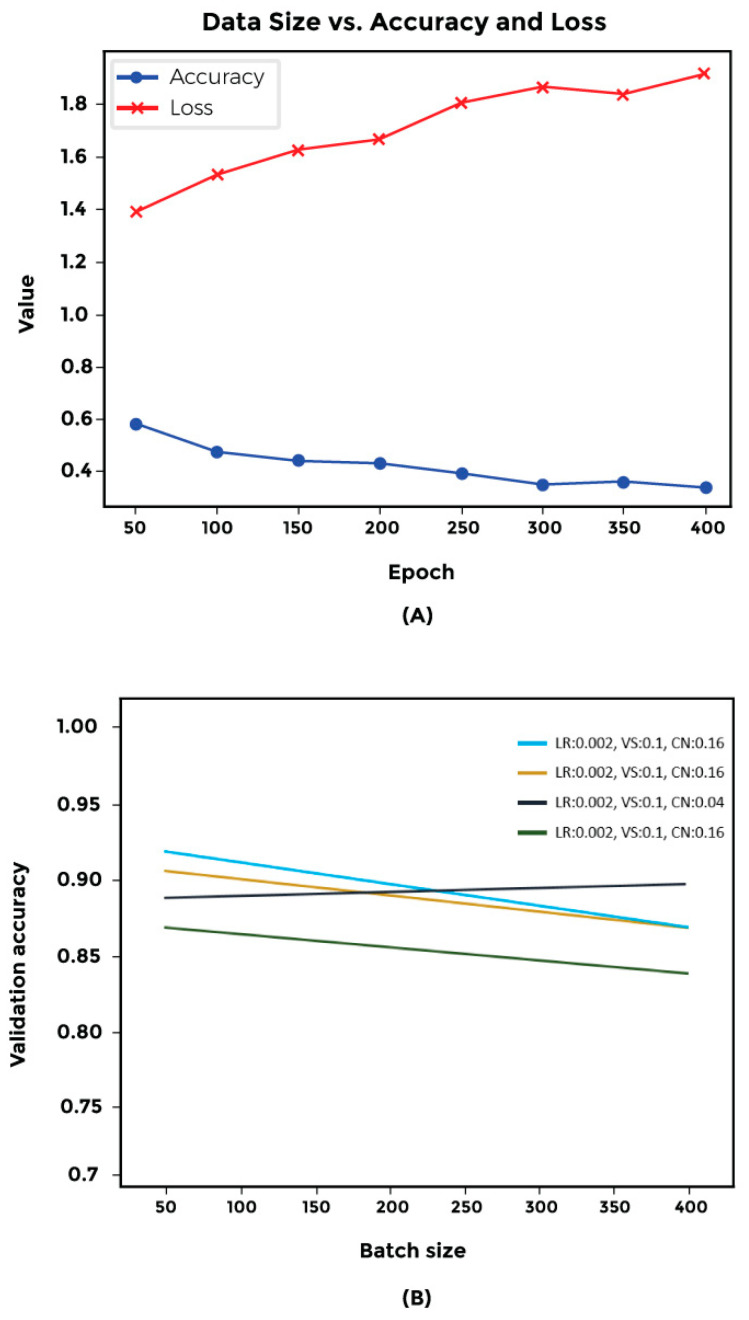
Effects of parameter fine-tuning. We explored the impact of tuning parameters using a grid search and Keras tuner. (**A**) Regarding batch size, our findings suggest that reducing the batch size can enhance accuracy. (**B**) Analysis of the hyperparameter involving learning rate, clip norm, and validation split indicates that each parameter can profoundly affect accuracy.

We used a grid search algorithm to find the parameters that would maximize the accuracy of classification and minimize loss for the generalized networks. The parameters investigated included learning rate, clip norm, and batch size. Our results indicate that the optimal configuration consists of a batch size of 50, a learning rate of 0.002, a clip norm of 0.08, and a validation split of 0.1. This combination yields over 92% accuracy and less than 0.28 loss (Figure 9).

**Figure 10:**
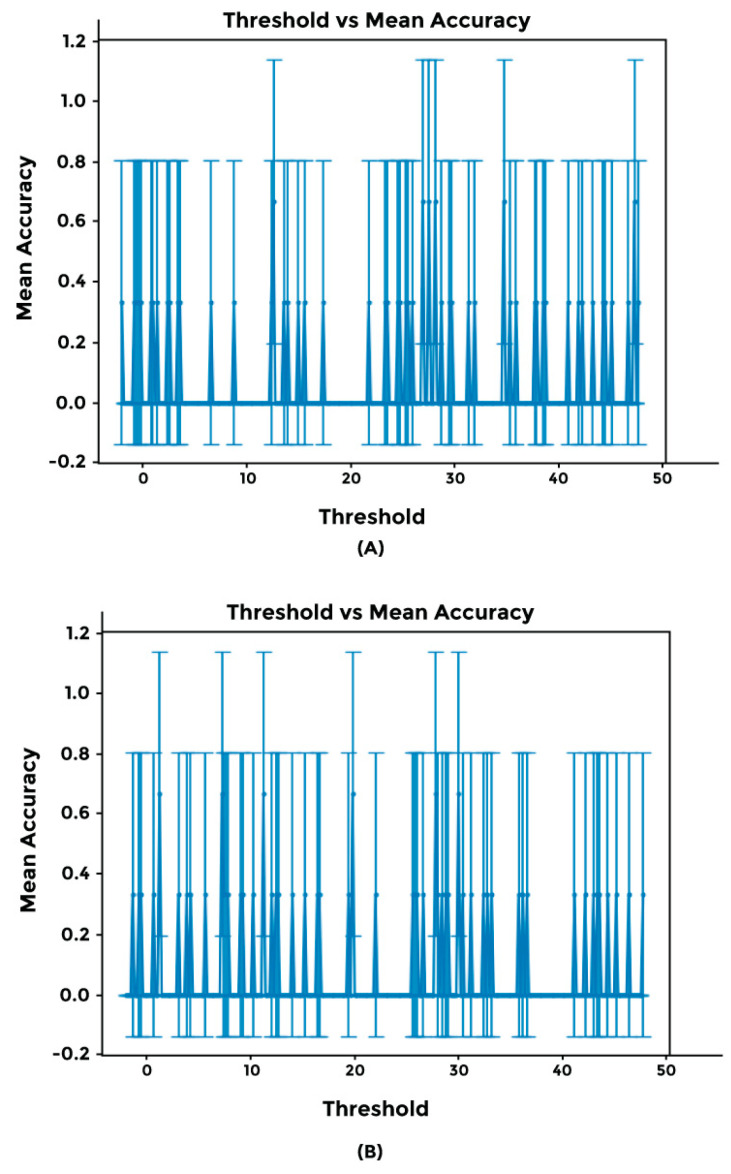
Examining the impact of threshold on accuracy. Both models exhibit multiple peaks, with potential accuracies reaching up to 99%. However, both models also show a high standard deviation.

We compared the performance of our two generalized models using an in-house synthetic dataset. This dataset mirrors the essence of the original dataset, focusing on chromosome location, strand, CNA values, and cancer type. To identify the optimal threshold for accepting a prediction as a tag for a specific cancer type, we analyzed the threshold versus mean accuracy relationship. Both versions of model 1 (with and without the Conv2D network) achieved varying accuracies, with a maximum of 75% at different threshold levels (Figure 10).

**Figure 11:**
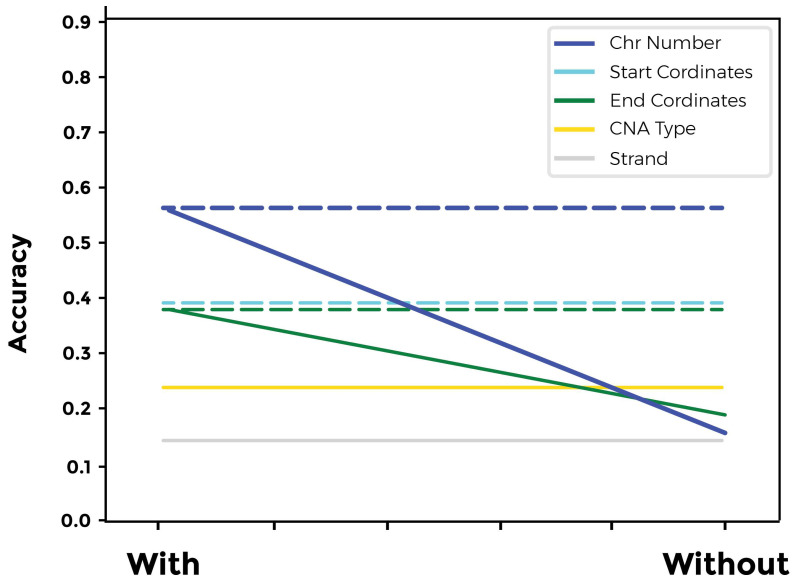
Biological explainability analysis of the generalized model performance. Our results indicate that the gene locus parameters have a larger impact on model accuracy in comparison to CNA type. Model accuracy is more dependent on the dense layer with the ReLU activation function than on the convolution network layers (solid line represents ReLU layers and dashed line represents the convolution layer).

We conducted a two-level biological explainability analysis. On the first level, we compared the impact of all available input combinations on model accuracy without altering the model architecture (Supplementary Figure S7). In the second level, we included one of the two primary architectural layers (a convolutional layer or a dense layer with ReLU activation) and assessed model accuracy using a single input (Figure 11). At the first level, we discovered that the chromosome number parameter had the greatest effect on network accuracy, whereas the strand had the least effect. The start and end coordinate parameters had a similar impact on the overall model accuracy. Regarding the investigation into layer effects, we found that the model’s performance is more sensitive to dense layers with ReLU activation compared to convolutional layers.

**Figure 12:**
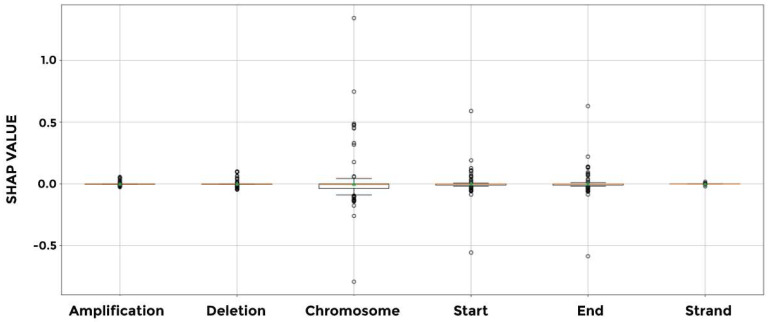
Box plot of the features based on their respective SHAP values. The chromosome number parameter is the most important parameter in determining the model’s performance. The results indicate that the chromosome parameter is the most influential in the model’s decision-making process, as evidenced by its larger variation and numerous outliers. The start and end parameters also contribute to the model’s predictions to a lesser extent. Amplification, deletion, and strand have the least impact, with their SHAP values being consistently close to zero.

Next, we conducted a SHAP analysis to estimate the contribution of each of the input parameters to the model prediction accuracy for each of the cancer types investigated. Results show similar findings to the previous analysis, where the chromosome number (Feature 2) is the most significant parameter in determining the SHAP values in various cancer types. This is followed by the end coordinates (Feature 4) and, to a lesser extent, the start coordinates (Feature 3). The amplification (Feature 0), deletion (Feature 1), and strand (Feature 5) features have the least impact on determining the SHAP values (Figure 12).

## 4. Discussion

In the quest for advancing cancer diagnosis and personalized treatment, identifying the precise origin of cancer becomes paramount. Cancer is a complex disease with diverse manifestations, and determining its primary site can significantly impact patient care and treatment outcomes. Chromosomal copy number alteration (CNA) data, offering insights into genetic changes, have emerged as valuable tools in unraveling the intricate molecular landscape of cancer. By harnessing the power of deep learning models, we sought to explore the potential of CNA data in accurately pinpointing the origin of metastatic cancers [16,17]. Our study sheds light on the promise of leveraging computational approaches to revolutionize cancer diagnostics and empower oncologists and pathologists with more reliable and efficient methods for determining cancer origin and guiding tailored therapeutic strategies.

Our generalized deep models can predict cancer type with 90% accuracy; however, their precision values were lower than 60%. In our comprehensive study, we explored two generalized models: Conv2D+ReLU and SELU+ReLU, both culminating with the Softmax activation function. The Conv2D+ReLU model has been widely used due to its effectiveness in feature extraction and pattern recognition [18,19]. SELU activation has shown promise in reducing vanishing/exploding gradients and promoting self-normalization, enhancing learning in deep neural networks [20]. Prediction of cancer type based on genetic information has recently come under the spotlight. Mostavi et al. used a convolutional network to identify cancer types based on RNA-seq and achieved more than 90% accuracy. However, the RNA-seq data are costly and cannot be used for personalized medicine [21]. Yuan et al. achieved more than 90% on cancer detection using copy number aberration and chromatin 3D structure. However, their approach is not feasible for clinical settings [22]. Karim et al. used an ensemble network which constitutes a combination of a convolutional network and an LSTM. However, the accuracy is lower than 76% [5]. Although our proposed models achieve higher accuracy, they suffer from low precision. This might indicate that the results could be close to the true value but vary widely when the prediction is repeated. To increase the precision of the models, we explored the effect of using smaller ensemble models (specialized shallow models). Interestingly, this did not result in enhancing precision, as there was variation between the different specialized models. To challenge this bottleneck, we explored the effect of balancing the data using customized loss functions such as focal cross-entropy. However, the precision levels remained lower than 60% (Figure 8).

To delve further into this matter, we examined the effects of fine-tuning parameters, including the batch size, learning rate, clip norm, validation split, and prediction threshold. Although fine-tuning these parameters can achieve higher accuracy, models suffered from a high standard deviation, indicating that the precision remained low (Figure 10). We hypothesize that as the cost of genotyping decreases, more datasets will be available for training, thus increasing both accuracy and precision.

To enhance the explainability of our models on a biological level, we conducted two types of input sensitivity analysis: (i) on the level of the whole model and (ii) on the level of the primary layer. For the first analysis, we investigated the sensitivity of the model’s accuracy using combinations of the model input parameters (e.g., single, double, triple, and quadruple inputs) (Figure 10). We found that the model’s accuracy was highly sensitive to the chromosome number, start and end coordinates, and their combinations on the first three levels. On the level of quadruple inputs, the highest accuracy was achieved when including chromosome number, start and end, and the CNA type as inputs. This information indicates that our model places a higher weight on the gene locus than the CNA type. Recently, more than 200 cancer types have been identified [23,24,25]. Reports indicate that an average tumor sample may exhibit 17% genome amplification and 16% deletion, which are significantly higher than non-pathological samples [25,26]. CNA can drive tumor progression in cancer by altering gene expression levels [27].

Chen et al. have shown that alpha-endosulfine (ENSA) exhibits recurrent amplification at the 1q21.3 region and is highly expressed in triple-negative breast cancer [28]. CNA’s role in cancer seems multifactorial and influenced by other aspects such as gene locus [29,30]. Genes located in the middle of a chromosome are less likely to contribute to the genetic variation in traits than genes found at the end [29]. The reason behind this could be the genetic linkage theory, which suggests that genes located in close proximity are evolutionarily linked. The effect of gene linkage in cancer is well documented [31]. The gene locus is also central to various developments inside the cell related to efficient regulation and expression, replication timing, and chromosomal physical interactions, which are highly relevant in cancer development and prognosis. (ii) We compared the model’s sensitivity to the single inputs using the same model architecture, albeit using only dense layers with ReLU activation or a convolution layer. Our analysis indicates that the model’s accuracy is more sensitive to the ReLU layers than the convolution layer. The ReLU layers capture the subtle associations between each input and the cancer type more than the convolution layer. The convolution layers are not connected to the entire input but to smaller sections, while each neuron in the ReLU layer is connected to the entire input, which allows it to build a connection between the gene locus and the CNA type.

Our research is not without limitations. Notably, we used a synthetic dataset for validation. We expect that the accuracy could decrease significantly upon validating our model using a real clinical dataset. Also, the input data are heavily unbalanced. This resulted in high accuracy but low precision. Nevertheless, our study represents a proof of concept in using AI to correlate genotyping to metastasis origin. It specifically highlights the importance of gene locus coordinates, such as the chromosome number, start, and end, that could influence the metastasis type. Further research is needed to determine the dimensions of this interaction.

## 5. Conclusions

This research article highlights the significance of CNV as a valuable genomic marker for the accurate identification of the primary tumor origin in metastatic cancer cases. Utilizing advanced AI-based techniques, this study achieved an impressive accuracy of over 90%; however, it suffers from low precision. As more CNV data become available, we expect that the precision of neural networks in predicting metastasis means that they could be used to support pathologists in the near future.

## Figures and Tables

**Table 1:** Types of cancer and number of samples used in this research.

	Study	Organ	Number of Samples
1	Adrenocortical Carcinoma	Adrenal gland	132
2	Bladder Urothelial Carcinoma	Bladder	612
3	Brain Lower-Grade Glioma	Brain	3
4	Breast Invasive Ductal Carcinoma	Breast	1007
5	Cervical Squamous Cell Carcinoma	Cervix	475
6	Cholangiocarcinoma	Bile duct	212
7	Colorectal Adenocarcinoma	Large intestine	405
8	Diffuse Large B cell lymphoma	B Cells	858
9	Esophageal Adenocarcinoma	Esophagus	905
10	Glioblastoma Multiforme	Brain	39
11	Head and Neck Squamous Cell Carcinoma	Oral cavity, pharynx, and larynx	3
12	Kidney Renal Clear Cell Carcinoma	Kidney	277
13	Liver Hepatocellular Carcinoma	Liver	183
14	Lung Adenocarcinoma	Lung	53
15	Ovarian Serous Cystadenocarcinoma	Ovary	1071
16	Prostate Adenocarcinoma	Prostate	171
17	Sarcoma	Connective tissue	286
18	Skin Cutaneous Melanoma	Skin	2
19	Testicular Germ Cell Tumors	Testicles	258
20	Uveal Melanoma	Eye	106

**Table 2:** Generalized models’ architecture.

Model	Layer	Activation	Description
1A	Dense	ReLU	256
	Dense	ReLU	256
	Conv2D	filters = 20, kernel size = 8, strides = 1, padding = same	
	Dense	ReLU	256
	Dropout	0.5	
	Dense	Softmax	20
1B	Dense	ReLU	256
	BatchNormalization		
	Dropout		0.002
	Dense	ReLU	256
	Dense	ELU	128
	Dropout		0.05
	Dense	SELU	64
	Dense	ELU	64
	Dense	ELU	32
	Dense	ReLU	32
	Dense	Softmax	20

**Table 3:** Specialized models’ architecture.

Cancer Types	Model	Layer	Activation	Description
Male-specific cancer	2.A	Dense	Softmax	2
	2.B	Dense	Sigmoid	2
	2.C	Dense	Softplus	2
Brain cancer	3.A	Dense	Softmax	2
	3.B	Dense	Sigmoid	2
	3.C	Dense	Softplus	2
Excretory system cancer	4.A	Dense	Softmax	2
	4.B	Dense	Sigmoid	2
	4.C	Dense	Softplus	2
Digestive system cancer	5.A	Dense	ReLU	128
		Dense	Sigmoid	2
	5.B	Dense	ReLU	128
		Dense	Softmax	2
	5.C	Dense	ReLU	128
		Dense	Softplus	2
Female-specific cancer	6.A	Dense	ReLU	128
		Dense	Sigmoid	2
	6.B	Dense	ReLU	128
		Dense	Softmax	2
	6.C	Dense	ReLU	128
		Dense	Softplus	2

## Data Availability

The code for differentiating between cancer types based on copy number alterations (CNAs) is available on GitHub at https://github.com/michel-phylo/Adera3 (accessed on 21 July 2024).

## Supplementary Materials

The supporting information can be downloaded at: https://www.mdpi.com/article/10.3390/cimb46080490/s1. Figure S1. Accuracy, Loss, Specificity, Sensitivity, and AUC Values of the two generalized models. (A–D) CNN (E–H) SeLU. Figure S2. Classification of Male-specific cancers. (A–D) Softmax, (E–H) Sigmoid, and (I–L) Softplus. Figure S3. Classification of brain cancers. (A–D) Softmax, (E–H) Sigmoid, and (I–L) Softplus. Figure S4. Classification of excretory cancers. (A–D) Softmax, (E–H) Sigmoid, and (I–L) Softplus. Figure S5. Classification of digestive system cancers. (A–D) Softmax, (E–H) Sigmoid, and (I–L) Softplus. Figure S6. Classification of digestive system cancers. (A–D) Softmax, (E–H) Sigmoid, and (I–L) Softplus. Figure S7. Biological explainability analysis of the generalized CNN model performance.
