# Supplementary material for: Using Copy Number Variation Data and Neural Networks to Predict Cancer Metastasis Origin Achieves High Area under the Curve Value with a Trade-Off in Precision
Source: Curr Issues Mol Biol. 2024 Aug 1;46(8):8301–19. doi: 10.3390/cimb46080490 (PMC11352492; doi:10.3390/cimb46080490)

Figure S1. Accuracy, Loss, Specificity, Sensitivity, and AUC Values of the two generalized models. (A-D) CNN (E-H) SeLU.

1A

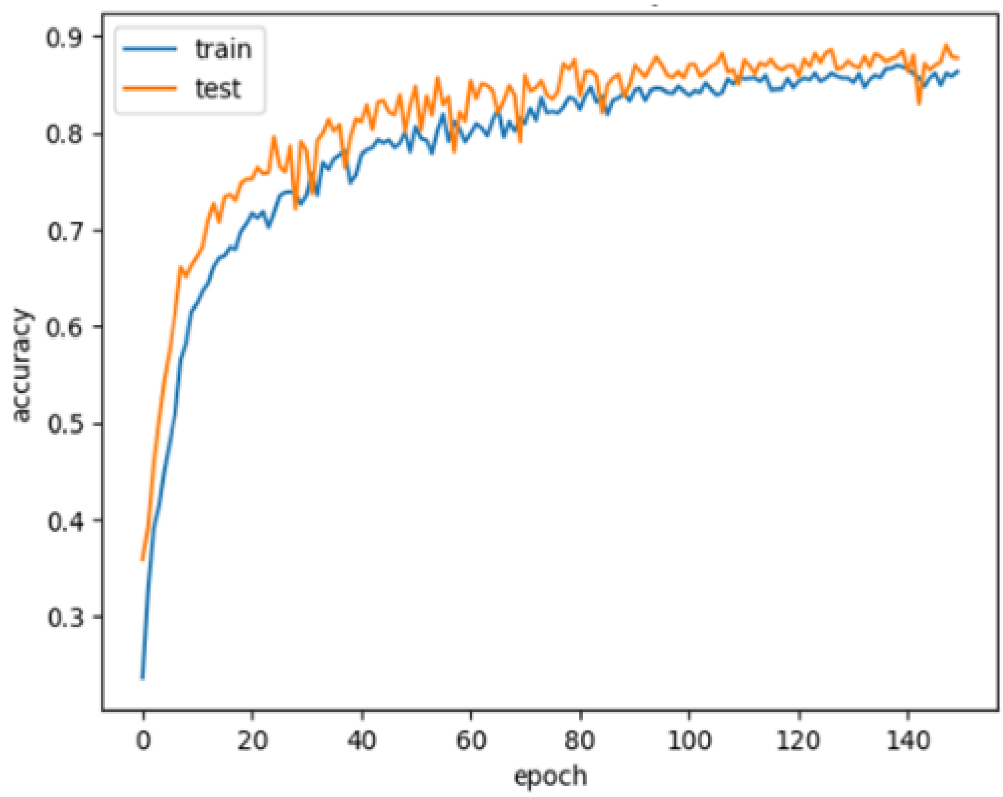

1B

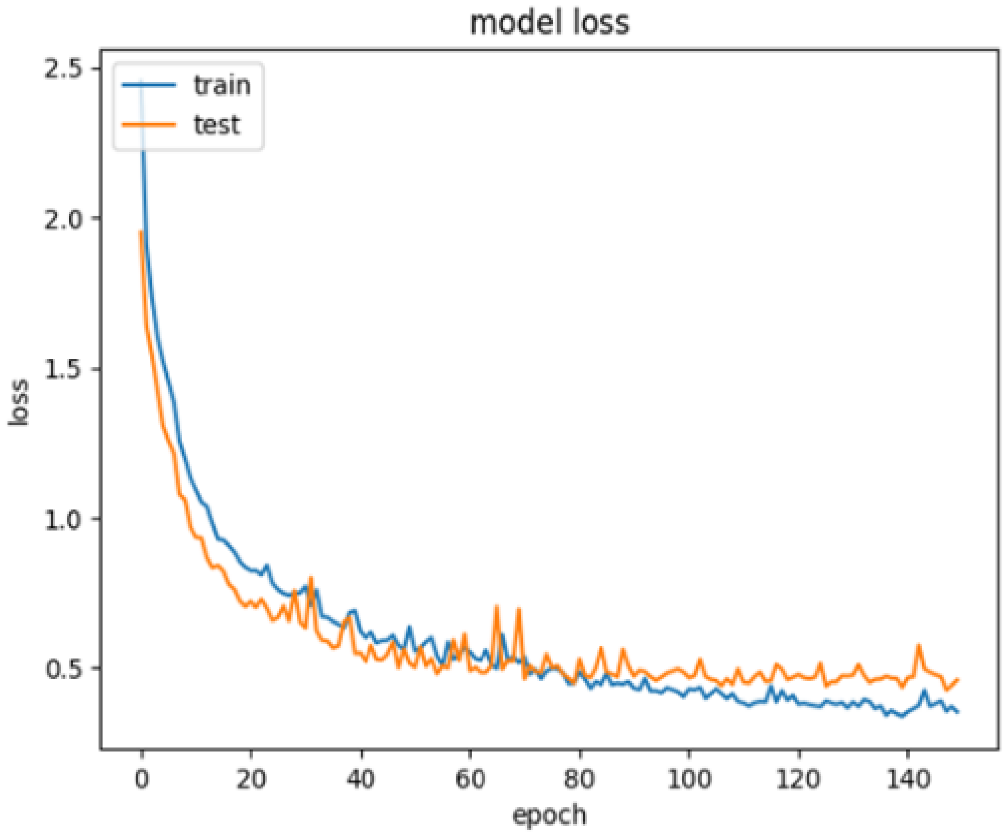

1C

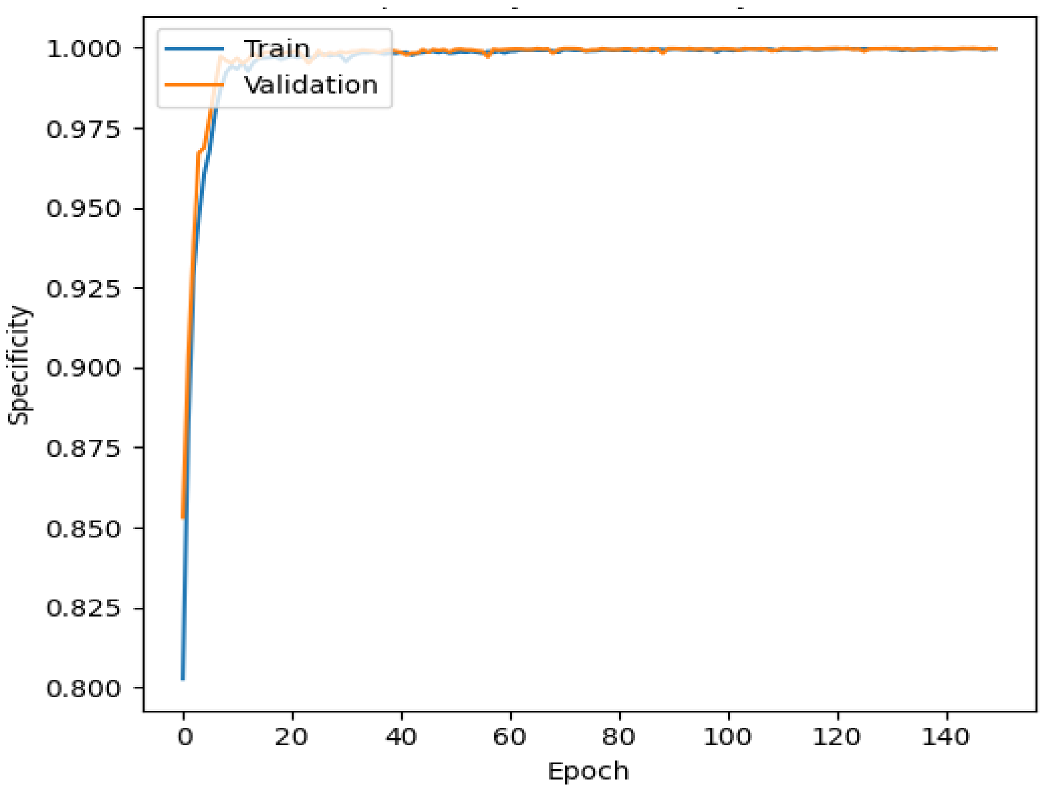
Train
Validation
Specificity
Epoch
1.000
0.975
0.950
0.925
0.900
0.875
0.850
0.825
0.800
0
20
40
60
80
100
120
140

1D

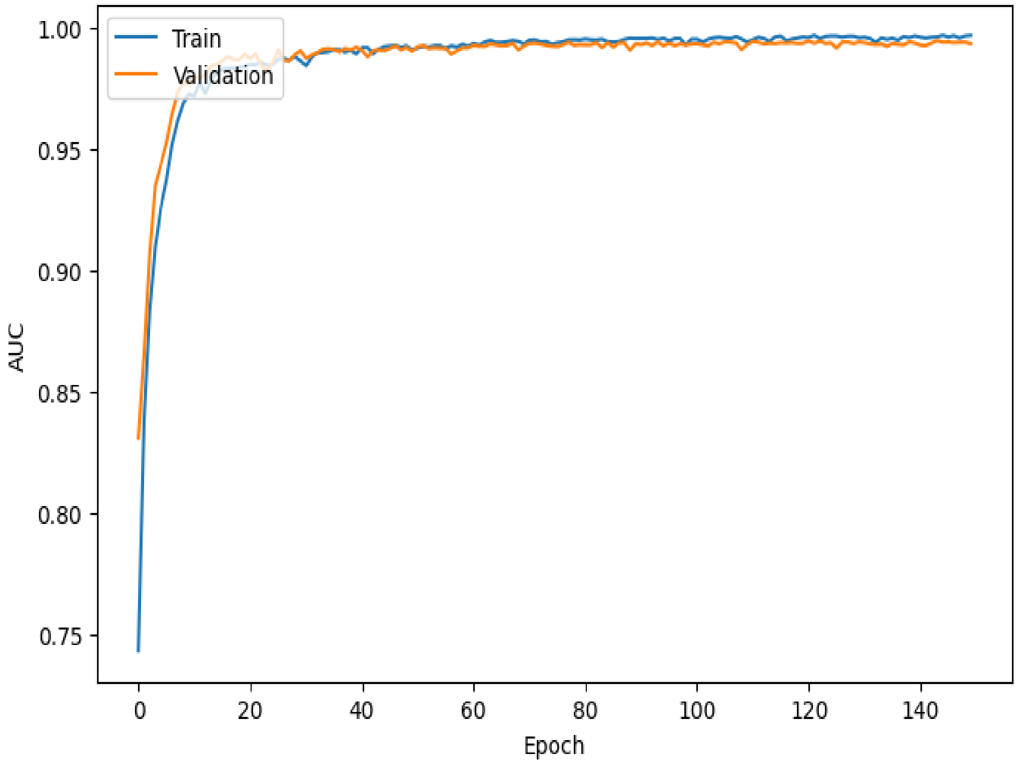
Train
Validation
AUC
Epoch
1.00
0.95
0.90
0.85
0.80
0.75
0
20
40
60
80
100
120
140

1E

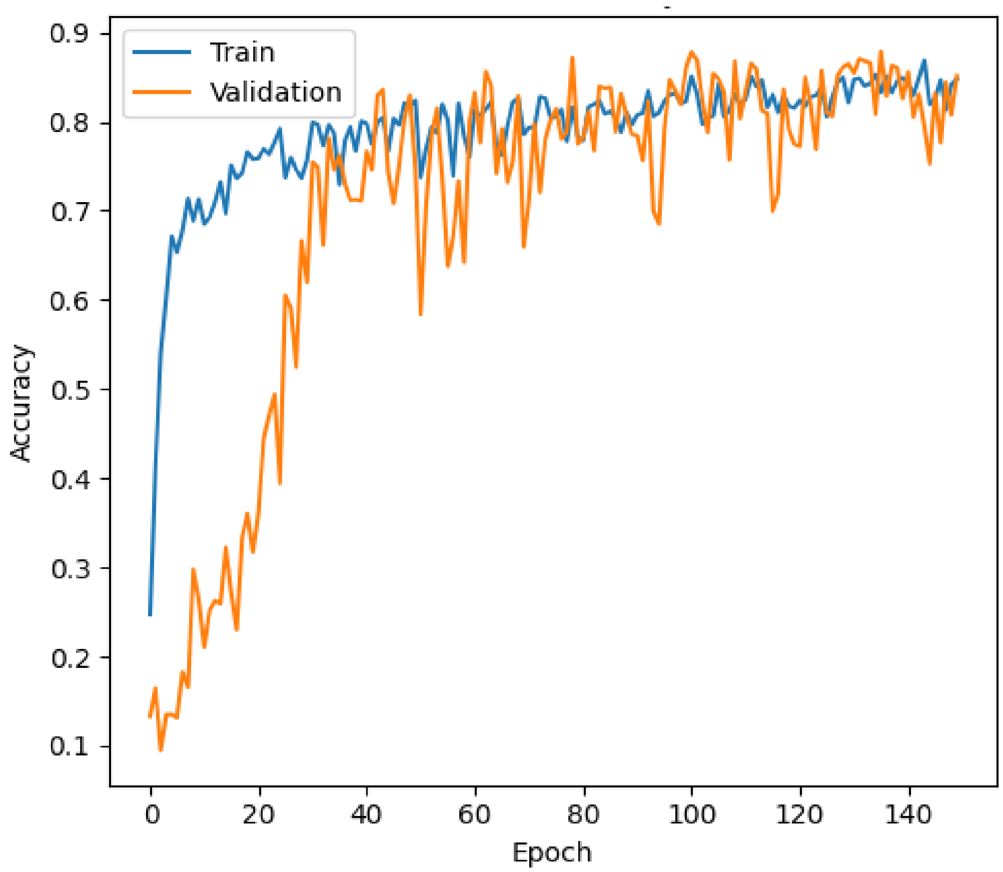
Train
Validation
Accuracy
0.9
0.8
0.7
0.6
0.5
0.4
0.3
0.2
0.1
0
20
40
60
80
100
120
140
Epoch

1F

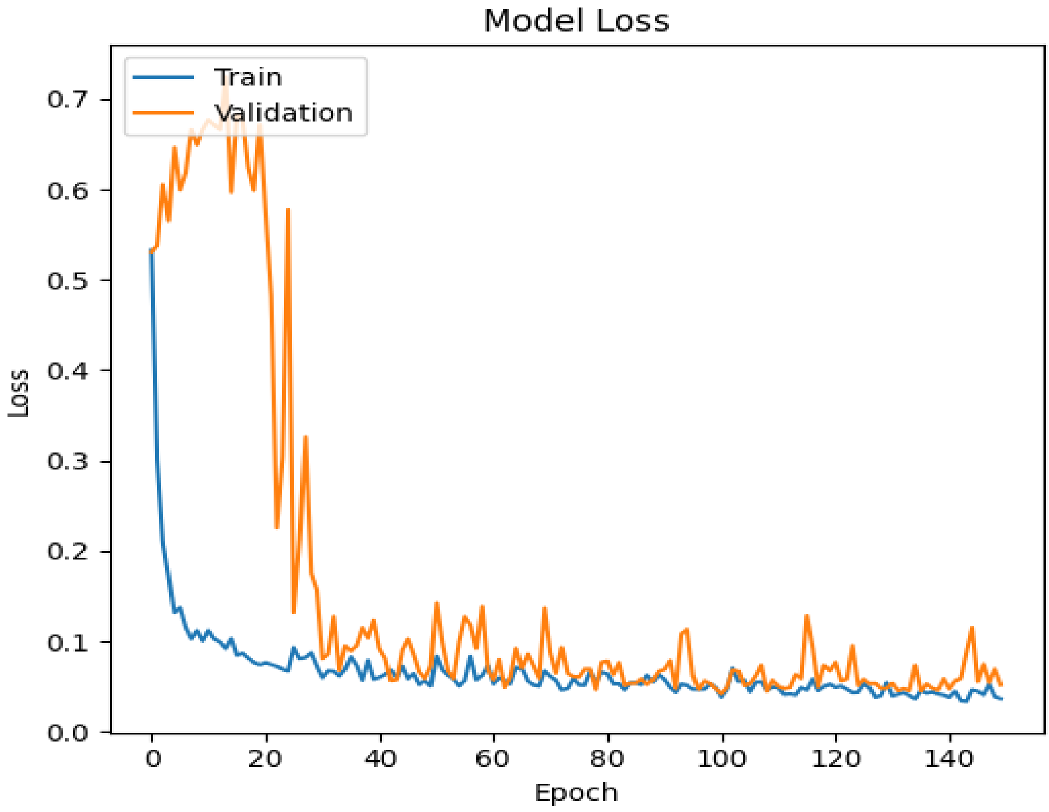
Model Loss
Train
Validation
Loss
0.7
0.6
0.5
0.4
0.3
0.2
0.1
0.0
0
20
40
60
80
100
120
140
Epoch

1G

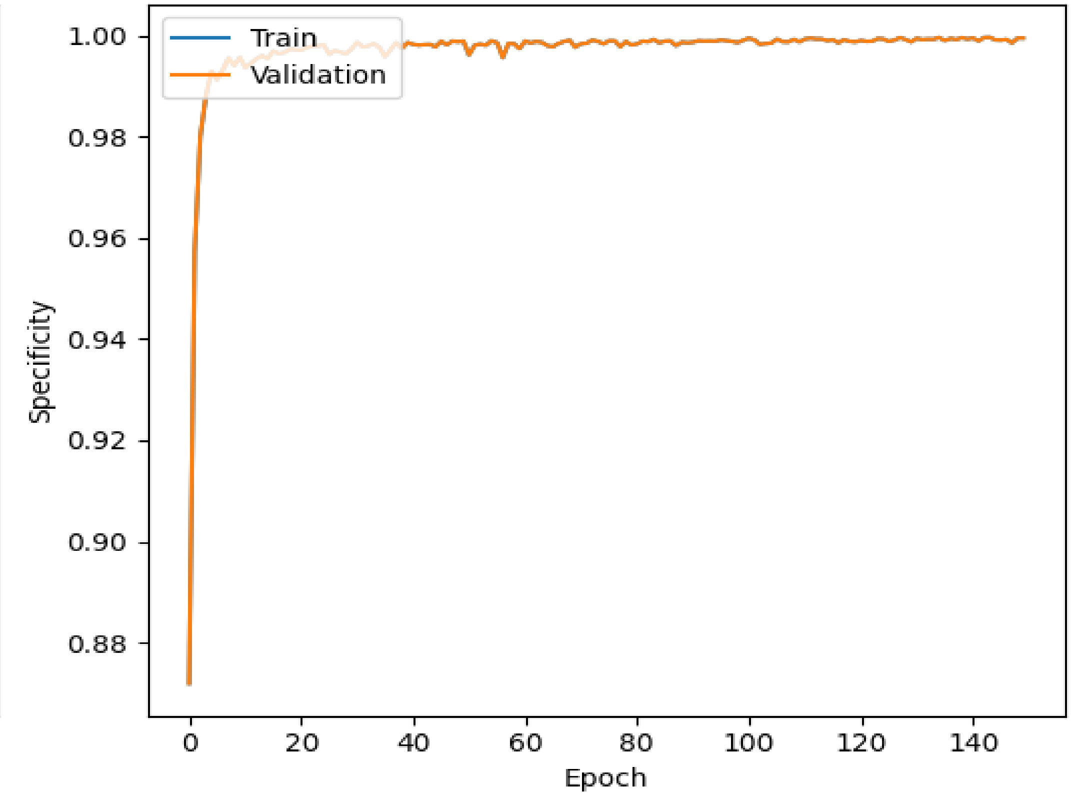

1H

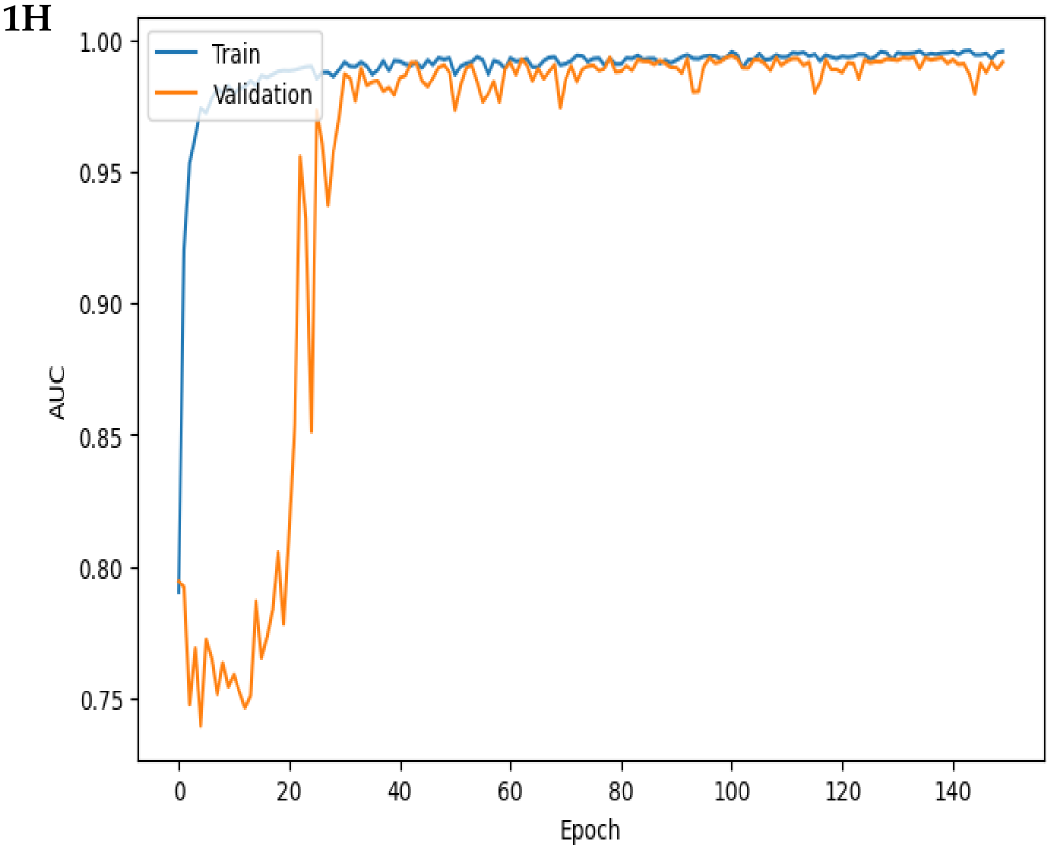

Figure S2. Classification of Male-specific cancers. (A-D) Softmax,(E-H) Sigmoid, and (I-L) Softplus.

2A

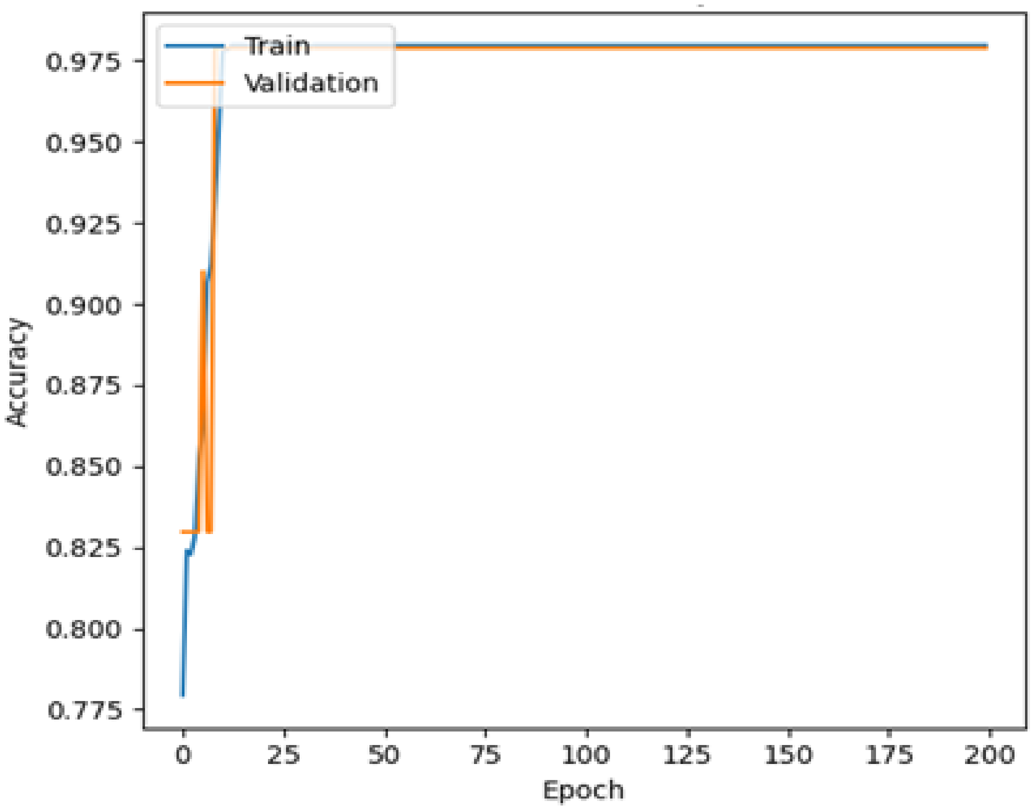
Train
Validation
Accuracy
0.975
0.950
0.925
0.900
0.875
0.850
0.825
0.800
0.775
0
25
50
75
100
125
150
175
200
Epoch

2B

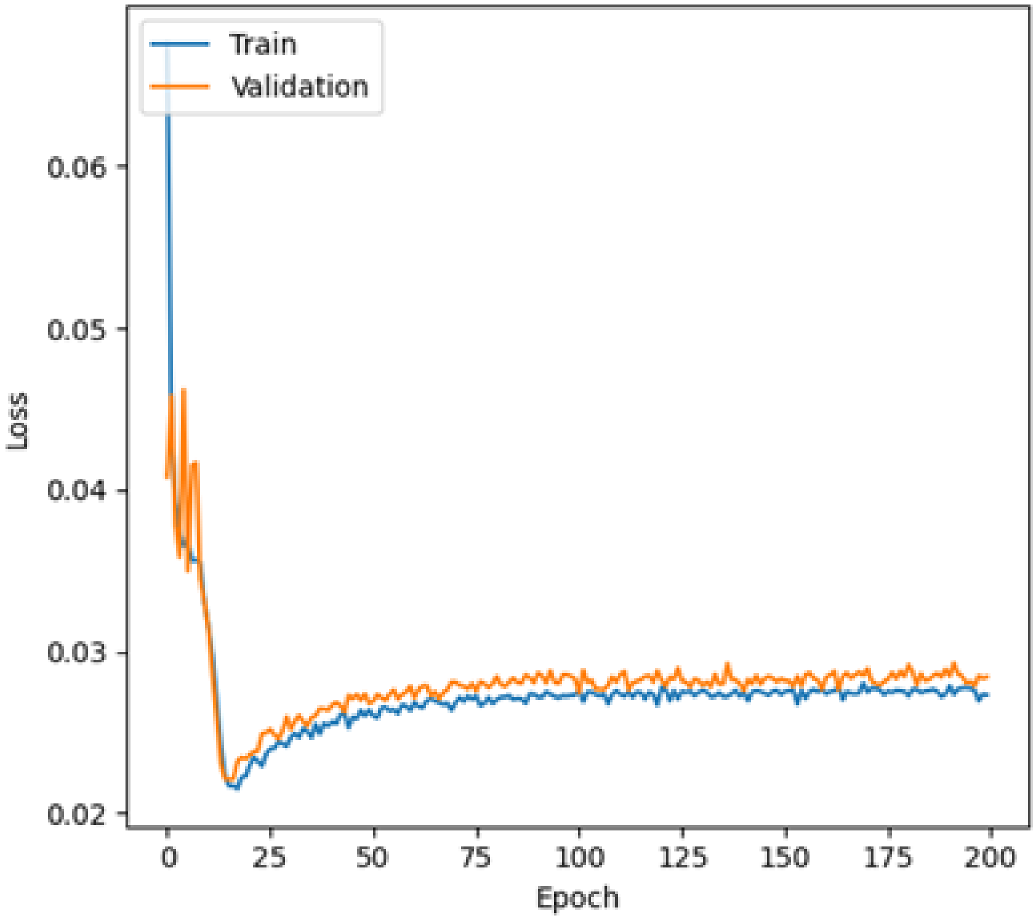
Train
Validation
Loss
0.06
0.05
0.04
0.03
0.02
0
25
50
75
100
125
150
175
200
Epoch

2C

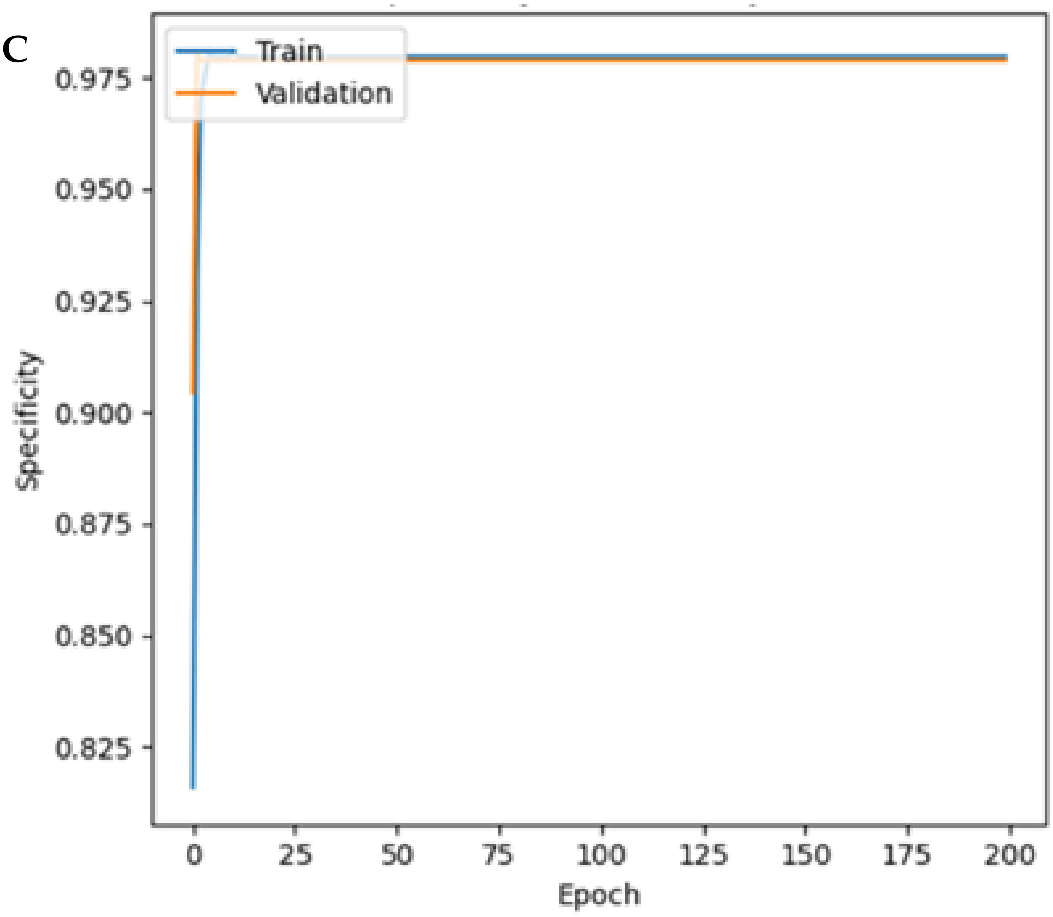
Train
Validation
Specificity
0.975
0.950
0.925
0.900
0.875
0.850
0.825
0
25
50
75
100
125
150
175
200
Epoch

2D

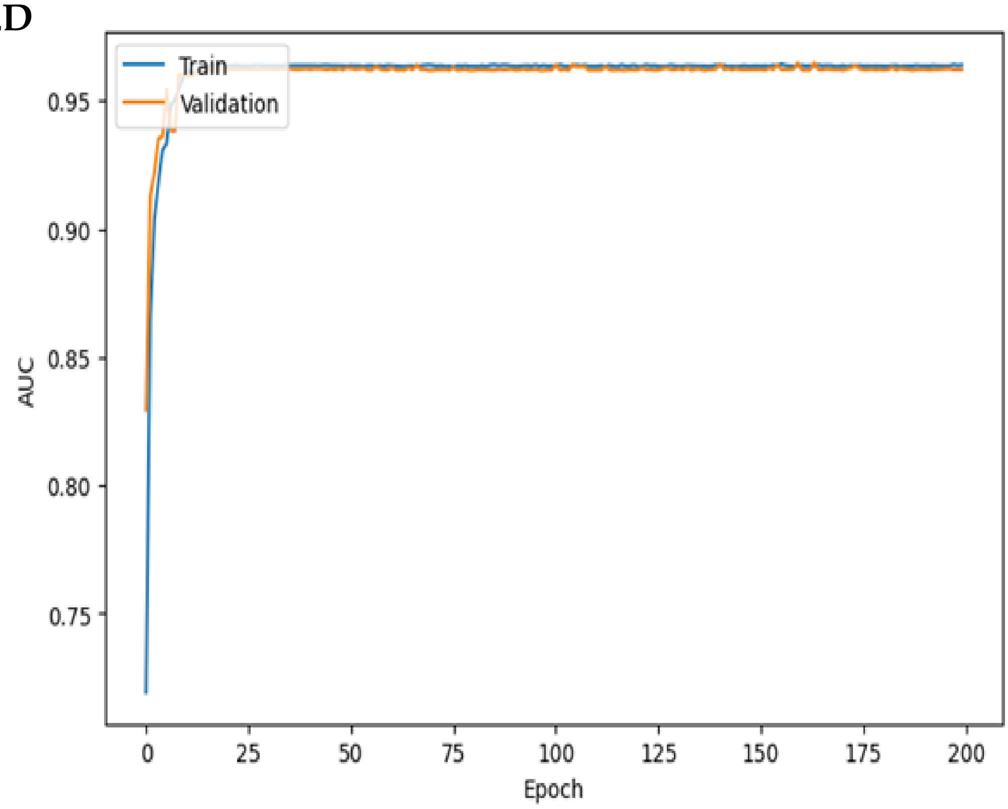
Train
Validation
AUC
0.95
0.90
0.85
0.80
0.75
0
25
50
75
100
125
150
175
200
Epoch

2E

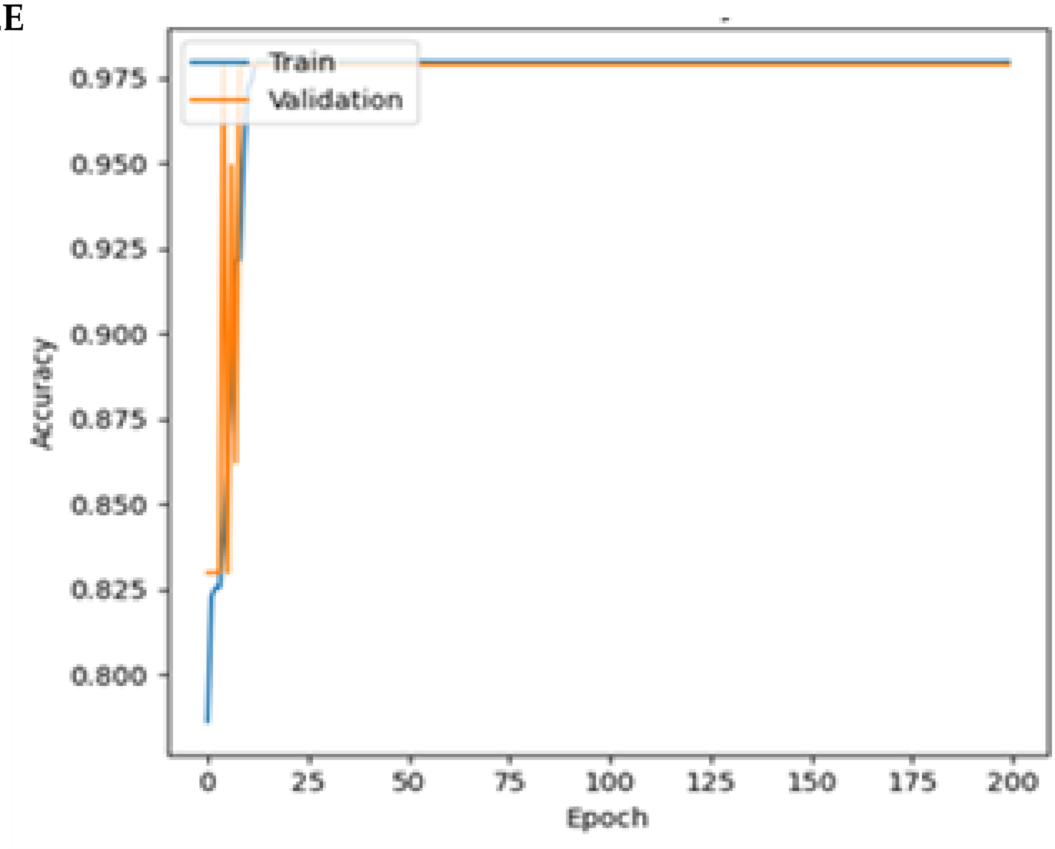
Train
Validation
0.975
0.950
0.925
0.900
0.875
0.850
0.825
0.800
Accuracy
0
25
50
75
100
125
150
175
200
Epoch

2F

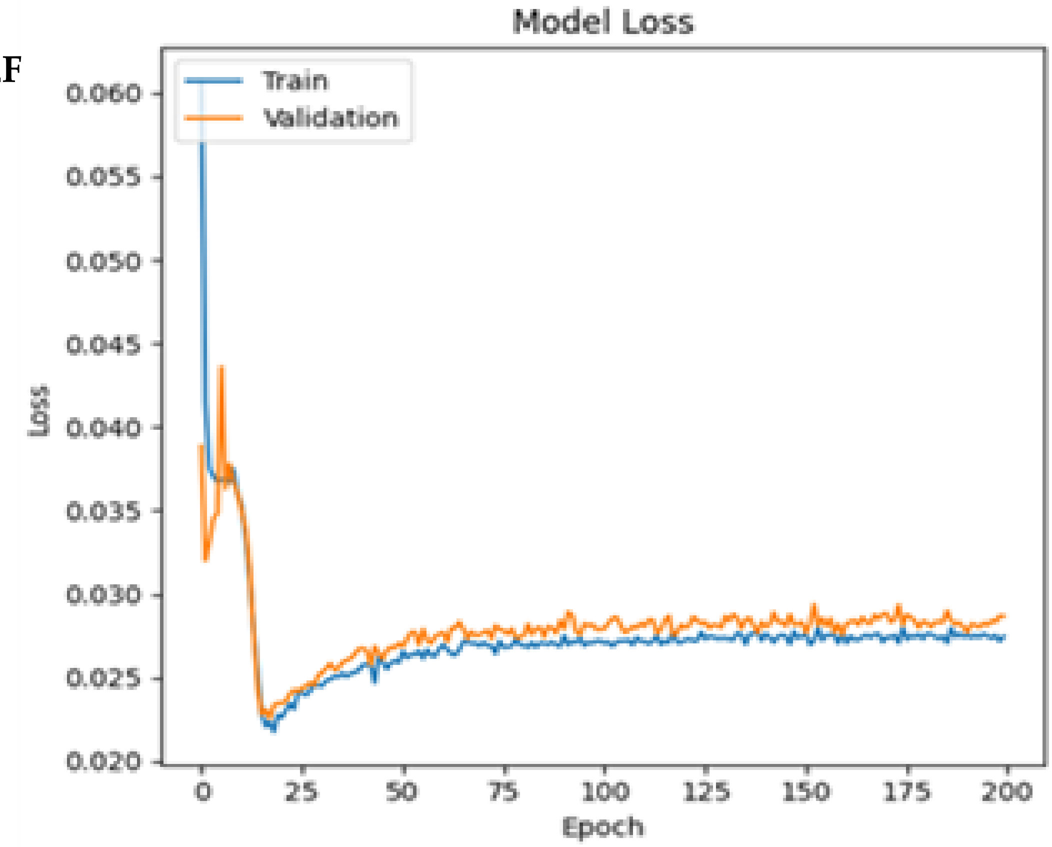
Model Loss
Train
Validation
0.060
0.055
0.050
0.045
0.040
0.035
0.030
0.025
0.020
Loss
0
25
50
75
100
125
150
175
200
Epoch

2G

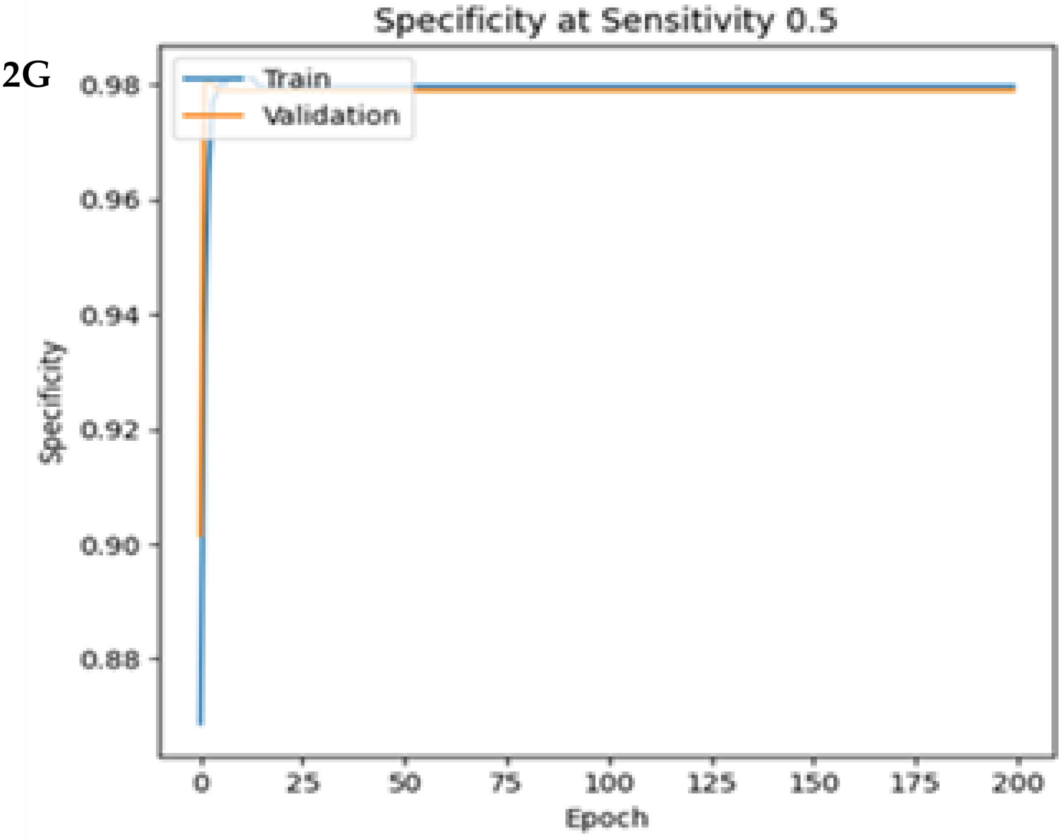
Specificity at Sensitivity 0.5
Train
Validation
Specificity
0.98
0.96
0.94
0.92
0.90
0.88
0
25
50
75
100
125
150
175
200
Epoch

2H

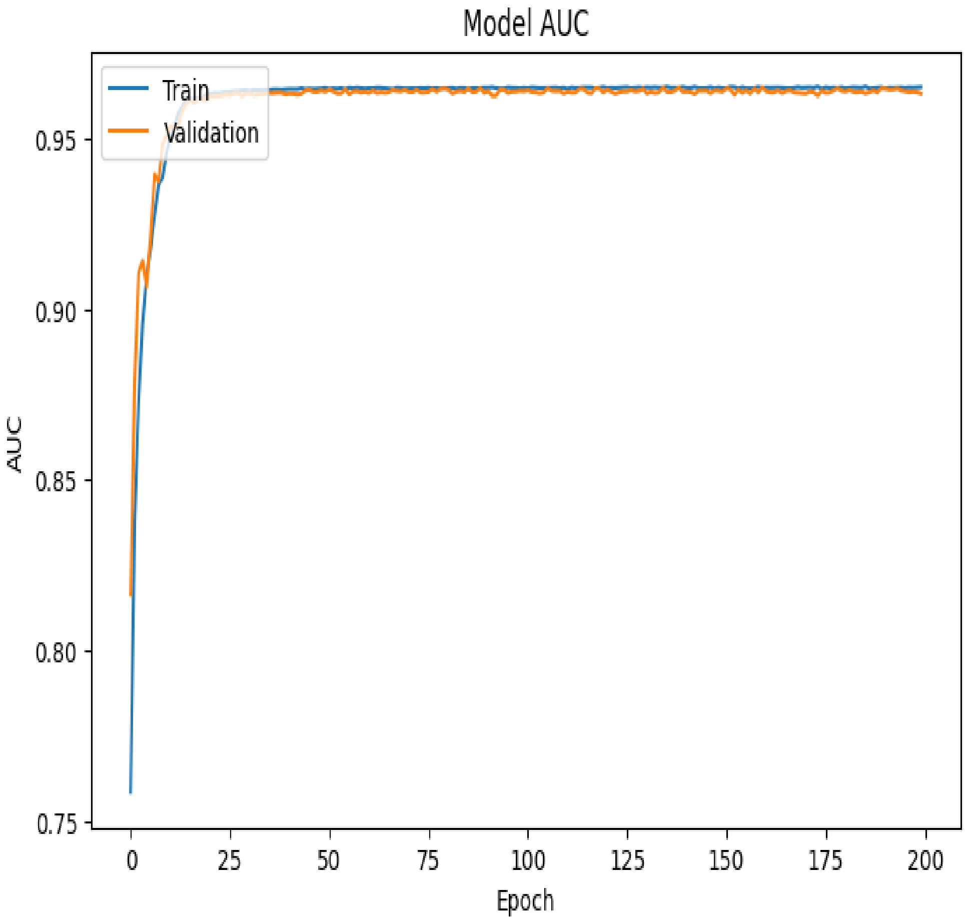
Model AUC
Train
Validation
AUC
0.95
0.90
0.85
0.80
0.75
0
25
50
75
100
125
150
175
200
Epoch

2I

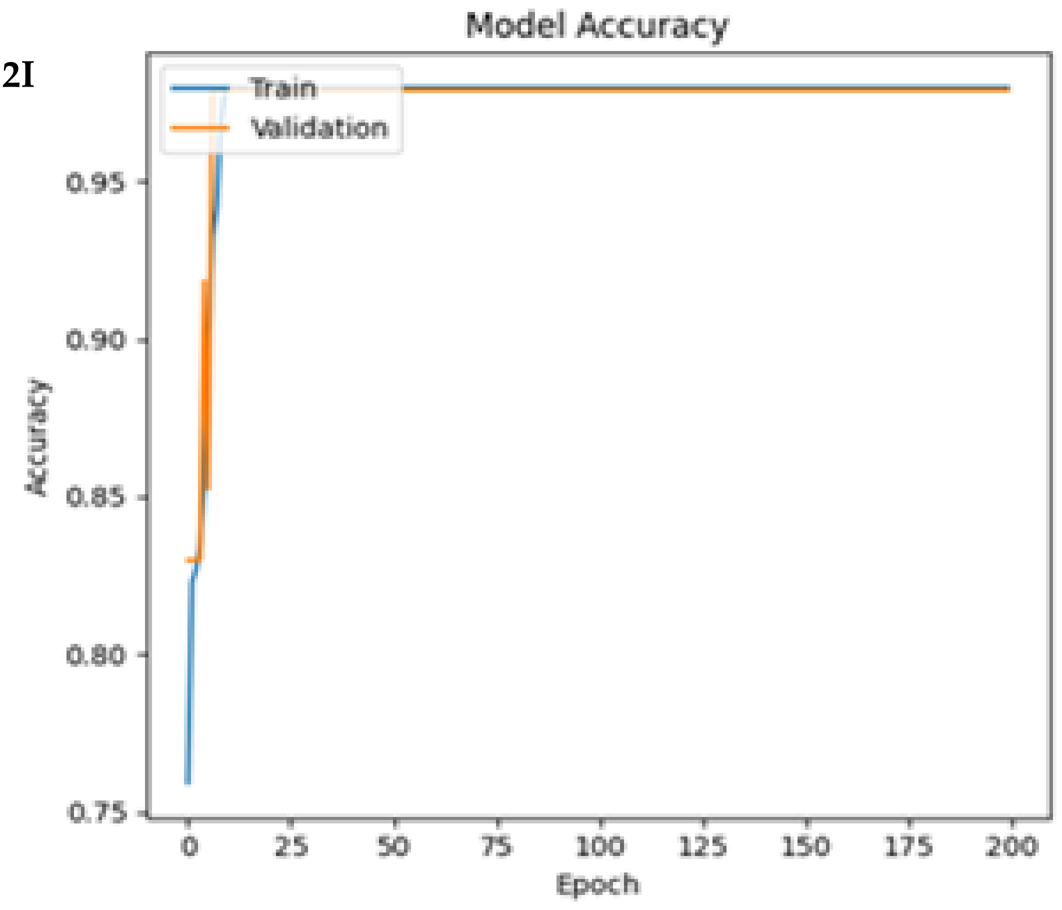
Model Accuracy
Train
Validation
0.95
0.90
0.85
0.80
0.75
Accuracy
0
25
50
75
100
125
150
175
200
Epoch

2J

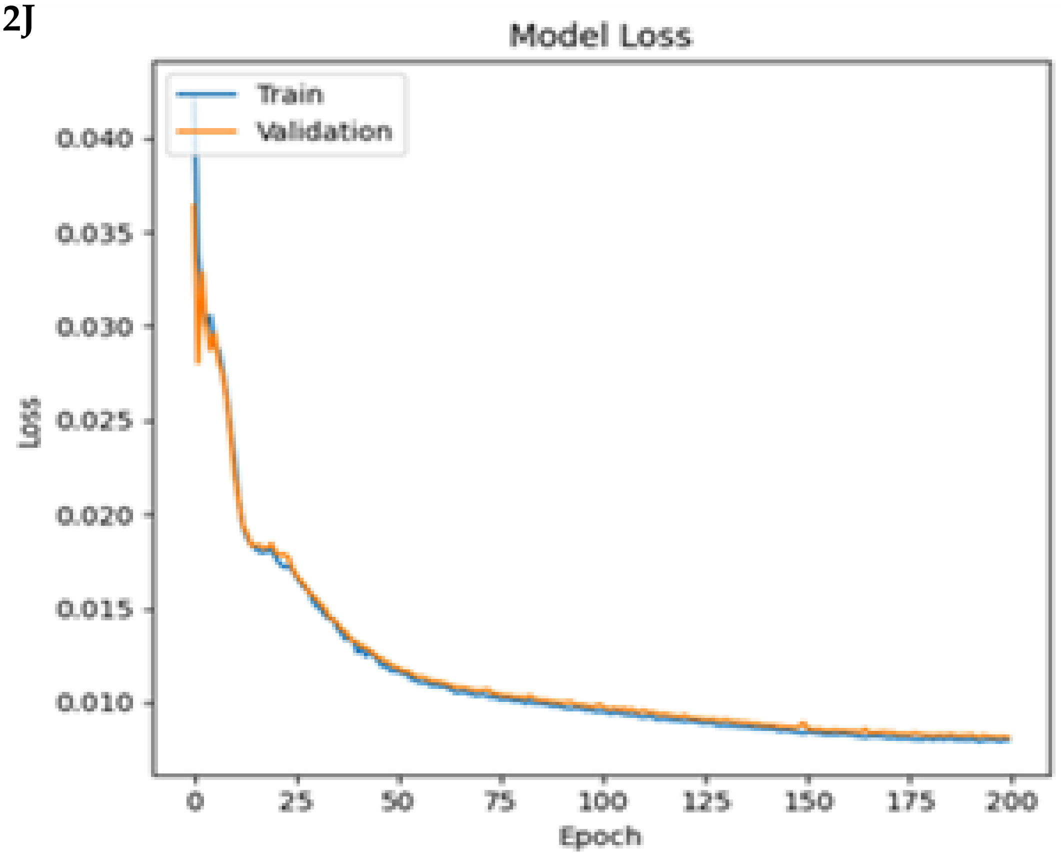
Model Loss
Train
Validation
0.040
0.035
0.030
0.025
0.020
0.015
0.010
Loss
0
25
50
75
100
125
150
175
200
Epoch

2K

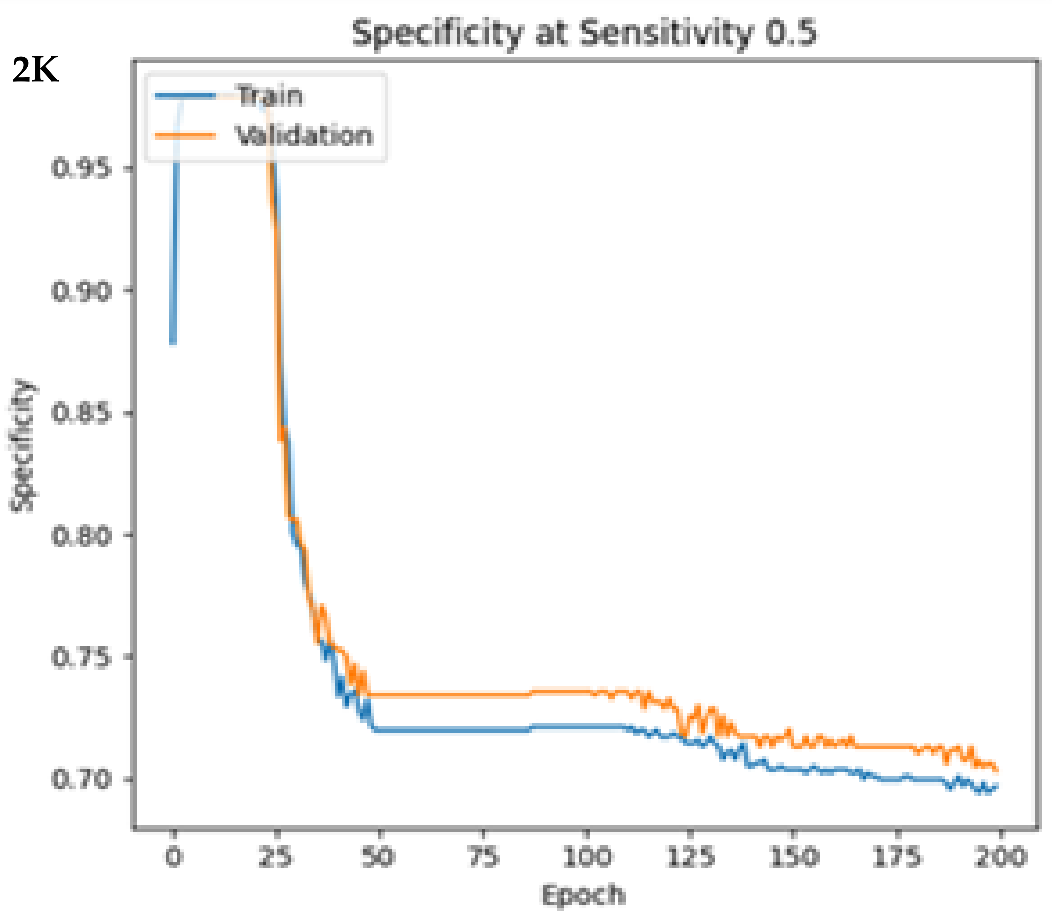
Specificity at Sensitivity 0.5
Train
Validation
Specificity
0.95
0.90
0.85
0.80
0.75
0.70
0
25
50
75
100
125
150
175
200
Epoch

2L

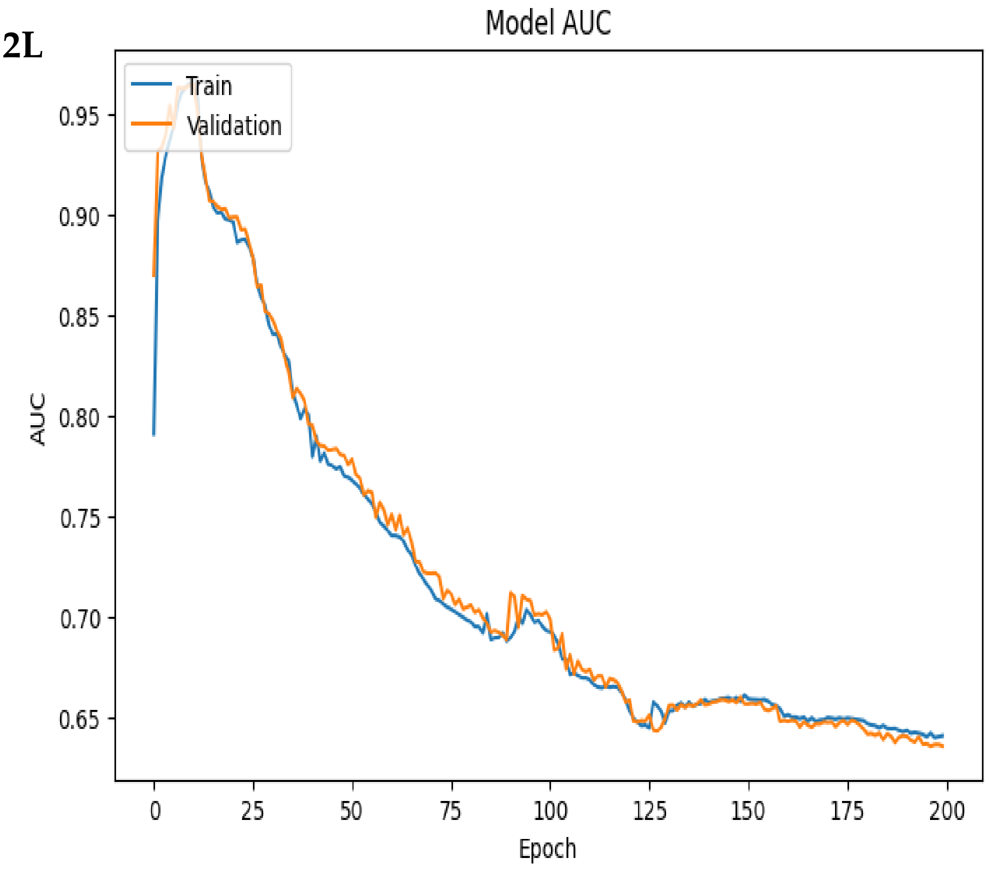
Model AUC
Train
Validation
AUC
0.95
0.90
0.85
0.80
0.75
0.70
0.65
0
25
50
75
100
125
150
175
200
Epoch

Figure S3. Classification of brain cancers. (A-D) Softmax,(E-H) Sigmoid, and (I-L) Softplus.

3A

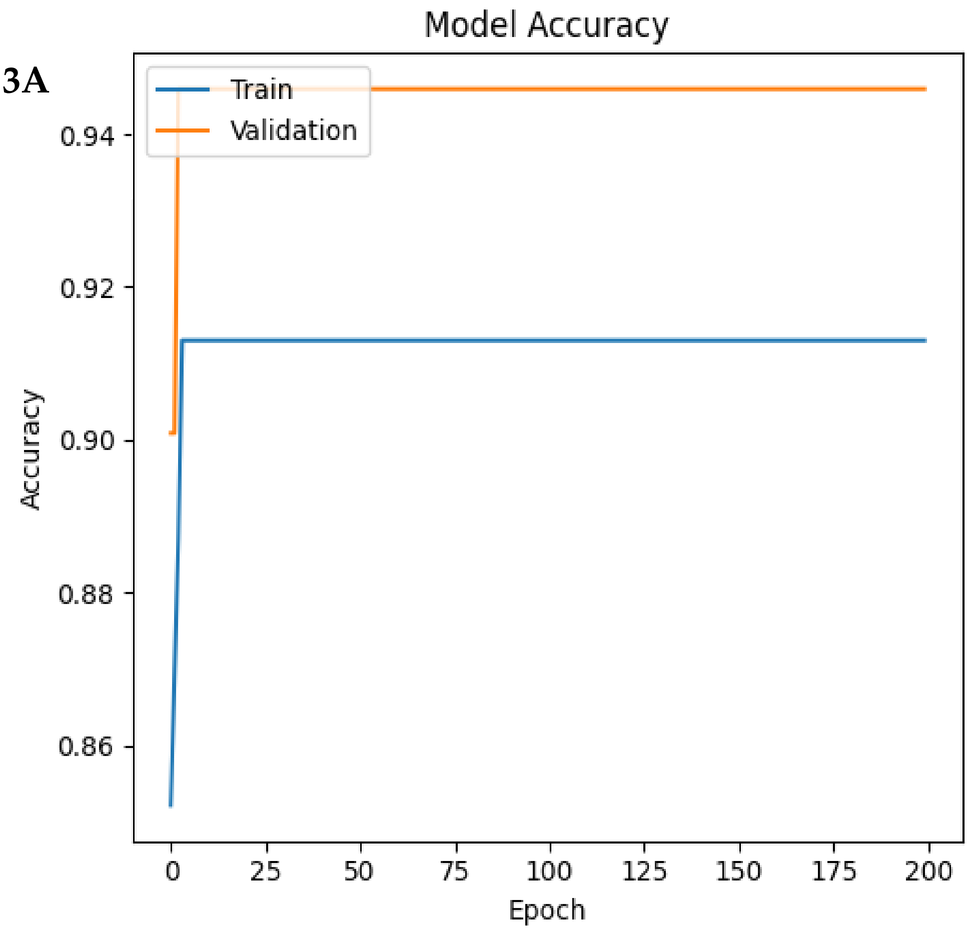

3B

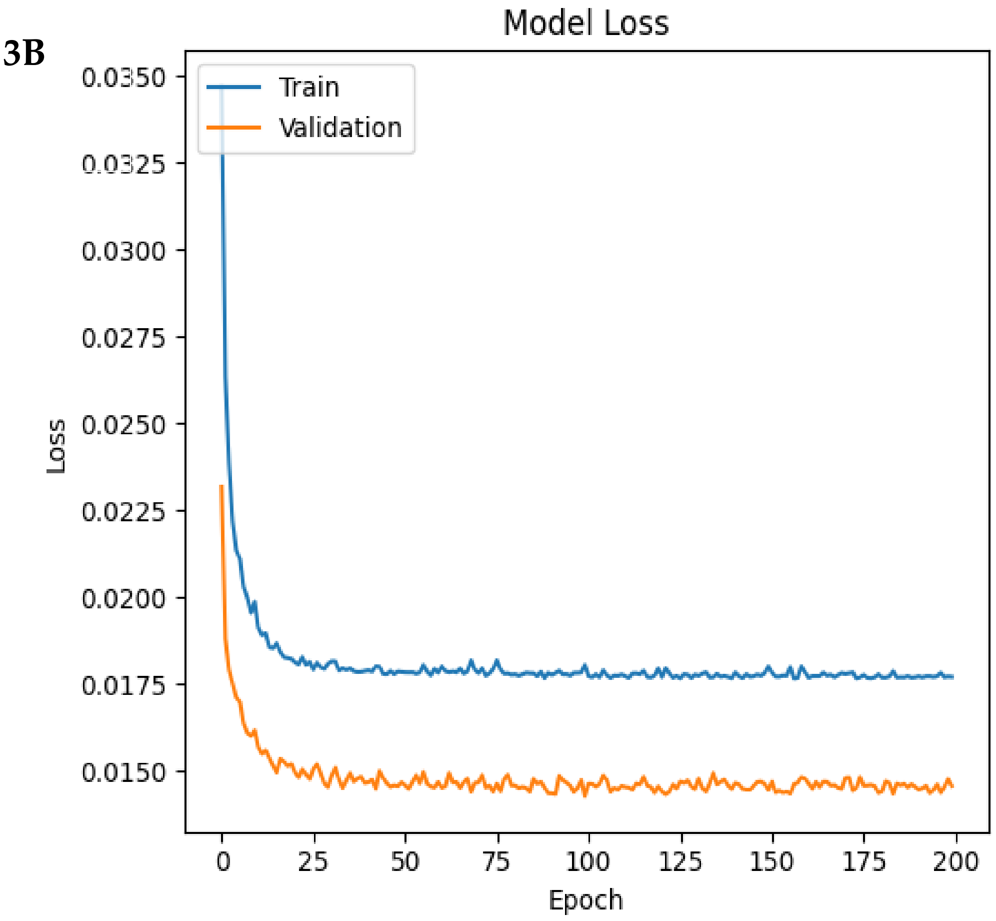

3C

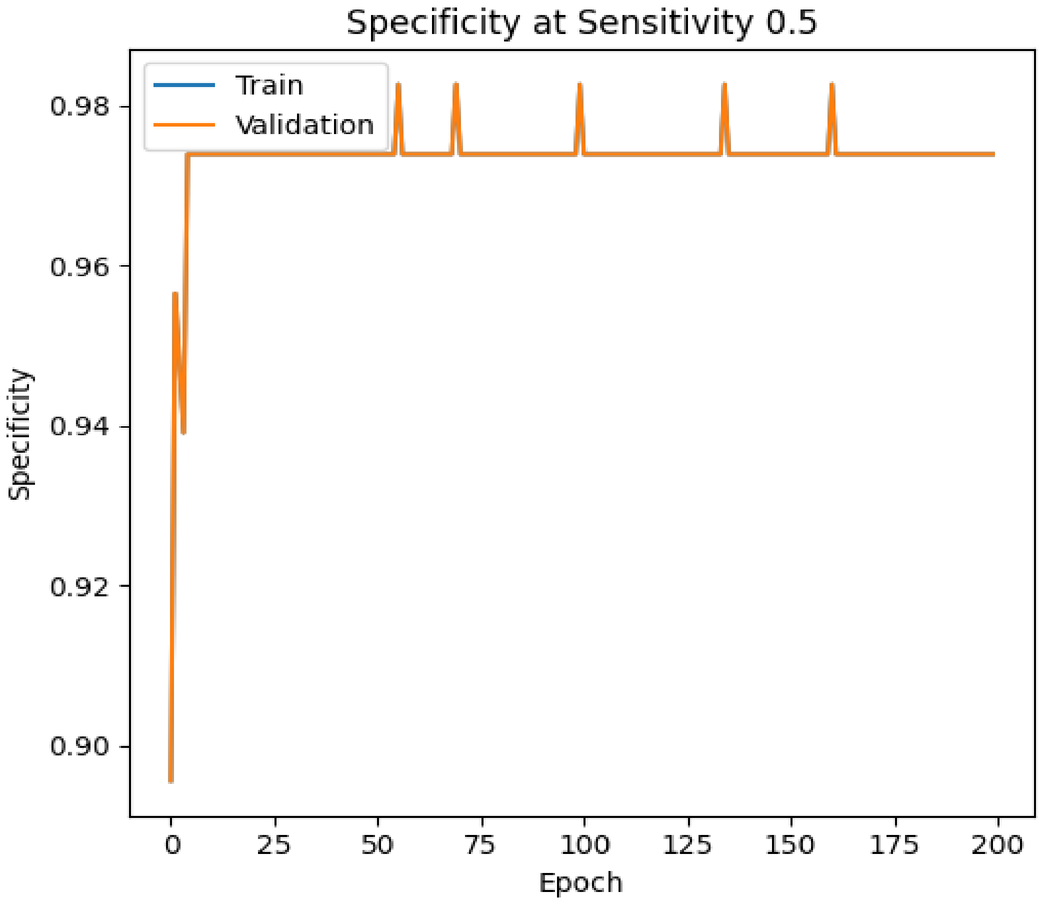
Specificity at Sensitivity 0.5
Train
Validation
0.98
0.96
0.94
0.92
0.90
Specificity
0
25
50
75
100
125
150
175
200
Epoch

3D

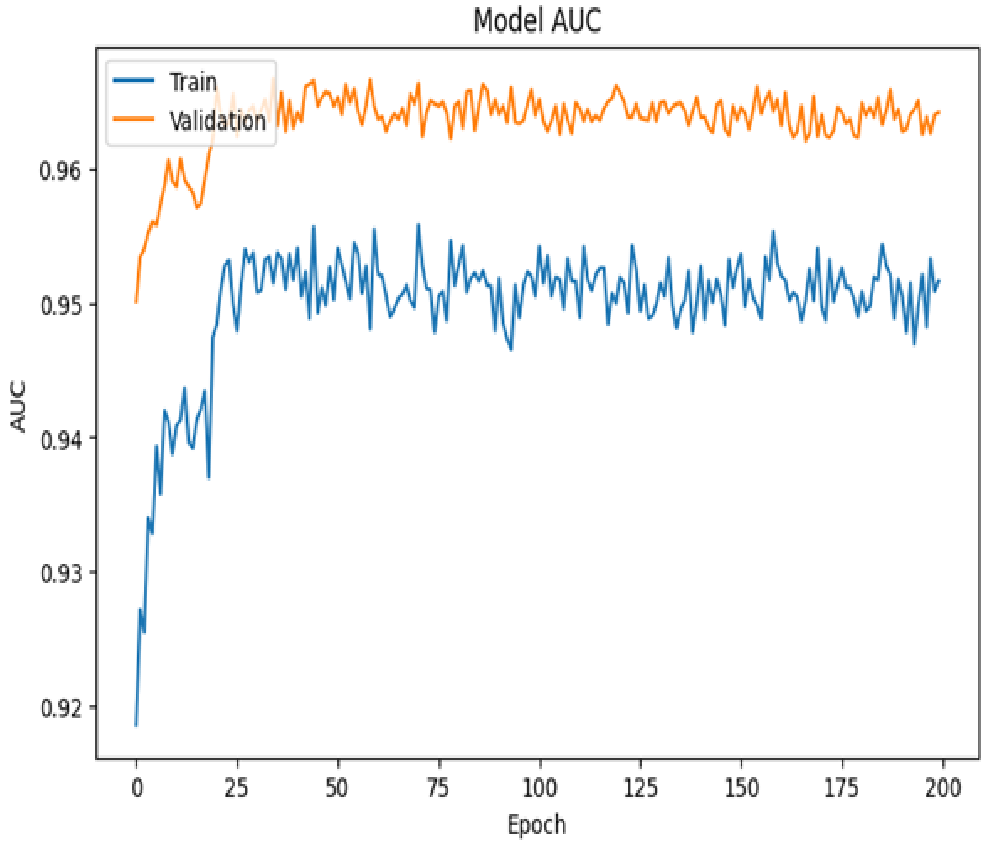
Model AUC
Train
Validation
0.96
0.95
0.94
0.93
0.92
AUC
0
25
50
75
100
125
150
175
200
Epoch

3E

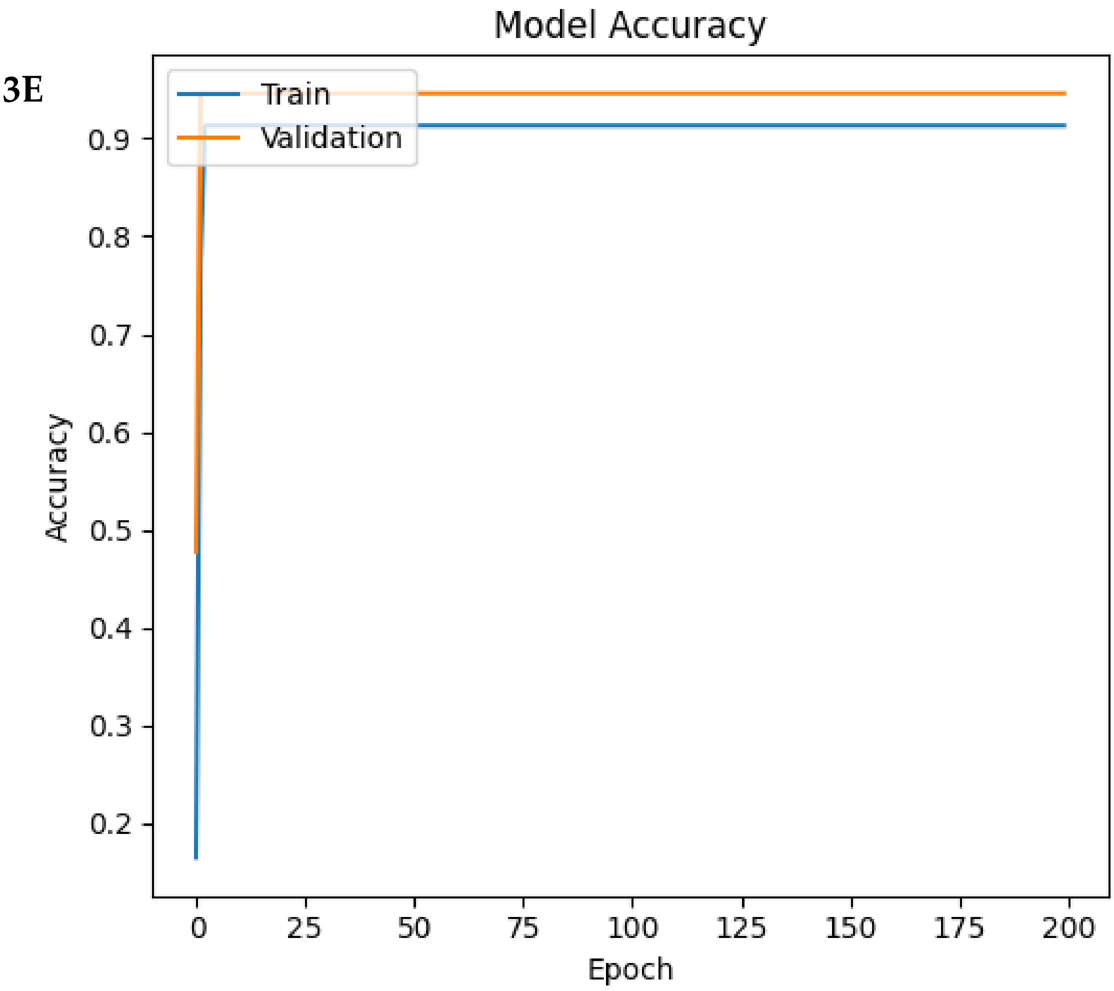
Model Accuracy
Train
Validation
Accuracy
0.9
0.8
0.7
0.6
0.5
0.4
0.3
0.2
0
25
50
75
100
125
150
175
200
Epoch

3F

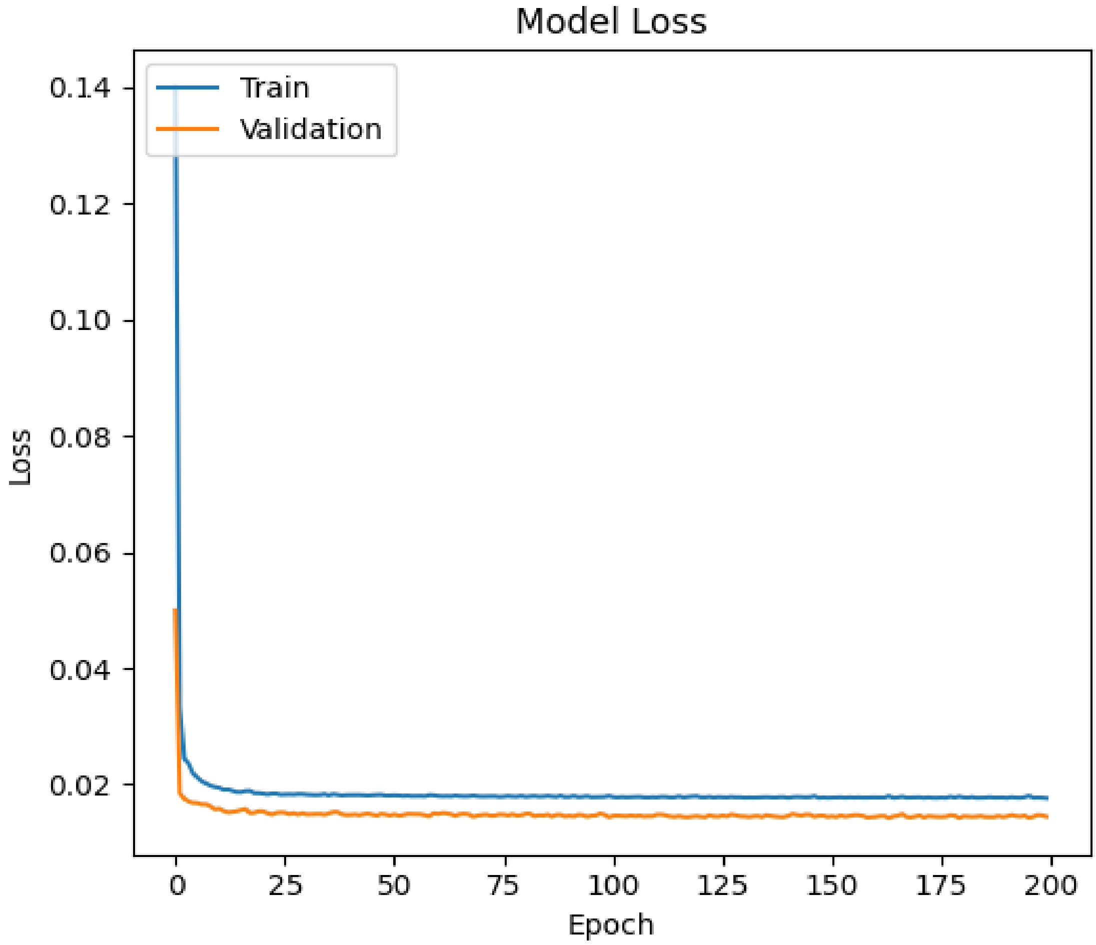
Model Loss
Train
Validation
Loss
0.14
0.12
0.10
0.08
0.06
0.04
0.02
0
25
50
75
100
125
150
175
200
Epoch

3G

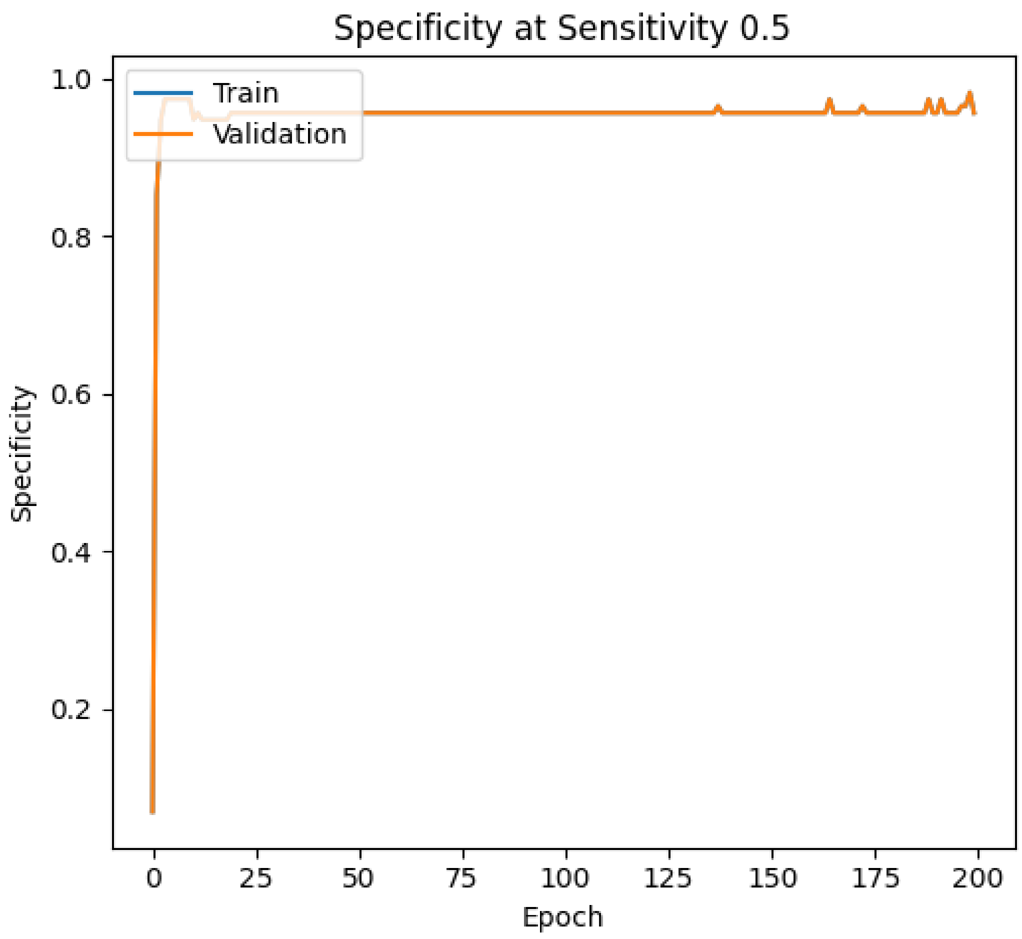
Specificity at Sensitivity 0.5
Train
Validation
Specificity
1.0
0.8
0.6
0.4
0.2
0
25
50
75
100
125
150
175
200
Epoch

3H

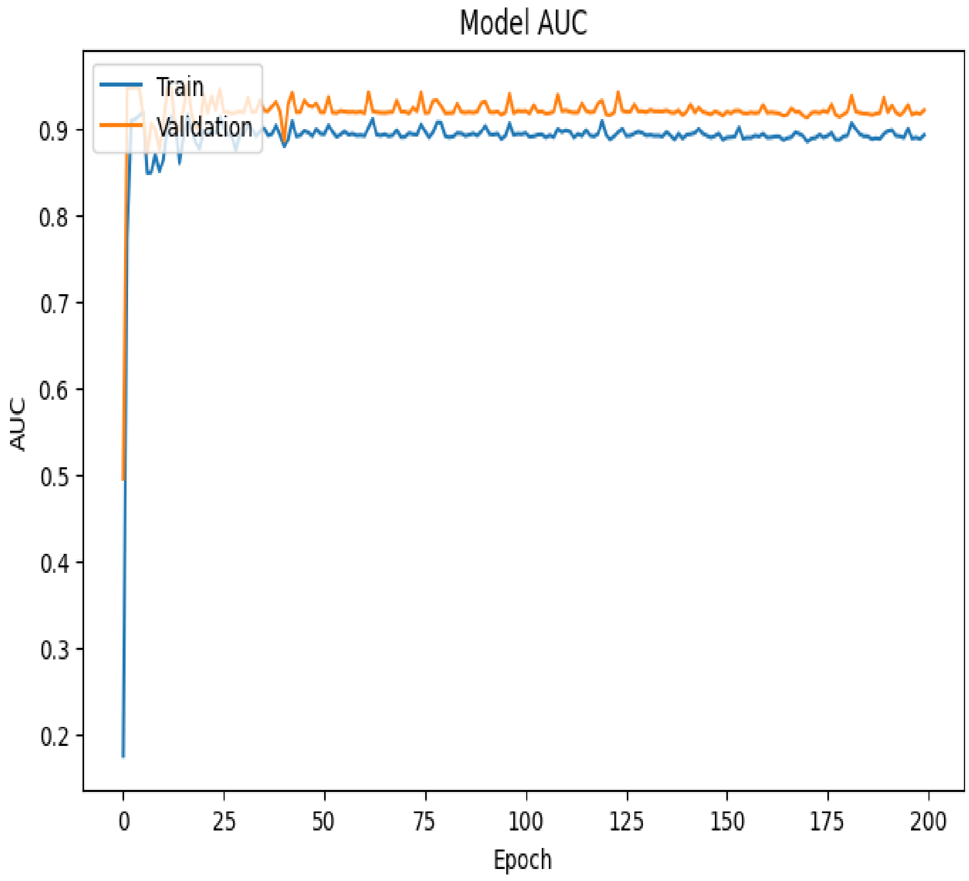
Model AUC
Train
Validation
AUC
0.9
0.8
0.7
0.6
0.5
0.4
0.3
0.2
0
25
50
75
100
125
150
175
200
Epoch

3I

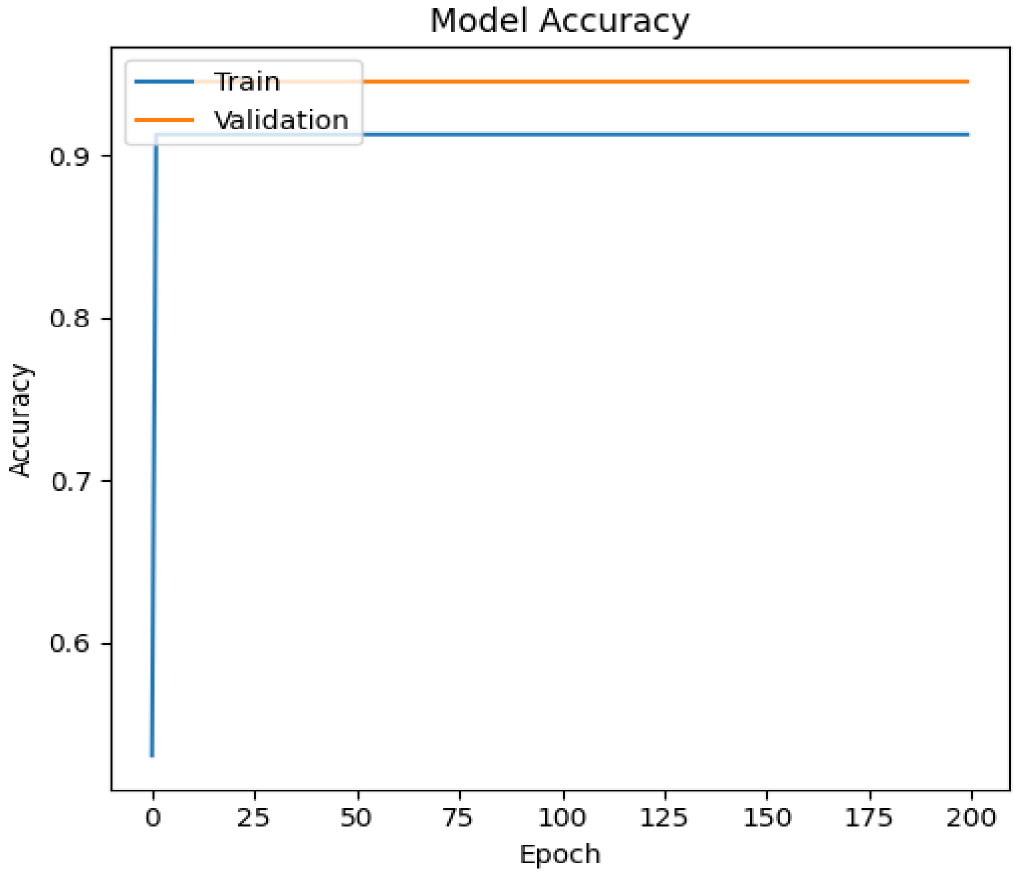
Model Accuracy
Train
Validation
Accuracy
0.9
0.8
0.7
0.6
0
25
50
75
100
125
150
175
200
Epoch

3J

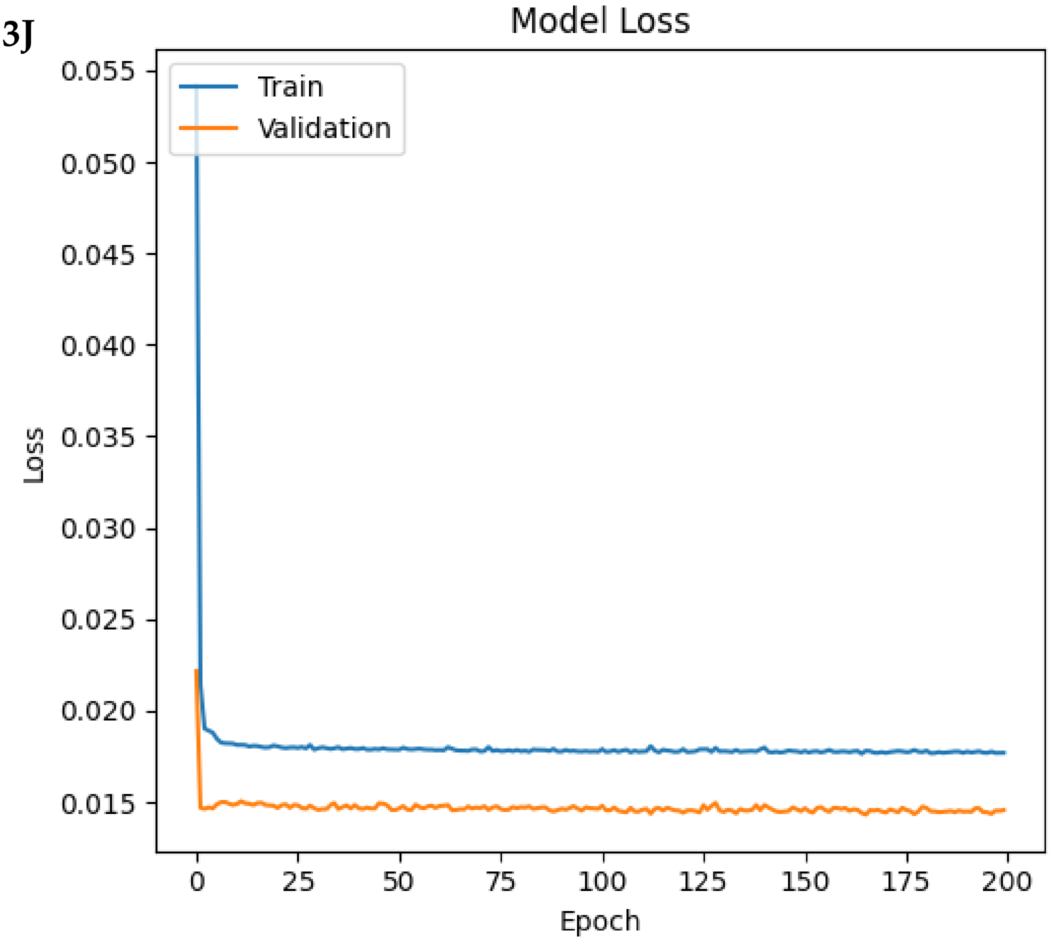
Model Loss
Train
Validation
Loss
0.055
0.050
0.045
0.040
0.035
0.030
0.025
0.020
0.015
0
25
50
75
100
125
150
175
200
Epoch

3K

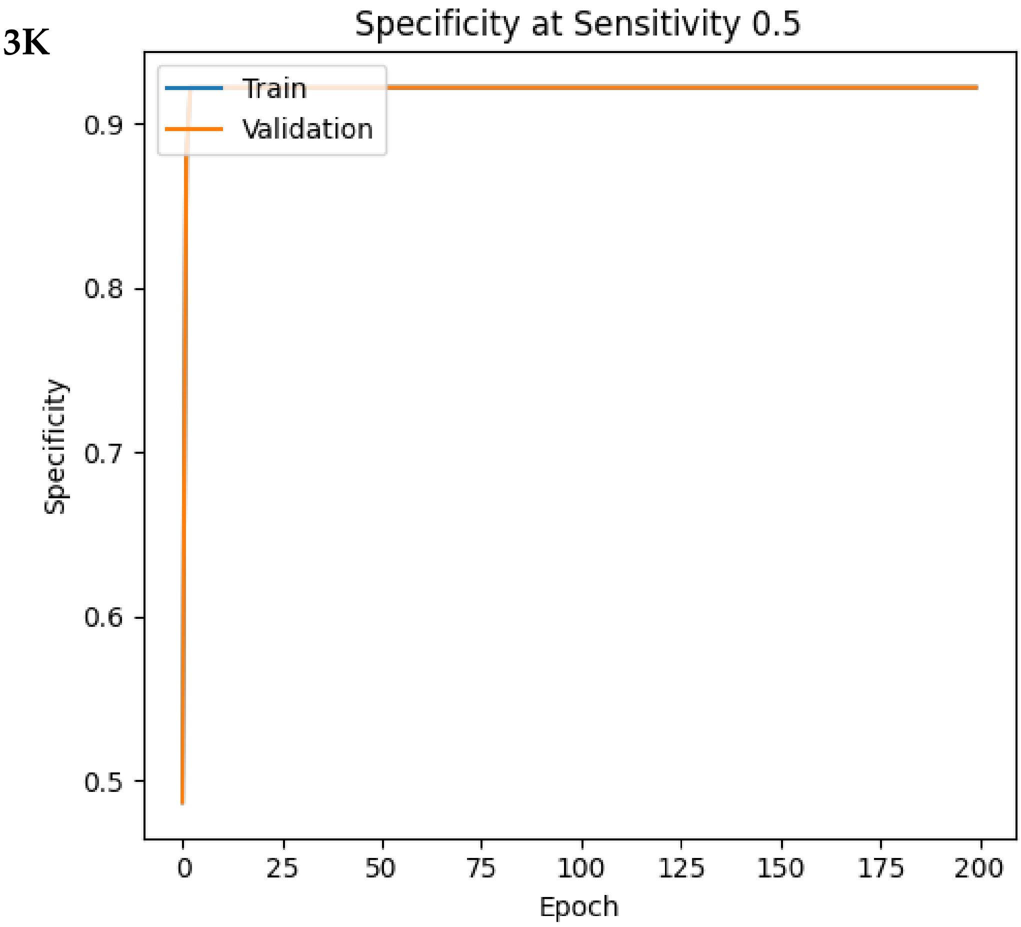
Specificity at Sensitivity 0.5
Train
Validation
0.9
0.8
0.7
0.6
0.5
Specificity
0
25
50
75
100
125
150
175
200
Epoch

3L

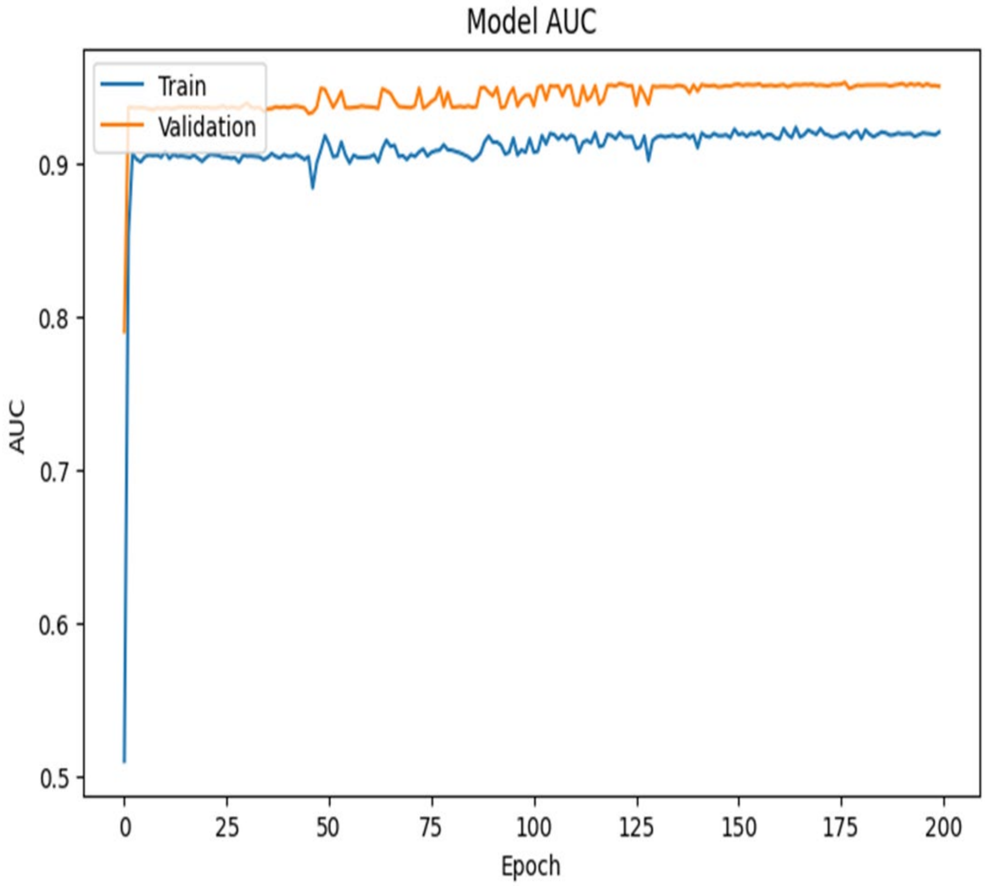
Model AUC
Train
Validation
0.9
0.8
0.7
0.6
0.5
AUC
0
25
50
75
100
125
150
175
200
Epoch

current issues in
molecular biology

Figure S4. Classification of excretory cancers. (A-D) Softmax,(E-H) Sigmoid, and

4A

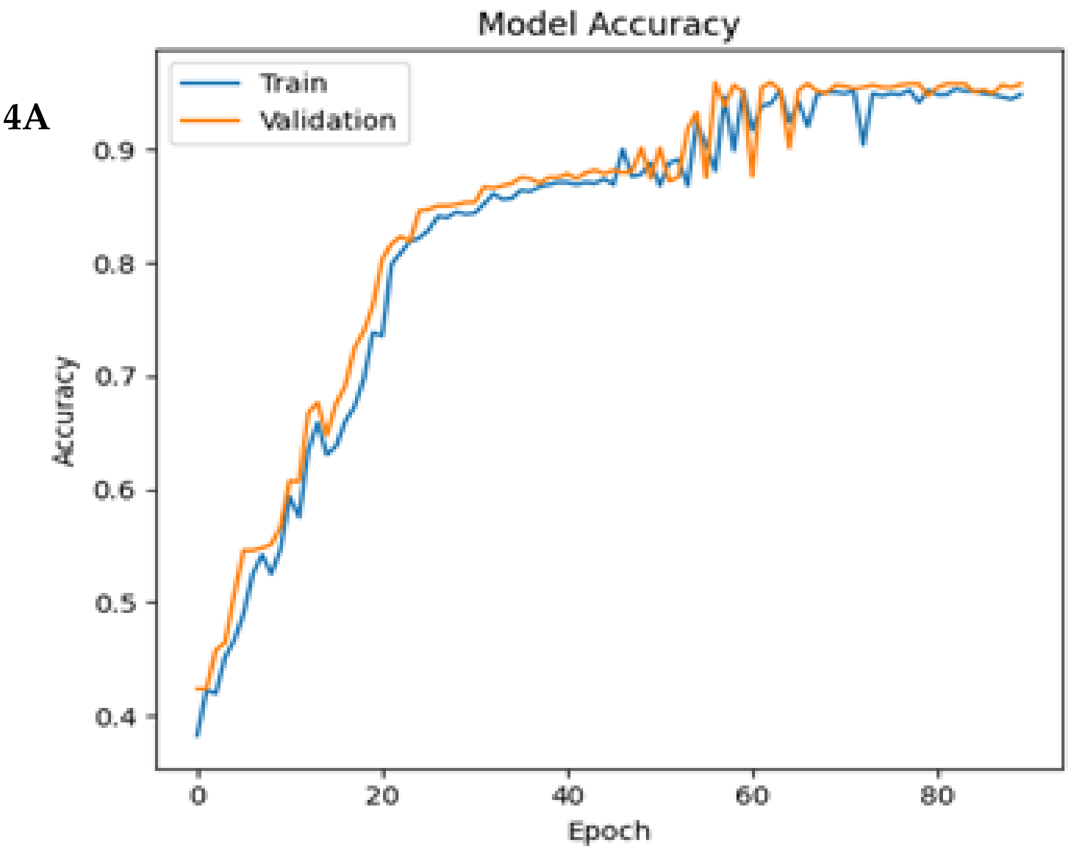

4B

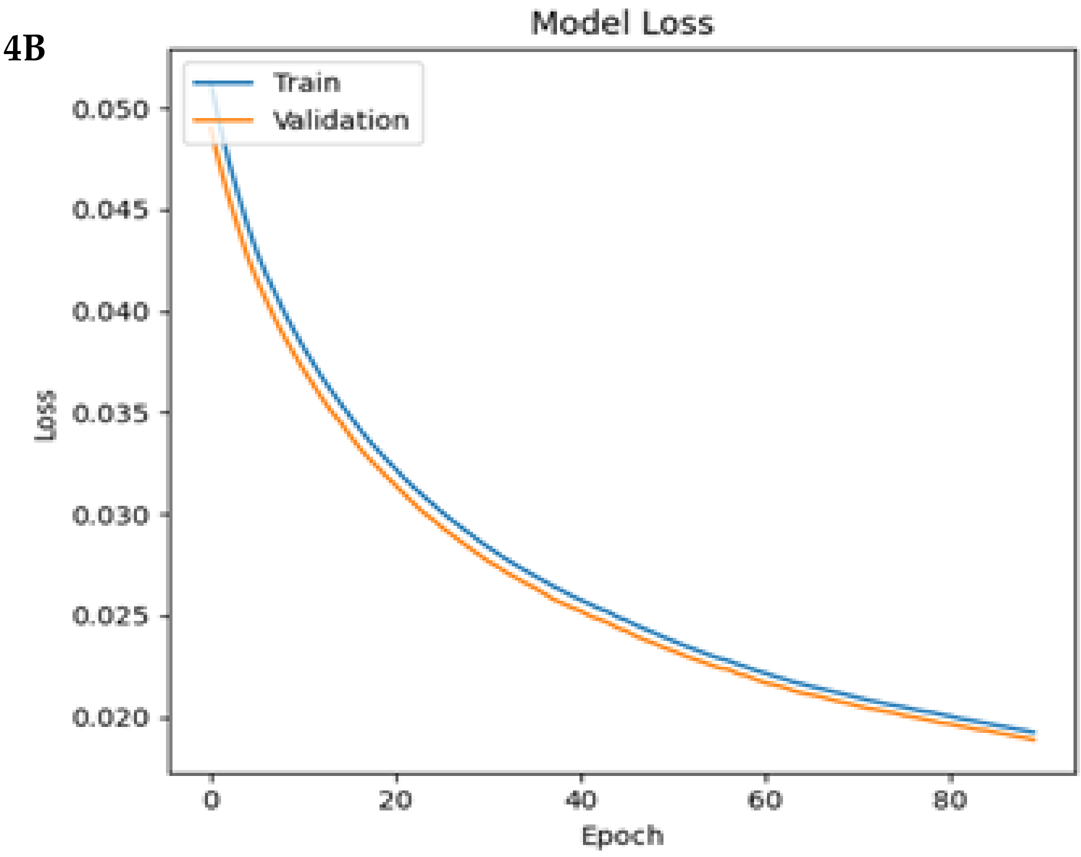

4C

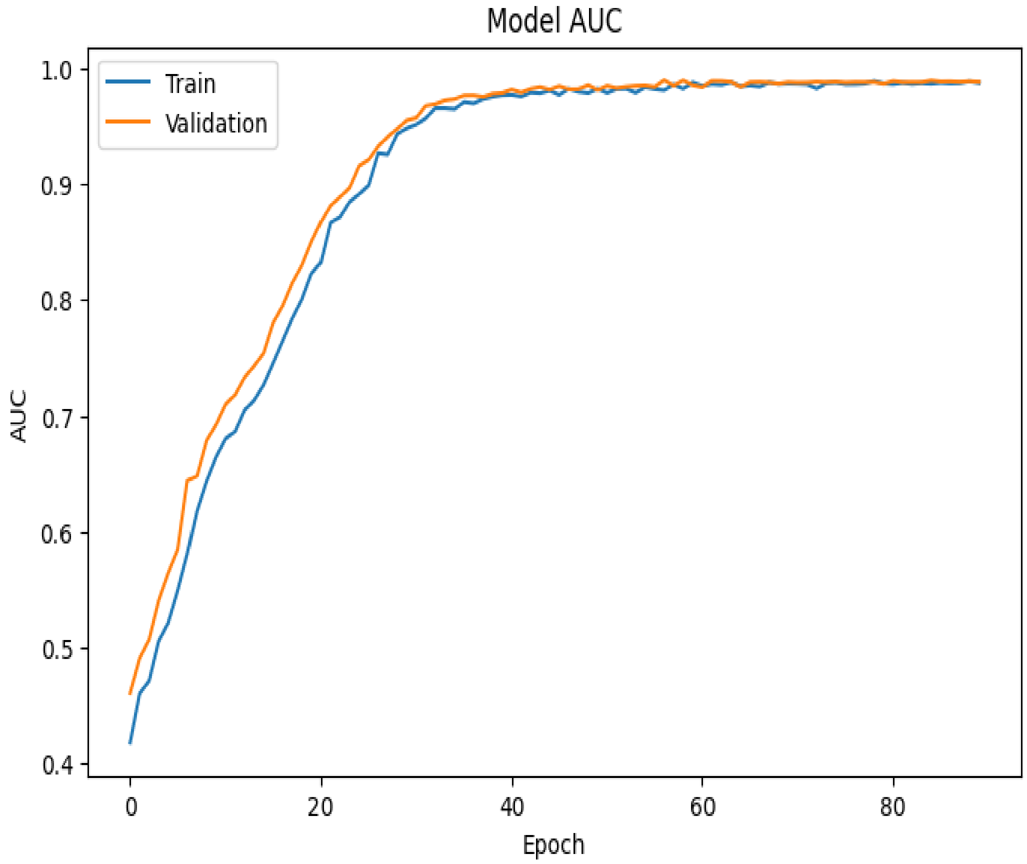
Model AUC
Train
Validation
AUC
Epoch
1.0
0.9
0.8
0.7
0.6
0.5
0.4
0
20
40
60
80

4D

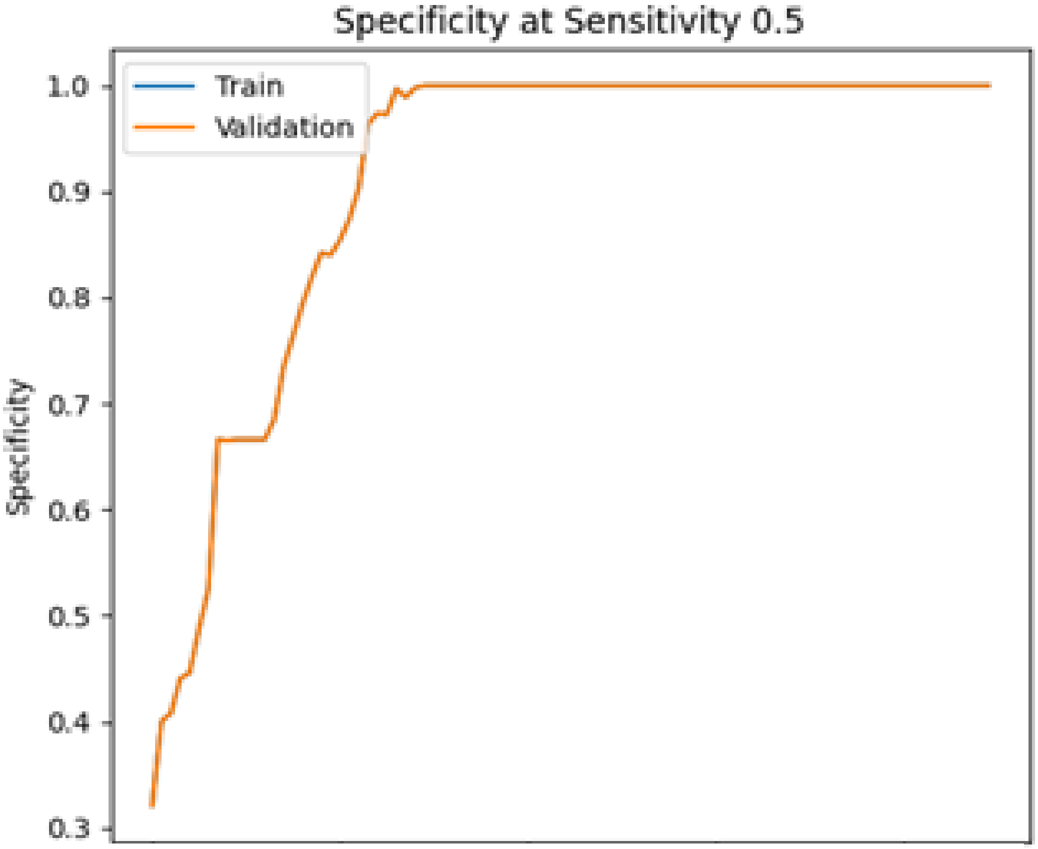
Specificity at Sensitivity 0.5
Train
Validation
Specificity
1.0
0.9
0.8
0.7
0.6
0.5
0.4
0.3

4E

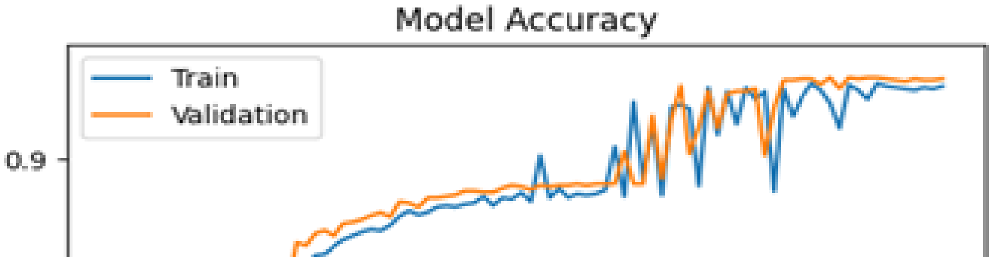
Model Accuracy
Train
Validation
0.9

4G

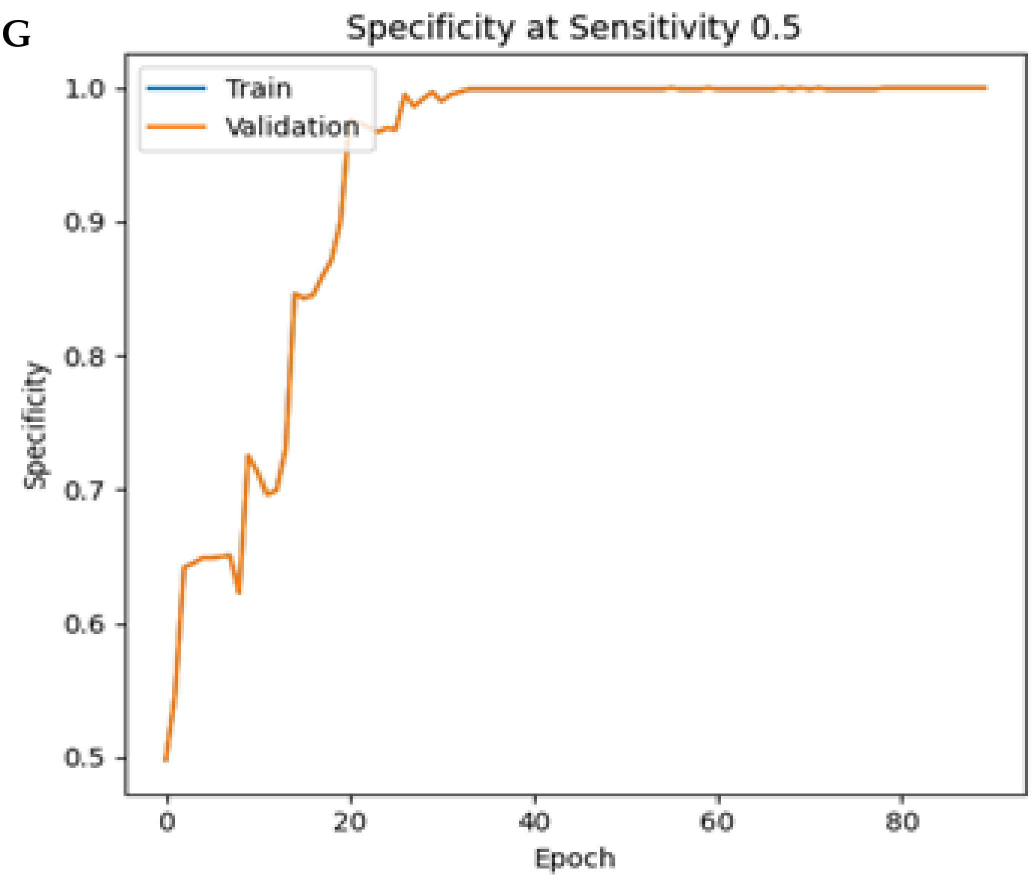
Specificity at Sensitivity 0.5
Train
Validation
1.0
0.9
0.8
0.7
0.6
0.5
Specificity
0
20
40
60
80
Epoch

4H

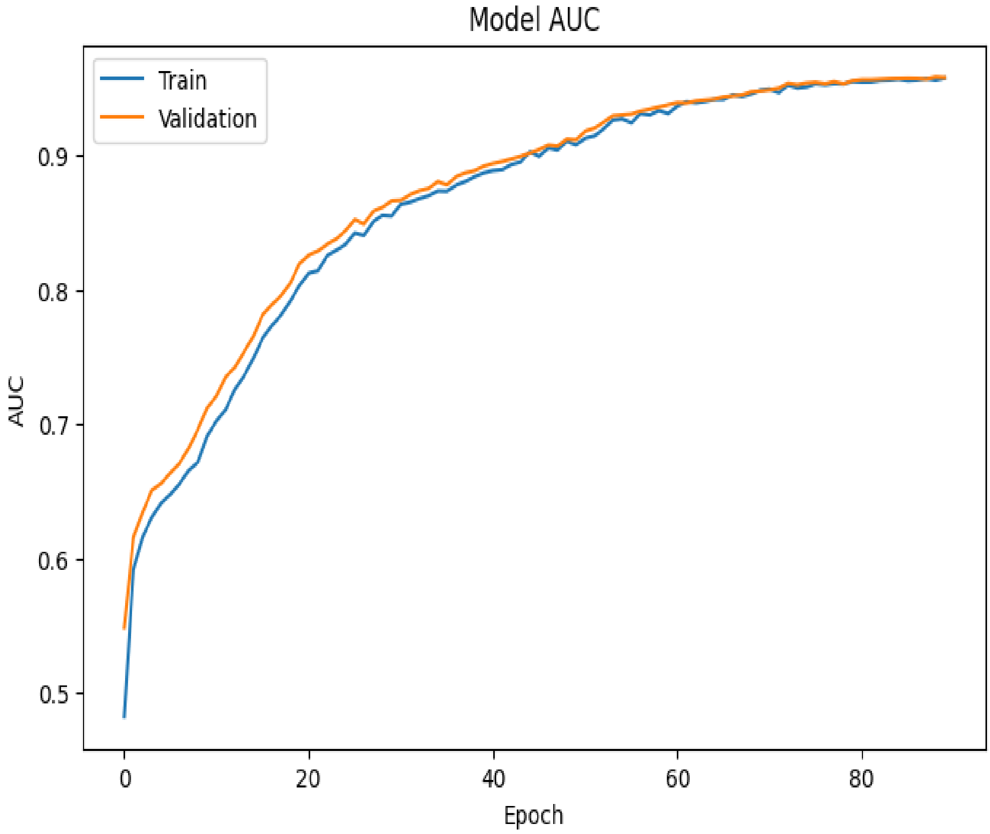
Model AUC
Train
Validation
0.9
0.8
0.7
0.6
0.5
AUC
0
20
40
60
80
Epoch

4I

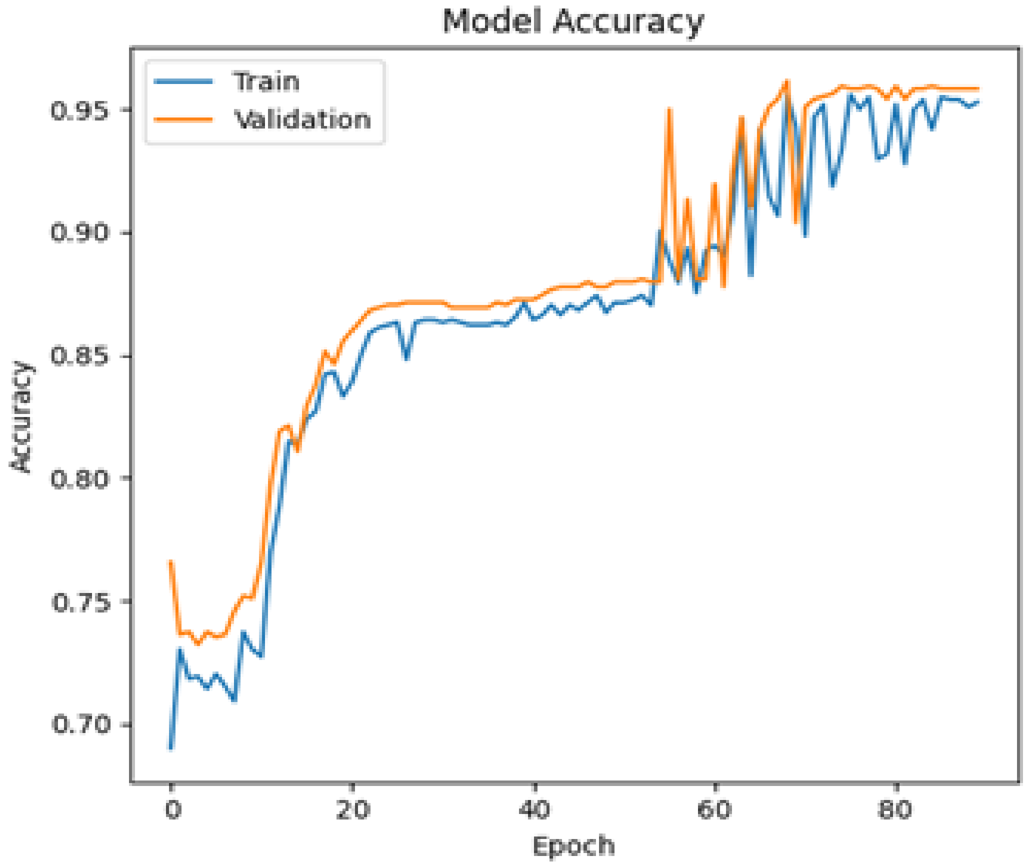
Model Accuracy
Train
Validation
Accuracy
0.95
0.90
0.85
0.80
0.75
0.70
0
20
40
60
80
Epoch

4J

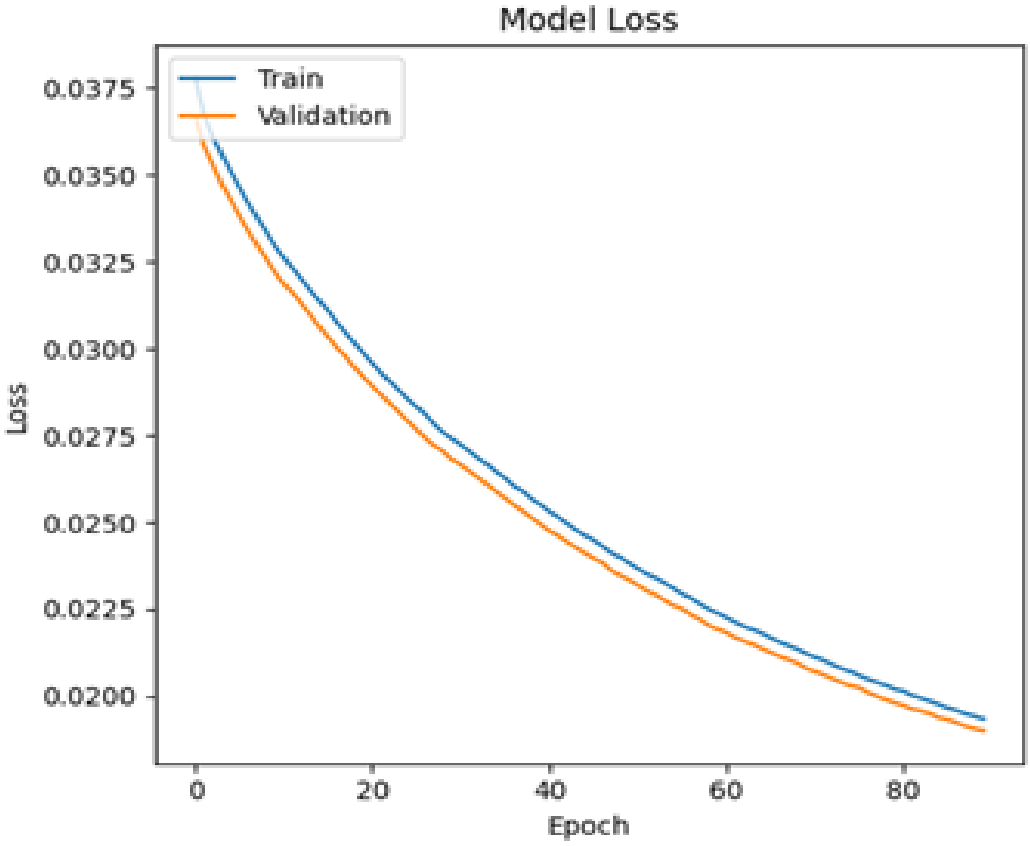
Model Loss
Train
Validation
Loss
0.0375
0.0350
0.0325
0.0300
0.0275
0.0250
0.0225
0.0200
0
20
40
60
80
Epoch

4K

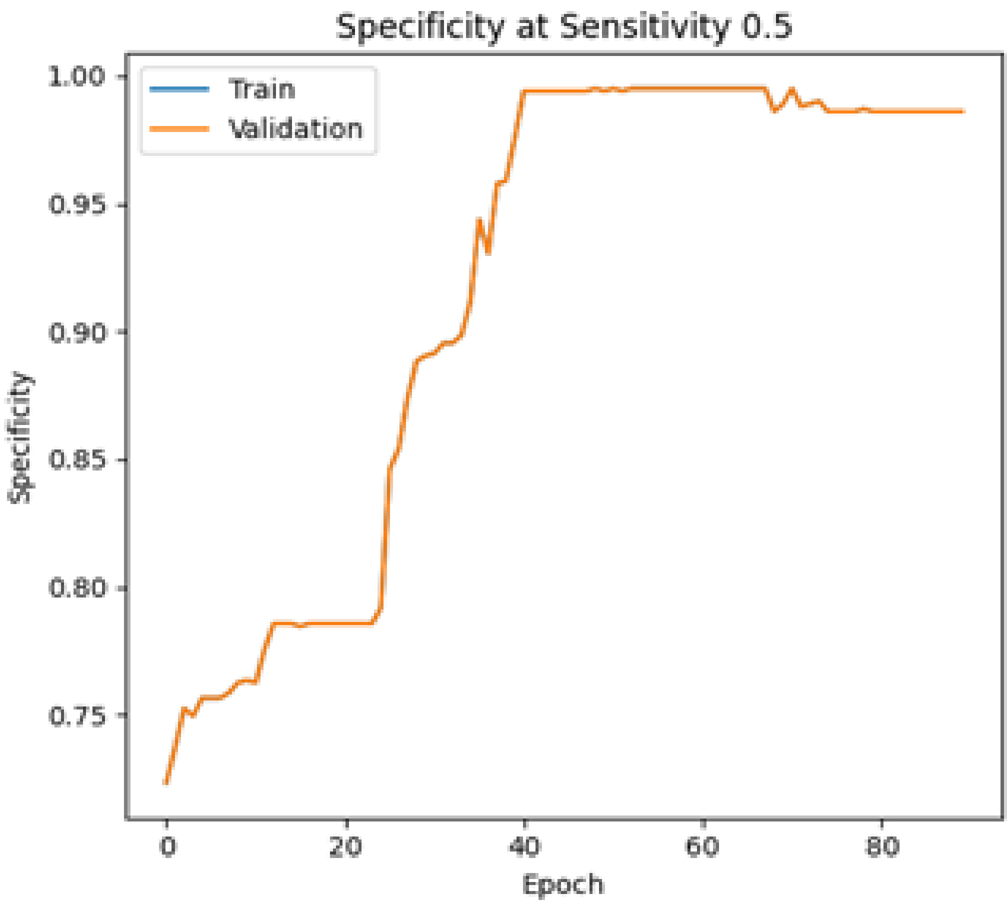
Specificity at Sensitivity 0.5
Train
Validation
Specificity
1.00
0.95
0.90
0.85
0.80
0.75
0
20
40
60
80
Epoch

4L

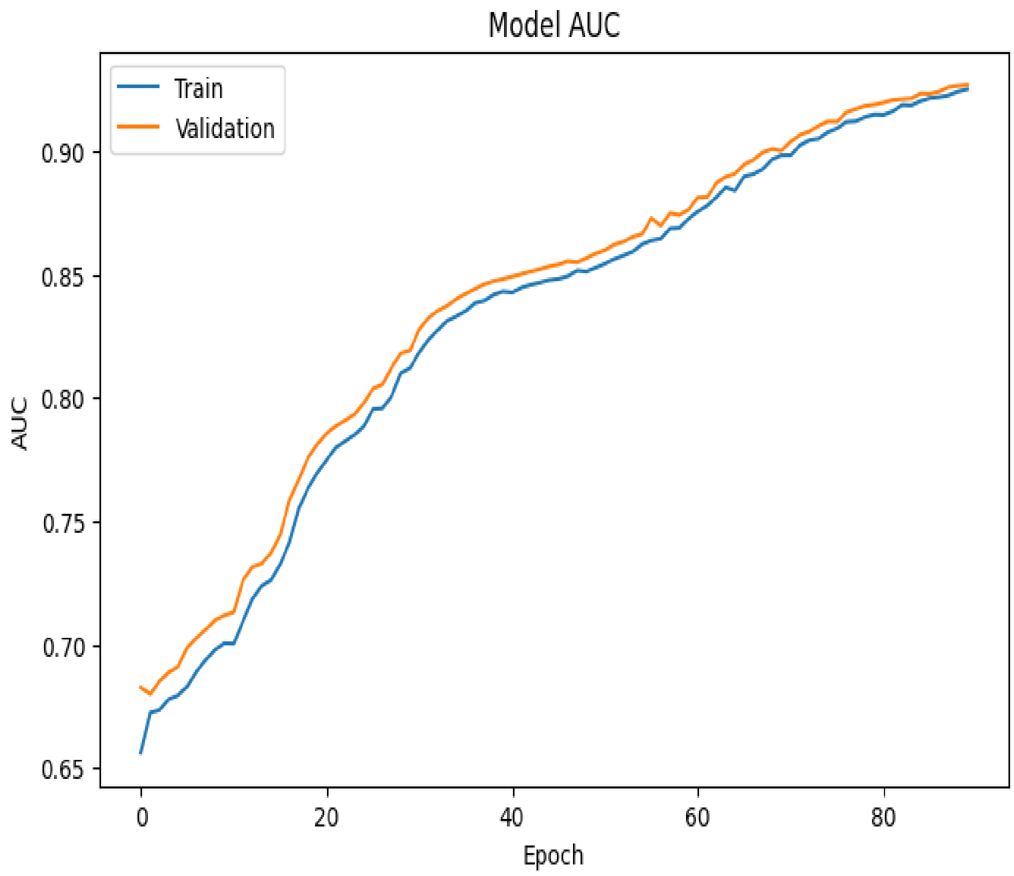
Model AUC
Train
Validation
AUC
0.90
0.85
0.80
0.75
0.70
0.65
0
20
40
60
80
Epoch

Figure S5. Classification of digestive system cancers. (A-D) Softmax,(E-H) Sigmoid, and (I-L)

5A

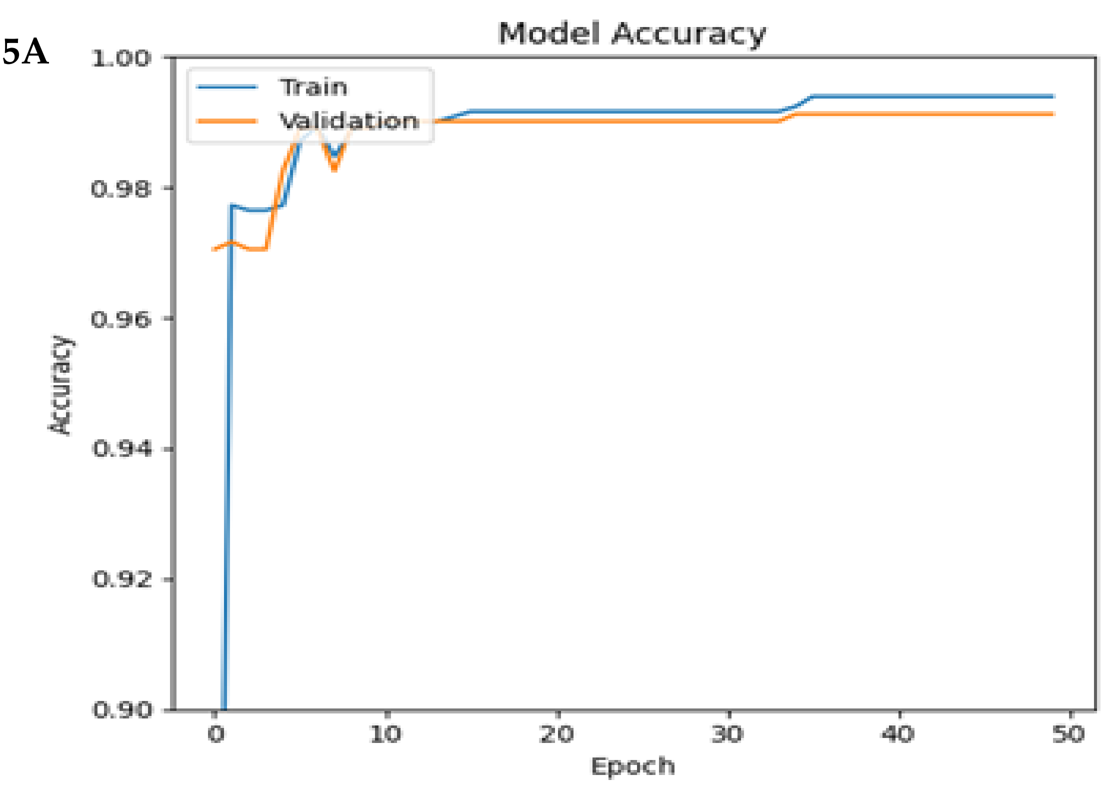

5B

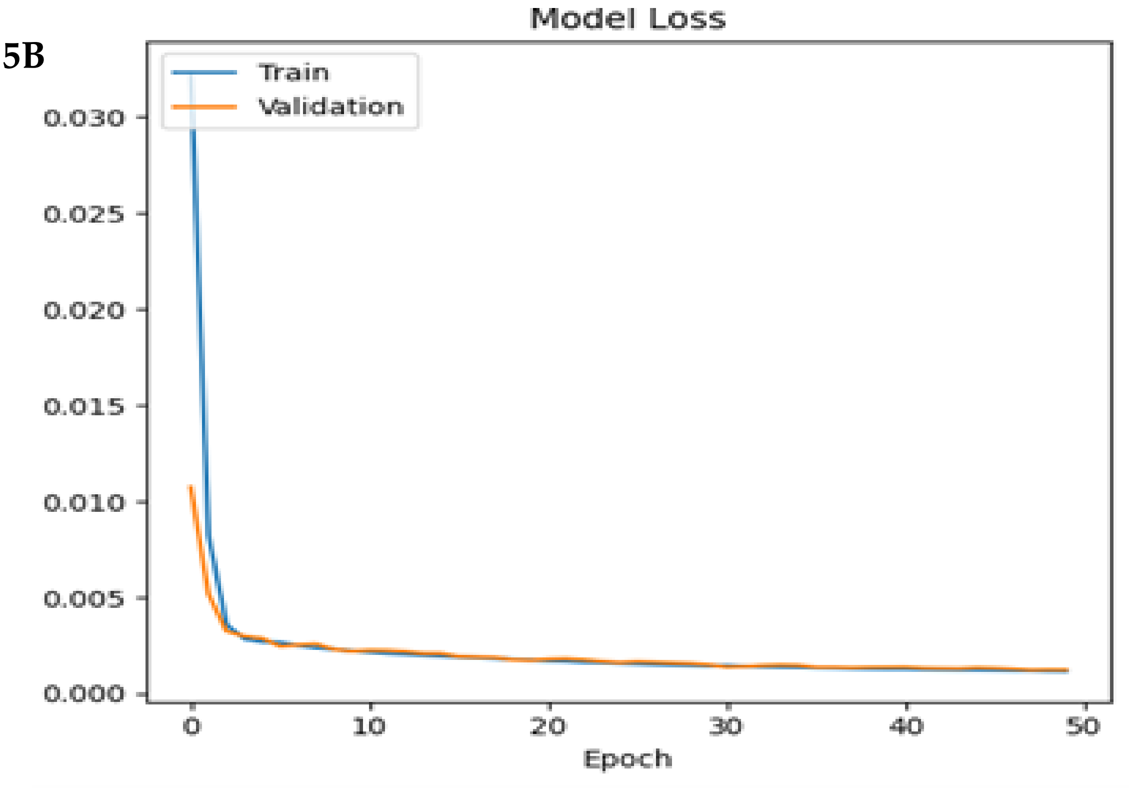

5C

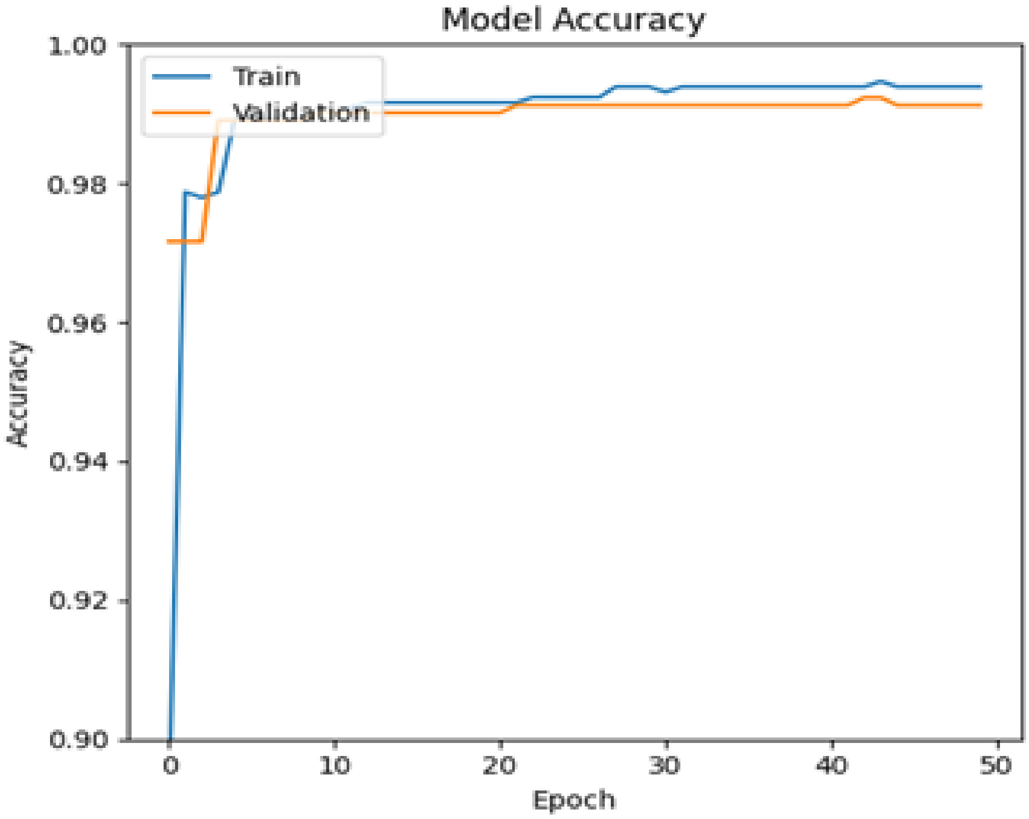
Model Accuracy
Train
Validation
Accuracy
1.00
0.98
0.96
0.94
0.92
0.90
0
10
20
30
40
50
Epoch

5D

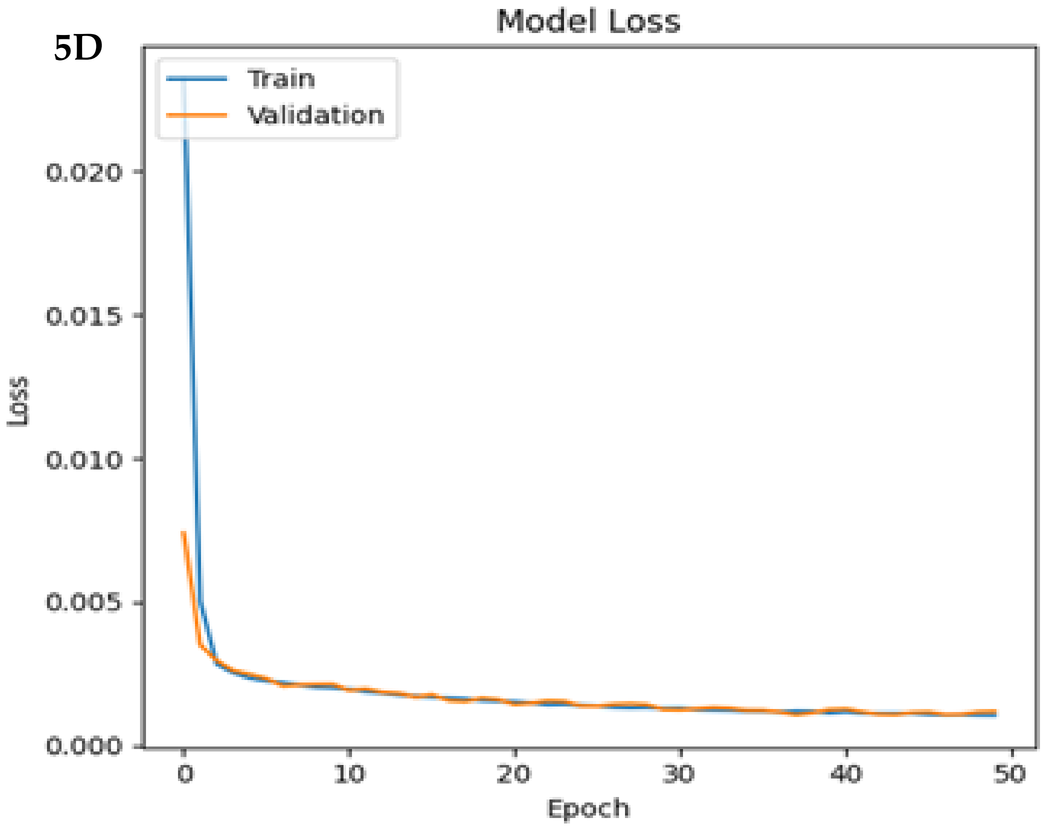
Model Loss
Train
Validation
Loss
0.020
0.015
0.010
0.005
0.000
0
10
20
30
40
50
Epoch

5E

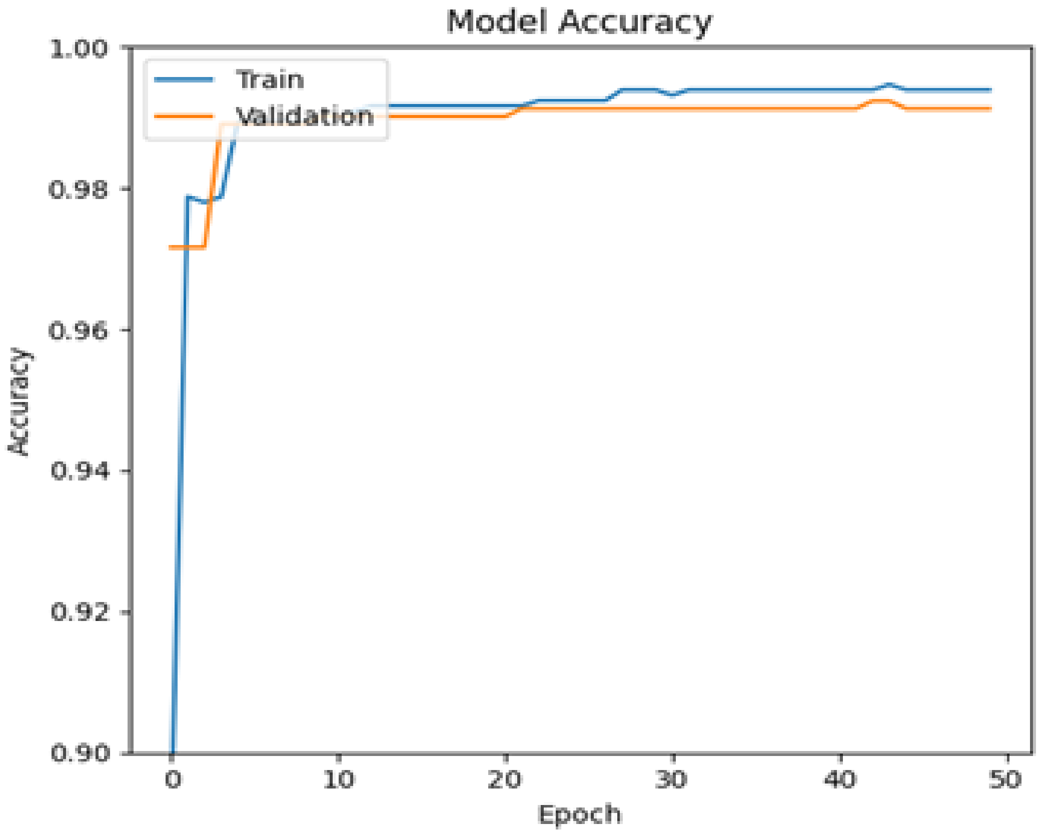
Model Accuracy
Train
Validation
1.00
0.98
0.96
0.94
0.92
0.90
Accuracy
0
10
20
30
40
50
Epoch

5F

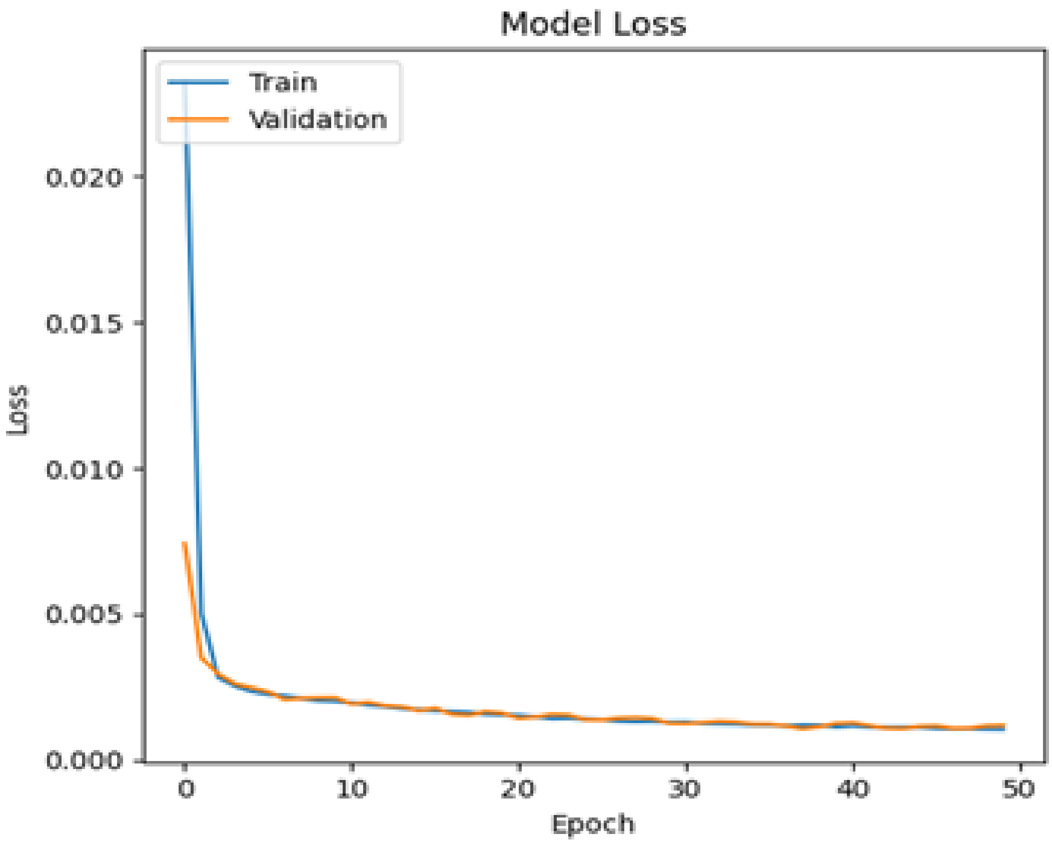
Model Loss
Train
Validation
0.020
0.015
0.010
0.005
0.000
Loss
0
10
20
30
40
50
Epoch

5G

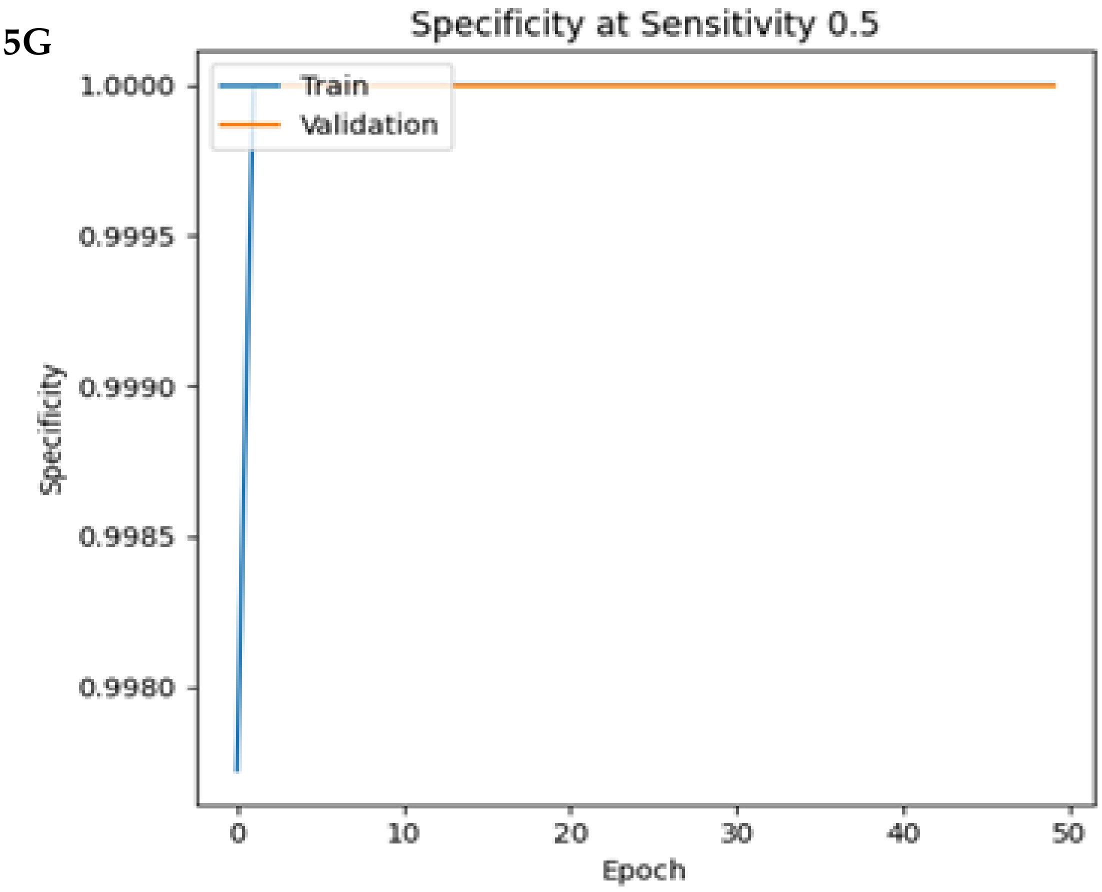
Specificity at Sensitivity 0.5
Train
Validation
1.0000
0.9995
0.9990
0.9985
0.9980
Specificity
0
10
20
30
40
50
Epoch

5H

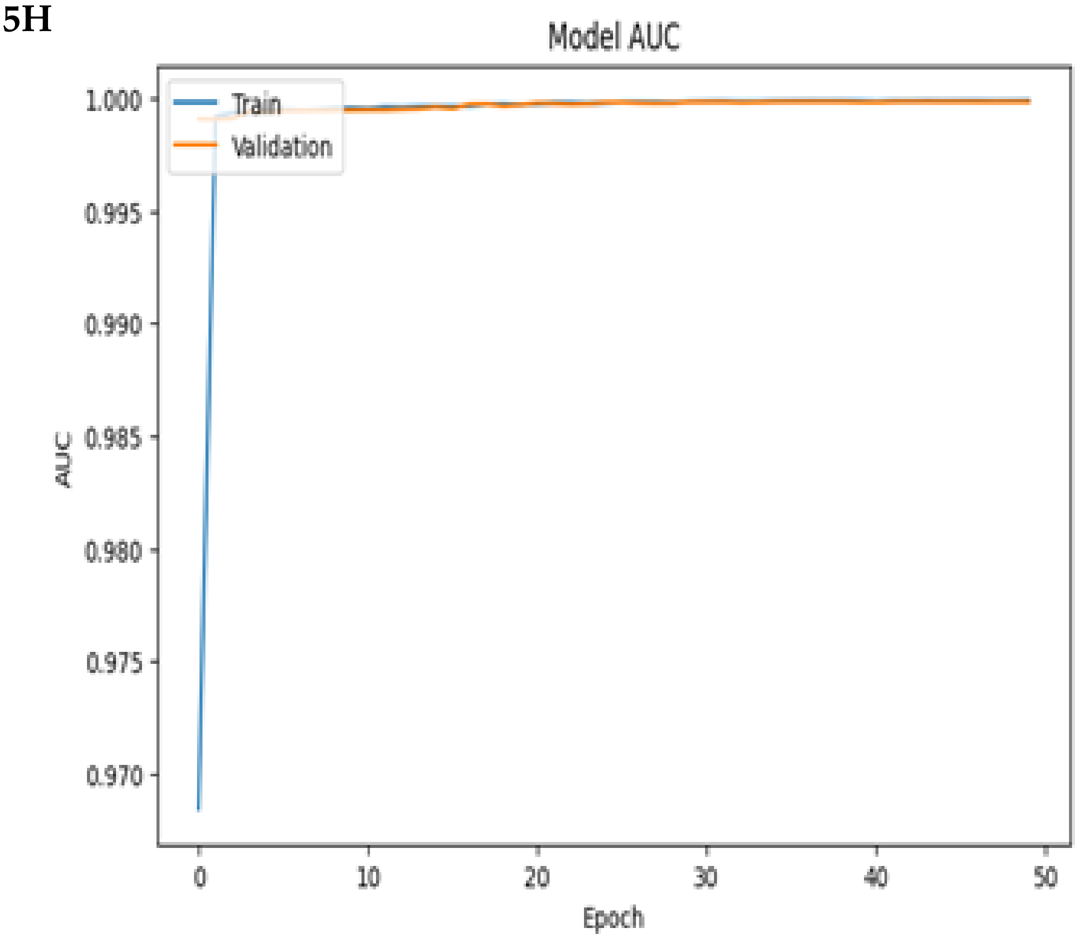
Model AUC
Train
Validation
1.000
0.995
0.990
0.985
0.980
0.975
0.970
AUC
0
10
20
30
40
50
Epoch

5I

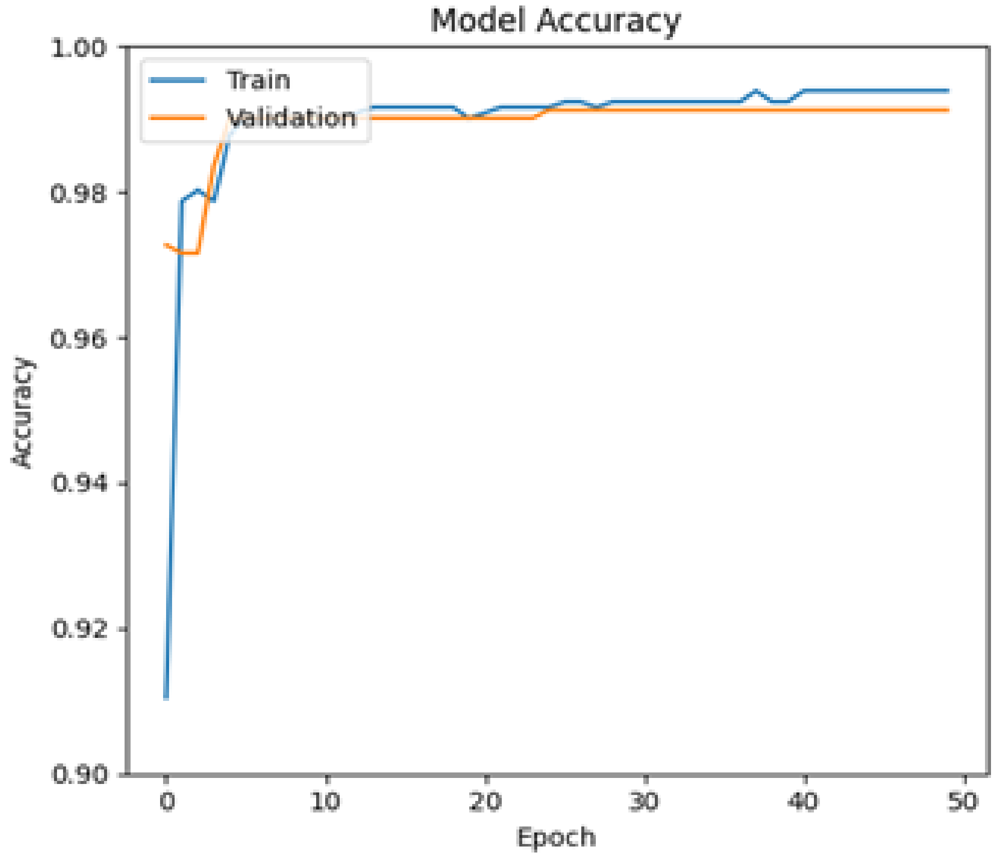
Model Accuracy
Train
Validation
Accuracy
Epoch
1.00
0.98
0.96
0.94
0.92
0.90
0
10
20
30
40
50

5J

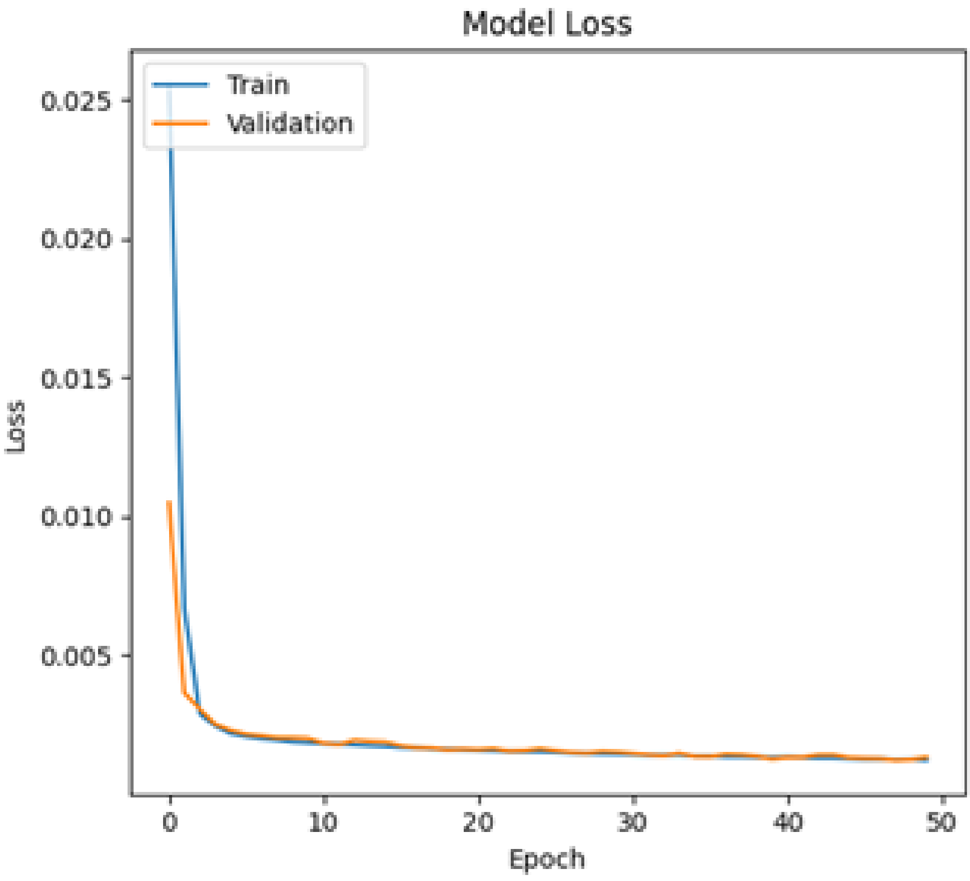
Model Loss
Train
Validation
Loss
Epoch
0.025
0.020
0.015
0.010
0.005
0
10
20
30
40
50

5K

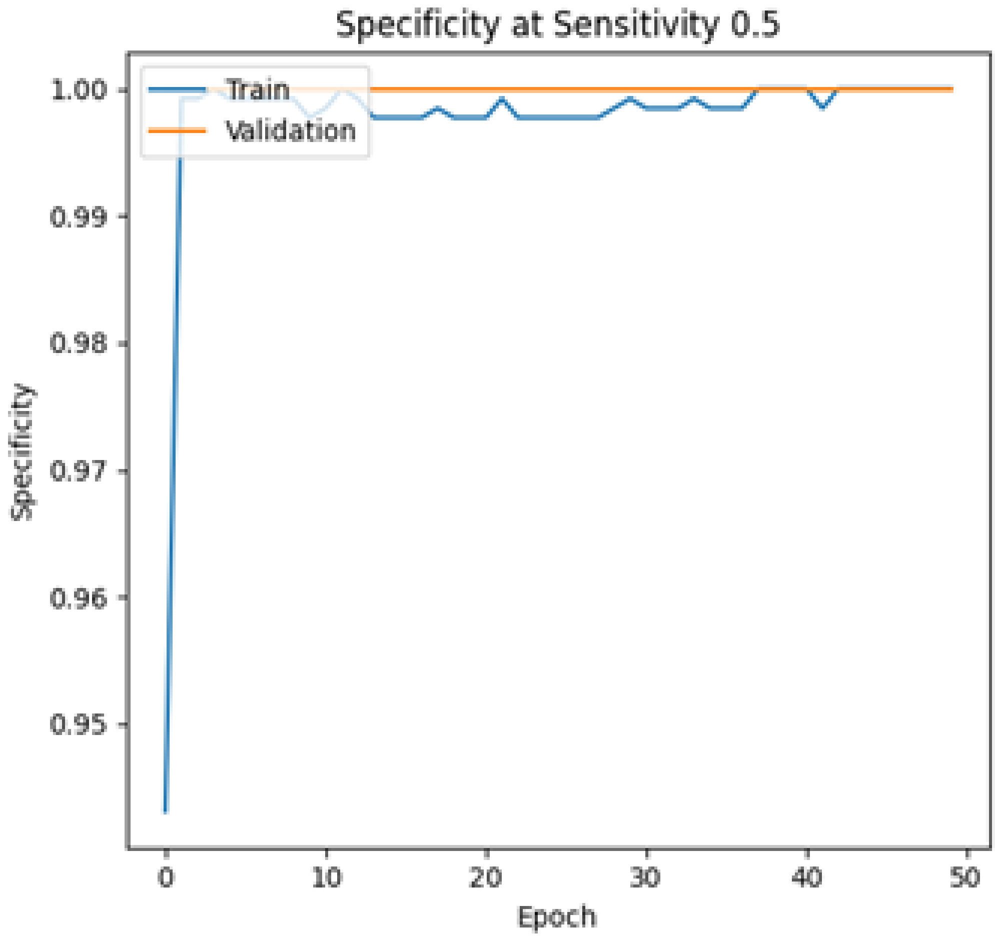
Specificity at Sensitivity 0.5
Train
Validation
1.00
0.99
0.98
0.97
0.96
0.95
Specificity
0
10
20
30
40
50
Epoch

5L

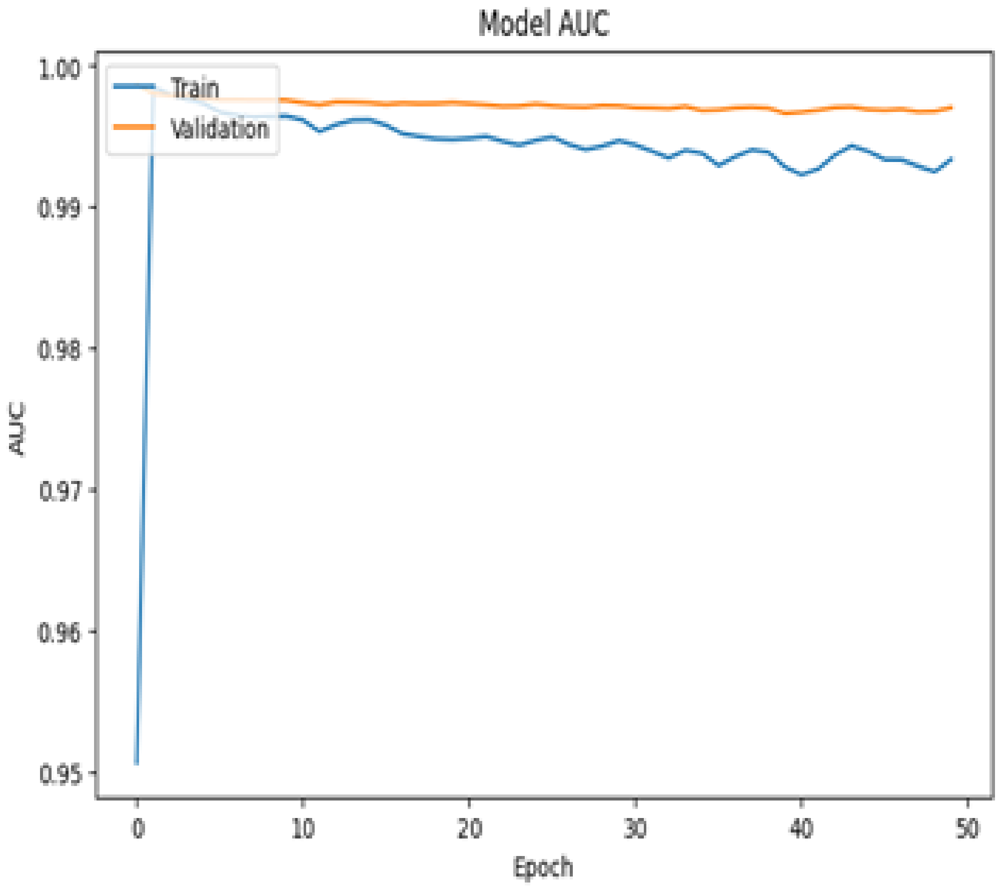
Model AUC
Train
Validation
1.00
0.99
0.98
0.97
0.96
0.95
AUC
0
10
20
30
40
50
Epoch

Figure S6. Classification of digestive system cancers. (A-D) Softmax,(E-H) Sigmoid, and (I-L)

6A

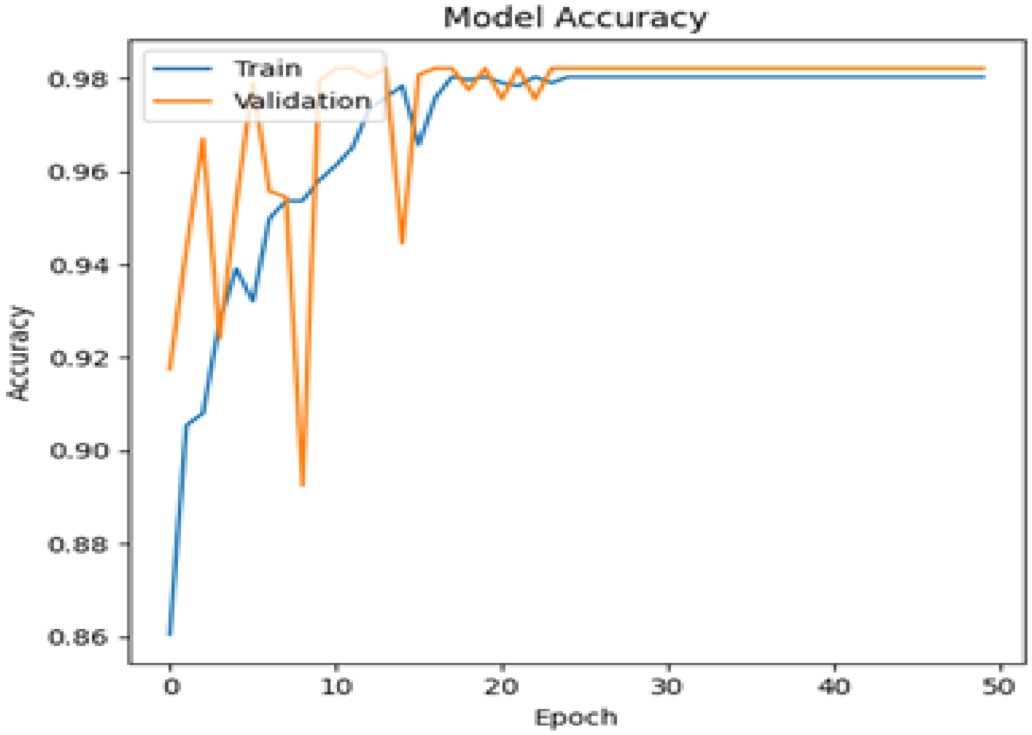

6B

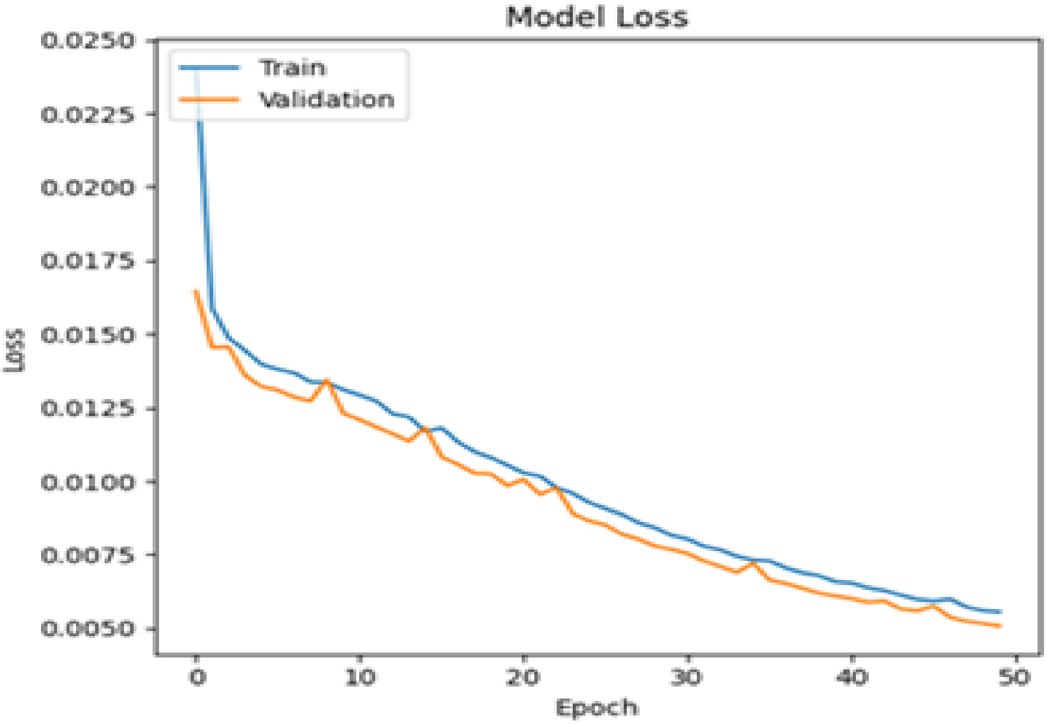

6C

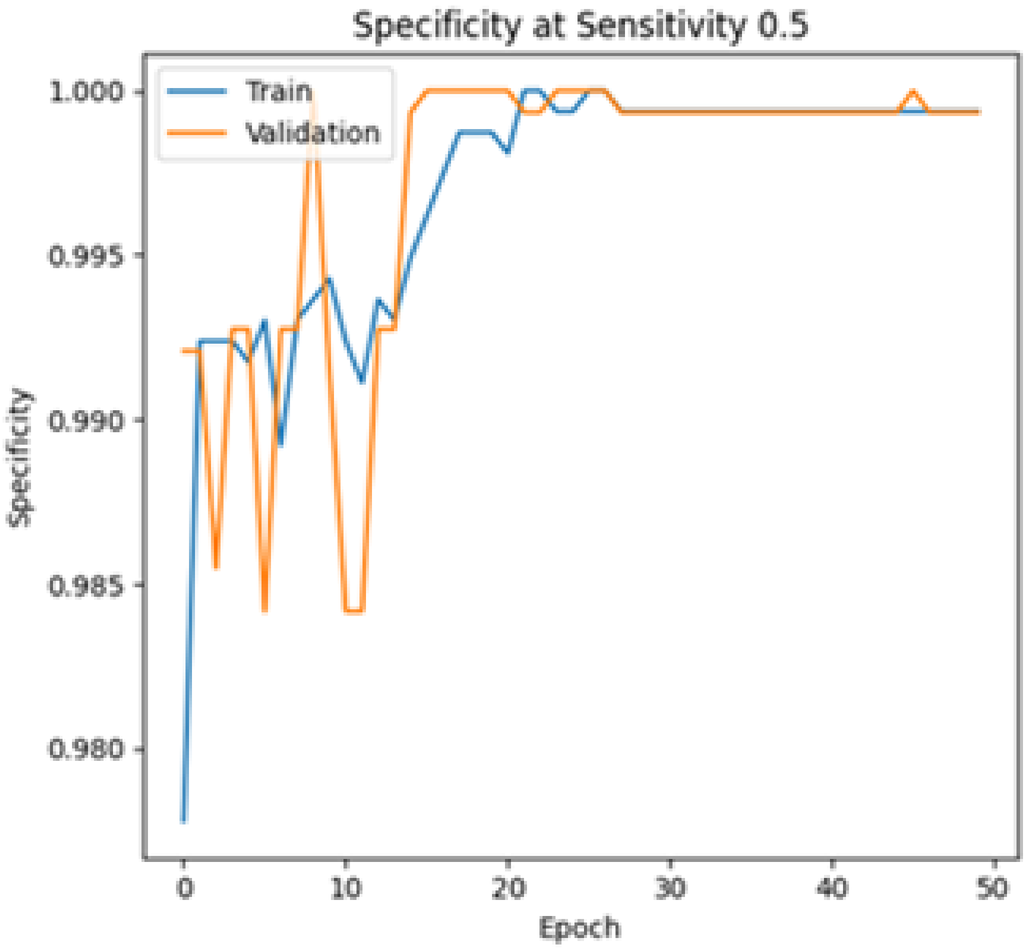
Specificity at Sensitivity 0.5
Train
Validation
1.000
0.995
0.990
0.985
0.980
Specificity
0
10
20
30
40
50
Epoch

6D

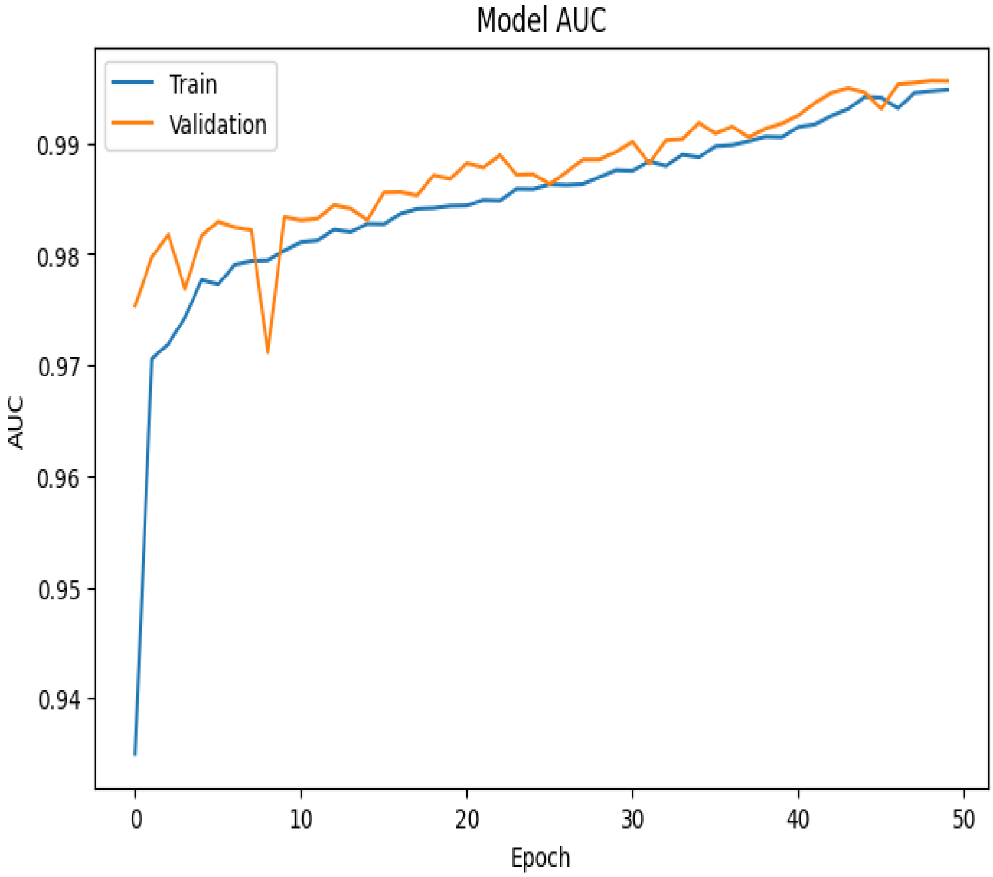
Model AUC
Train
Validation
0.99
0.98
0.97
0.96
0.95
0.94
AUC
0
10
20
30
40
50
Epoch

6E

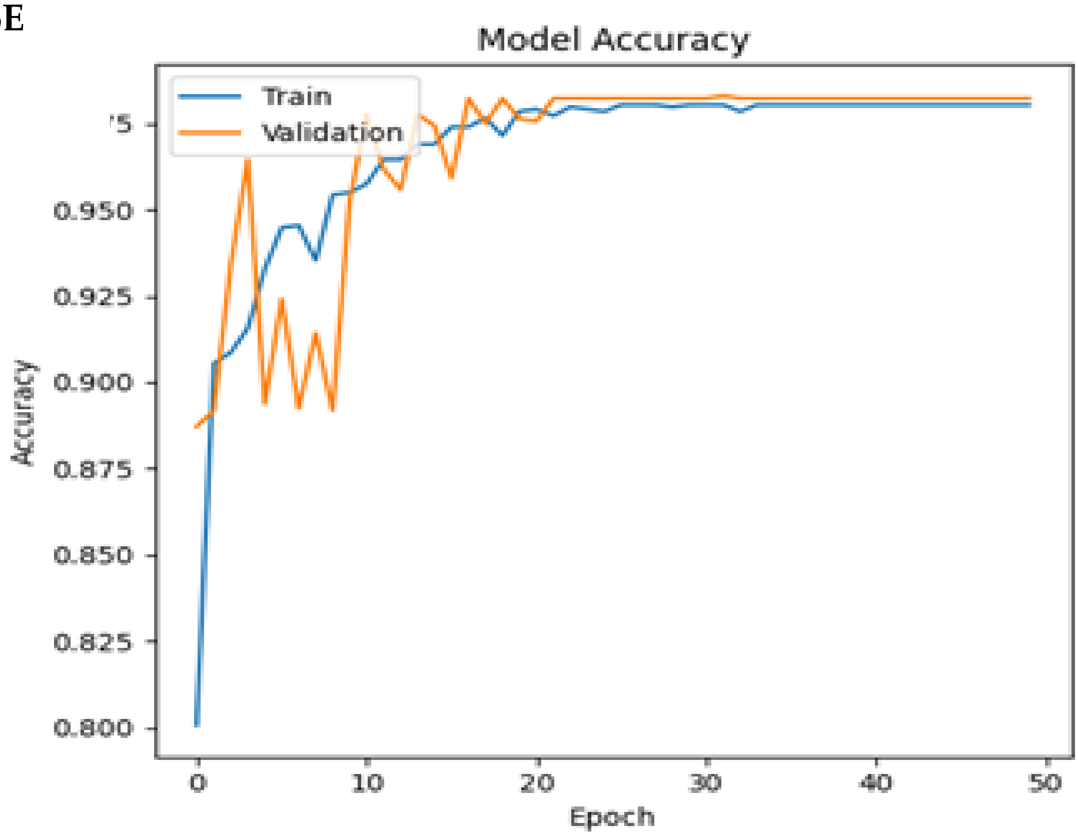
Model Accuracy
Train
Validation
'5
0.950
0.925
0.900
0.875
0.850
0.825
0.800
Accuracy
0
10
20
30
40
50
Epoch

6F

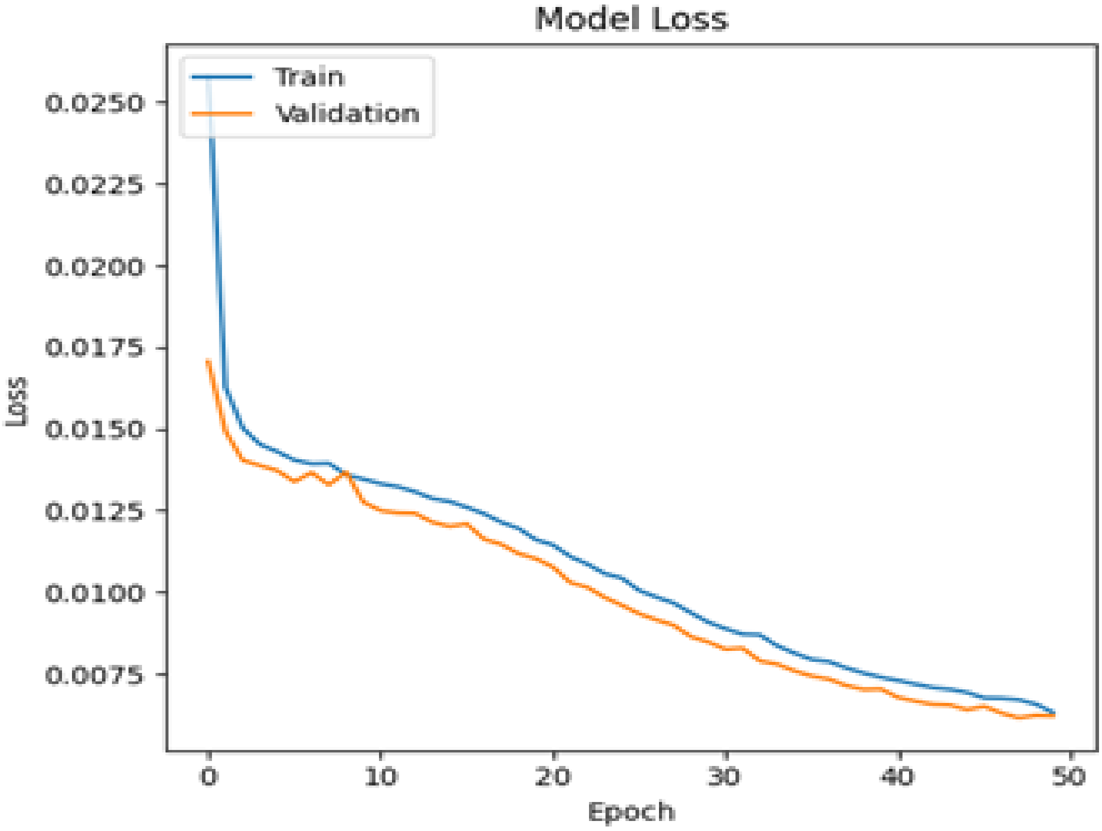
Model Loss
Train
Validation
0.0250
0.0225
0.0200
0.0175
0.0150
0.0125
0.0100
0.0075
Loss
0
10
20
30
40
50
Epoch

6G

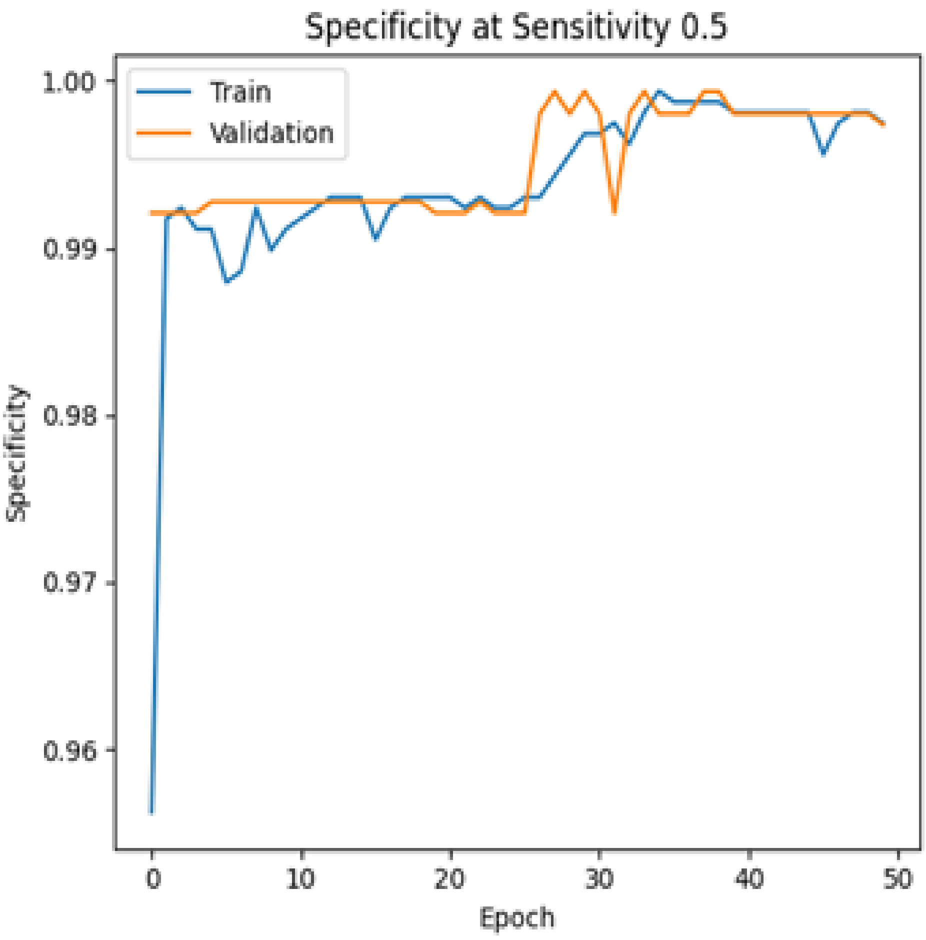
Specificity at Sensitivity 0.5
Train
Validation
1.00
0.99
0.98
0.97
0.96
Specificity
0
10
20
30
40
50
Epoch

6H

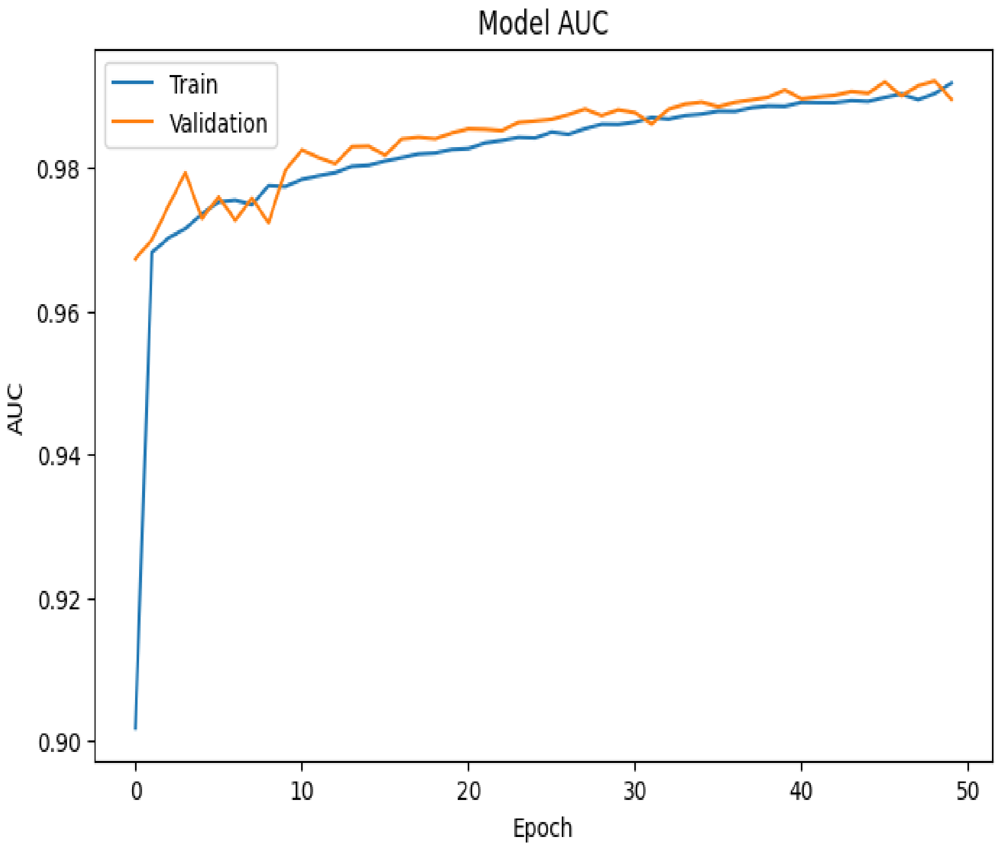
Model AUC
Train
Validation
0.98
0.96
0.94
0.92
0.90
AUC
0
10
20
30
40
50
Epoch

6I

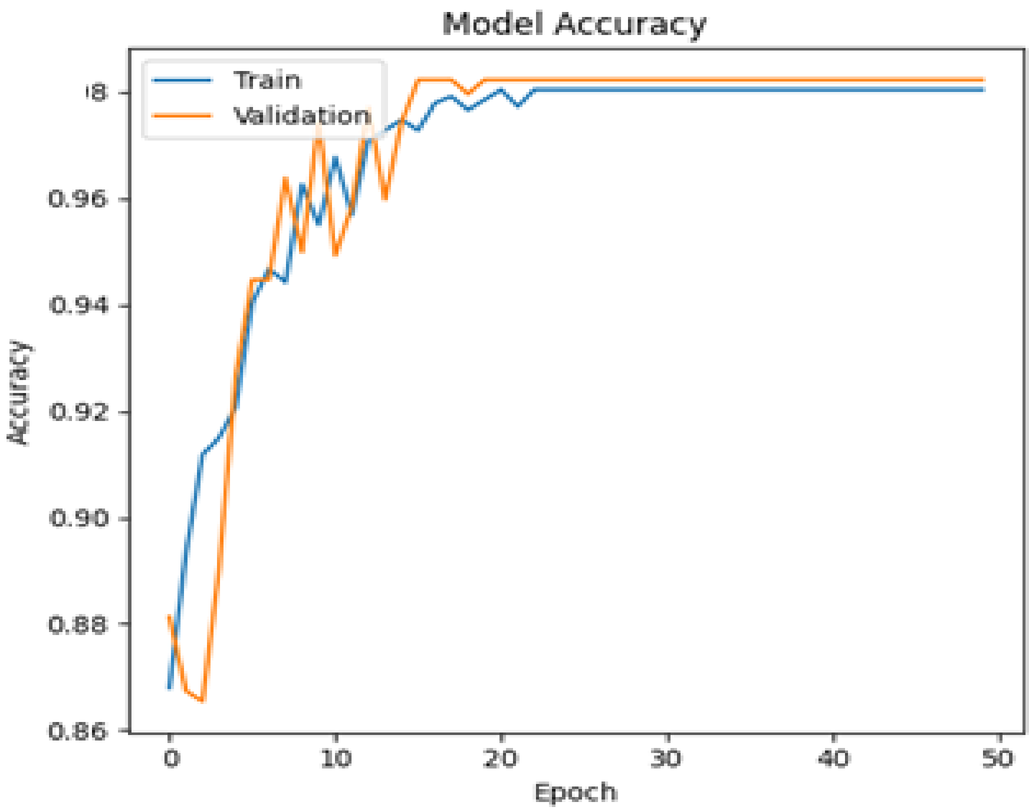
Model Accuracy
Train
Validation
Accuracy
0.86
0.88
0.90
0.92
0.94
0.96
0
10
20
30
40
50
Epoch

6J

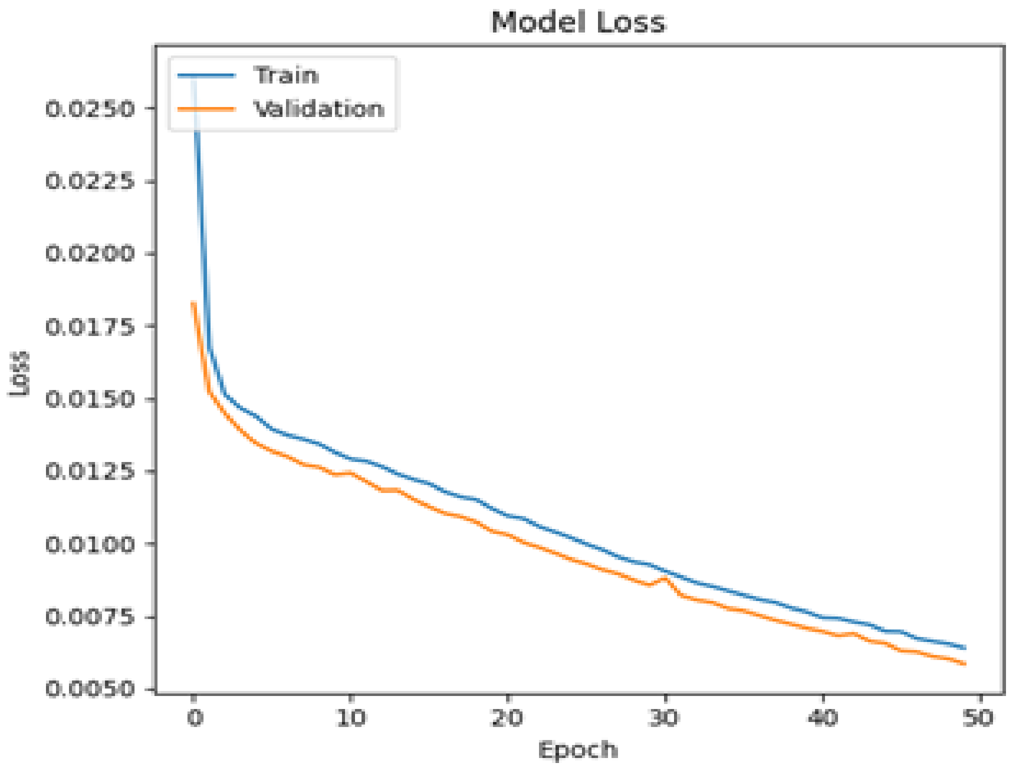
Model Loss
Train
Validation
Loss
0.0050
0.0075
0.0100
0.0125
0.0150
0.0175
0.0200
0.0225
0.0250
0
10
20
30
40
50
Epoch

6K

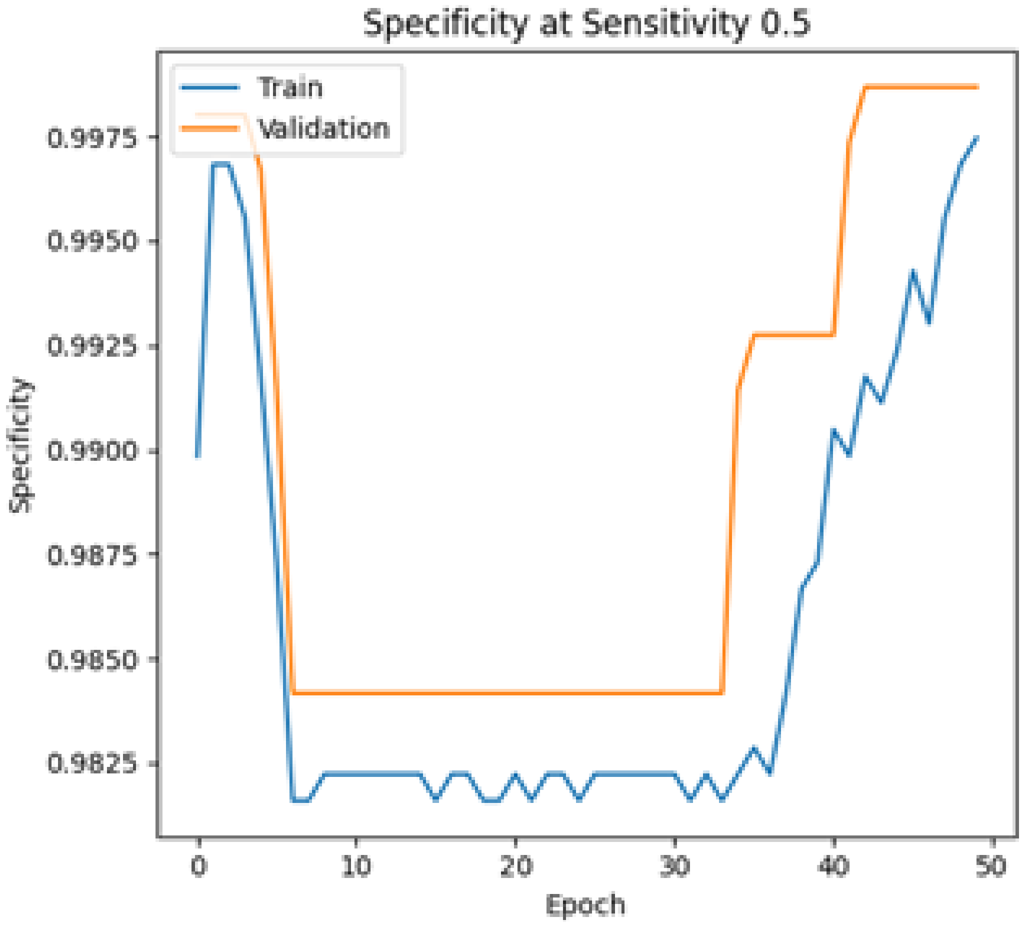
Specificity at Sensitivity 0.5
Train
Validation
0.9975
0.9950
0.9925
0.9900
0.9875
0.9850
0.9825
Specificity
0
10
20
30
40
50
Epoch

6L

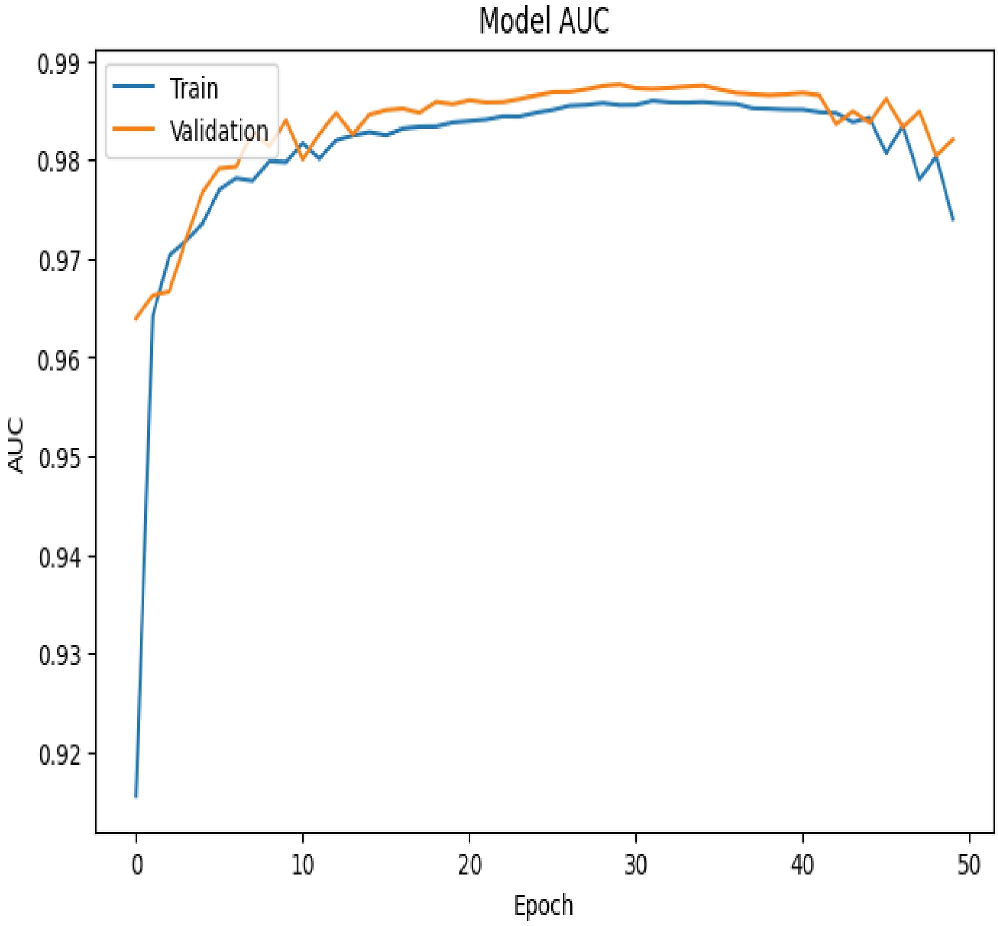
Model AUC
Train
Validation
0.99
0.98
0.97
0.96
0.95
0.94
0.93
0.92
AUC
0
10
20
30
40
50
Epoch

Figure S7. Biological explainability analysis of the generalized CNN model performance.

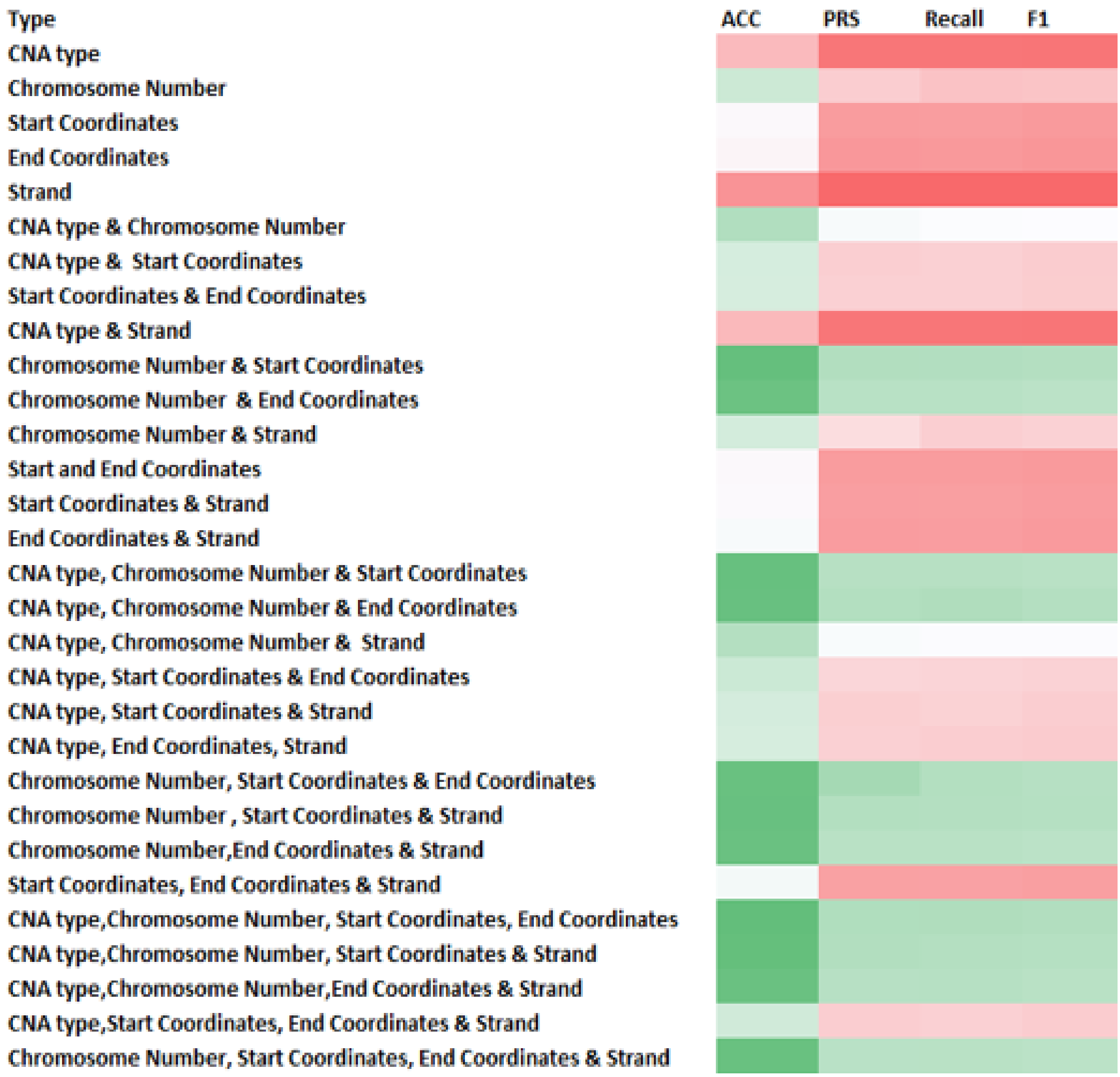

Supplement: Supplementary file 1 [file cimb-46-00490-s001.zip › cimb-3043040-supplementary.pdf]
